# Bioprospecting *Trichoderma*: A Systematic Roadmap to Screen Genomes and Natural Products for Biocontrol Applications

**DOI:** 10.3389/ffunb.2021.716511

**Published:** 2021-09-16

**Authors:** Tomás A. Rush, Him K. Shrestha, Muralikrishnan Gopalakrishnan Meena, Margaret K. Spangler, J. Christopher Ellis, Jesse L. Labbé, Paul E. Abraham

**Affiliations:** ^1^Oak Ridge National Laboratory, Biosciences Division, Oak Ridge, TN, United States; ^2^Graduate School of Genome Science and Technology, University of Tennessee, Knoxville, Knoxville, TN, United States; ^3^Oak Ridge National Laboratory, National Center for Computational Sciences, Oak Ridge, TN, United States

**Keywords:** predictive biology, functional genomics, secondary metabolites, antimicrobials, integrated pest management, plant-microbe interactions, graph theory—graph algorithms, drug discoveries

## Abstract

Natural products derived from microbes are crucial innovations that would help in reaching sustainability development goals worldwide while achieving bioeconomic growth. *Trichoderma* species are well-studied model fungal organisms used for their biocontrol properties with great potential to alleviate the use of agrochemicals in agriculture. However, identifying and characterizing effective natural products in novel species or strains as biological control products remains a meticulous process with many known challenges to be navigated. Integration of recent advancements in various “omics” technologies, next generation biodesign, machine learning, and artificial intelligence approaches could greatly advance bioprospecting goals. Herein, we propose a roadmap for assessing the potential impact of already known or newly discovered *Trichoderma* species for biocontrol applications. By screening publicly available *Trichoderma* genome sequences, we first highlight the prevalence of putative biosynthetic gene clusters and antimicrobial peptides among genomes as an initial step toward predicting which organisms could increase the diversity of natural products. Next, we discuss high-throughput methods for screening organisms to discover and characterize natural products and how these findings impact both fundamental and applied research fields.

## Introduction

The discovery and usage of biological controls as management strategies started with astute observations of ecological niches for studying microbial interactions (Dubos, 1939; Dubos and Cattaneo, 1939; van den Bosch et al., 1982; Dias et al., 2012; Barratt et al., 2018). Later came the rise of biological control applications due to a consortium of scientists and industry partners working on multidisciplinary ideas and projects that initially seemed unrelated yet were serving a common goal. Invasive species cost around $120 billion USD yearly in crop yield losses (Pimentel et al., 2005); plant pathogens alone, primarily fungi, result in annual crop financial losses estimated at $23.5 billion USD (including control costs) (Rossman, 2009). Even more alarming are the pesticide-resistant populations that exist, which have led to the conception of several organizations, e.g., the Fungicide Resistance Action Committee (https://www.frac.info/), and the Insecticide Resistance Action Committee (https://irac-online.org/). However, not all microbes associated with crops are harmful (Stark, 2010). In fact, beneficial microbes have become an integral component of pest management strategies to control pest populations or promote plant health (Meena et al., 2017). Factors that influence the use of beneficial microbes as biological control products are stress-induced environments, nutrient-deficient areas, and known populations of plant pathogens that can be controlled (Hayat et al., 2010; Stark, 2010; Chen et al., 2018; Begum et al., 2019; Kulimushi et al., 2021). In general, the use of biological control products is preferred for many reasons, including the reduction of pesticide use, cost-effectiveness, and its efficacy against a broad range of natural pest and support services (Bale et al., 2008; Benjamin and Wesseler, 2016; Barratt et al., 2018). Yet, biological control product applications face several challenges including invasive species stemming from the fungus used as an active ingredient; increasing crop groups, cultivars and varieties; pest complexes and resistances; incompatibility with pesticides; non-targeted effects, and risk assessment strategies (Bale et al., 2008; Barratt et al., 2018; Köhl et al., 2019). Given the complexity of these challenges, herein we propose a roadmap for bioprospecting microbes, using *Trichoderma* species as our model organisms. The suggested roadmap integrates predictive biology, functional genomics, high-throughput analytics, and next-generation biodesign and genome engineering approaches. Moreover, because newly discovered and characterized biological controls can have various applications, we provide a summary of important advantages and drawbacks that should be considered. Using this framework, we begin to predict which species among the already sequenced *Trichoderma* have unique potential as valuable biocontrol agents or source of natural products.

### The Growing Market for Biological Products

**Figure 1:**
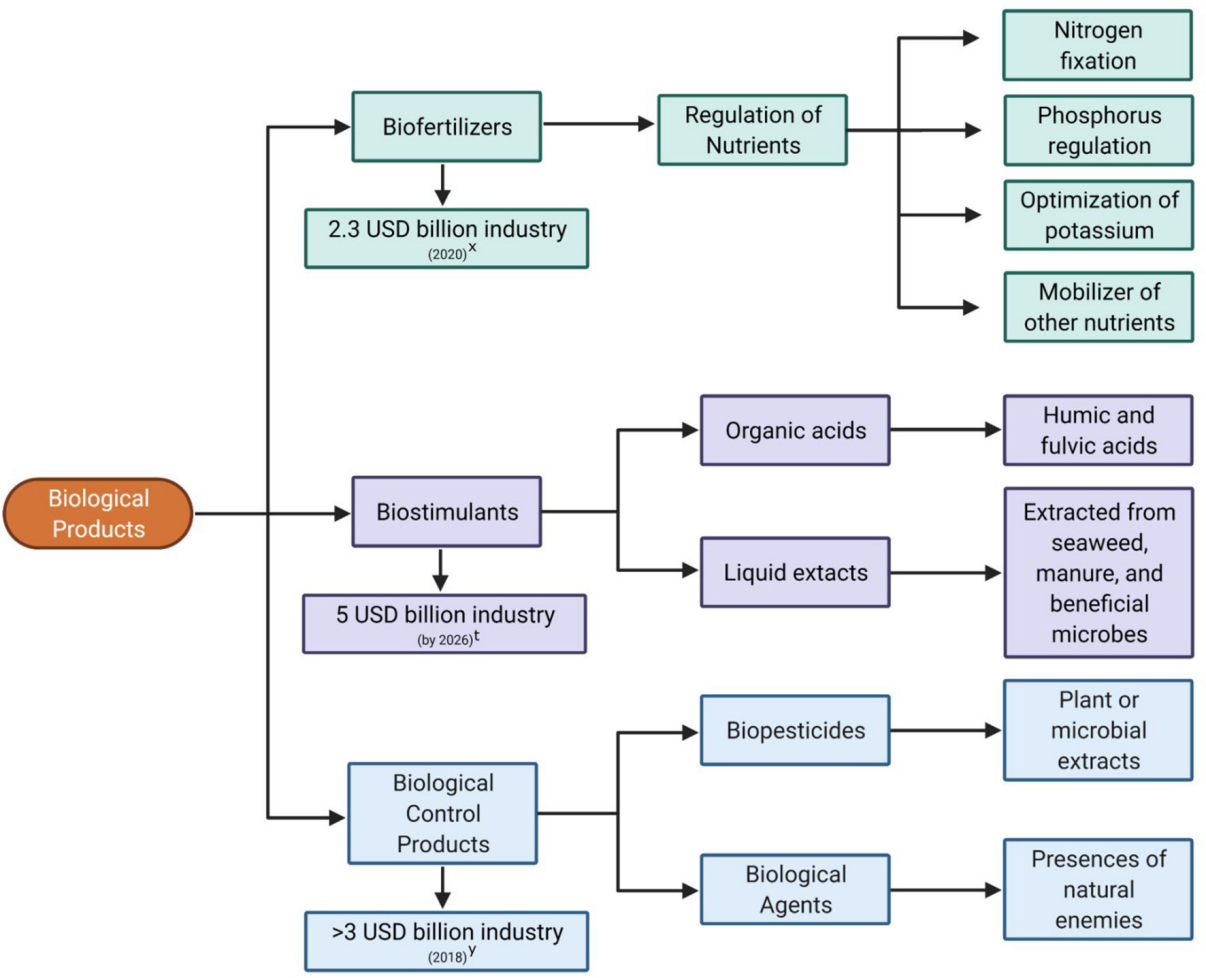
Summary of the various types of biological products and their market value. (x) https://markets.businessinsider.com/news/stocks/biofertilizers-market-worth-3-9-billion-by-2025-exclusive-report-by-marketsandmarkets-1029369497; (t) http://www.globenewswire.com/en/news-release/2020/12/22/2149738/0/en/Biostimulants-Market-revenue-worth-5-billion-by-2026-Says-GMI.html; (y) https://www.gminsights.com/industry-analysis/biocontrol-agents-market.

Biological products including biofertilizers, biostimulants, bioherbicides, and biological control products are a multi-million-dollar industry (Bale et al., 2008; Barratt et al., 2018; van Lenteren et al., 2018) and projected to become a multi-billion-dollar industry in the next few years (Figure 1). Currently, there are few studies investigating the impact that *Trichoderma* or its derived natural products have on the biological control market. Although *Trichoderma* species represent 50–60% of the fungal biological control agents (Whipps, 2001; Verma et al., 2007), their potential market value remains uncertain. Natural product–derived drugs represent 25–50% of currently marketed drugs (Kingston, 2011) and have been the source of new drugs for nearly 40 years (Newman and Cragg, 2020). The development of biological control products was predicted to be costly; however, the rate remains reasonable compared with the cost of the synthesis, toxicological evaluation, and marketing of a new pesticide (Bale et al., 2008). The main caveat is that it can take up to 10 years to complete an extensive efficacy and non-targeted effect studies before releasing a new biological control product (Bale et al., 2008).

Biofertilizers consist of microorganisms that enhance the supply of adequate nutrients to crop plants (Reddy et al., 2020). Biostimulants are any substances/mixtures of natural origin or microorganisms that improve the condition of a crop without causing adverse effects (Rouphael and Colla, 2020). Biological control products comprise both biopesticides and biological control agents. Biopesticides are microbes known to produce antagonistic secreted molecules (i.e., metabolites, peptides, etc.), while biological control agents are microbes used as active ingredients owing to their ability to compete for food or space, their mycoparasitism and antibiosis capacities, or their ability to induce plant defense responses (Contreras-Cornejo et al., 2016). Characterizing the diversity of natural products among microbial protagonists, like *Trichoderma*, will undoubtedly add to the growing market and provide necessary resources required for a sustainable future in agriculture.

### *Trichoderma* As a Ubiquitous Genus Worthy of Exploring for Biocontrol Product Discovery

**Figure 2:**
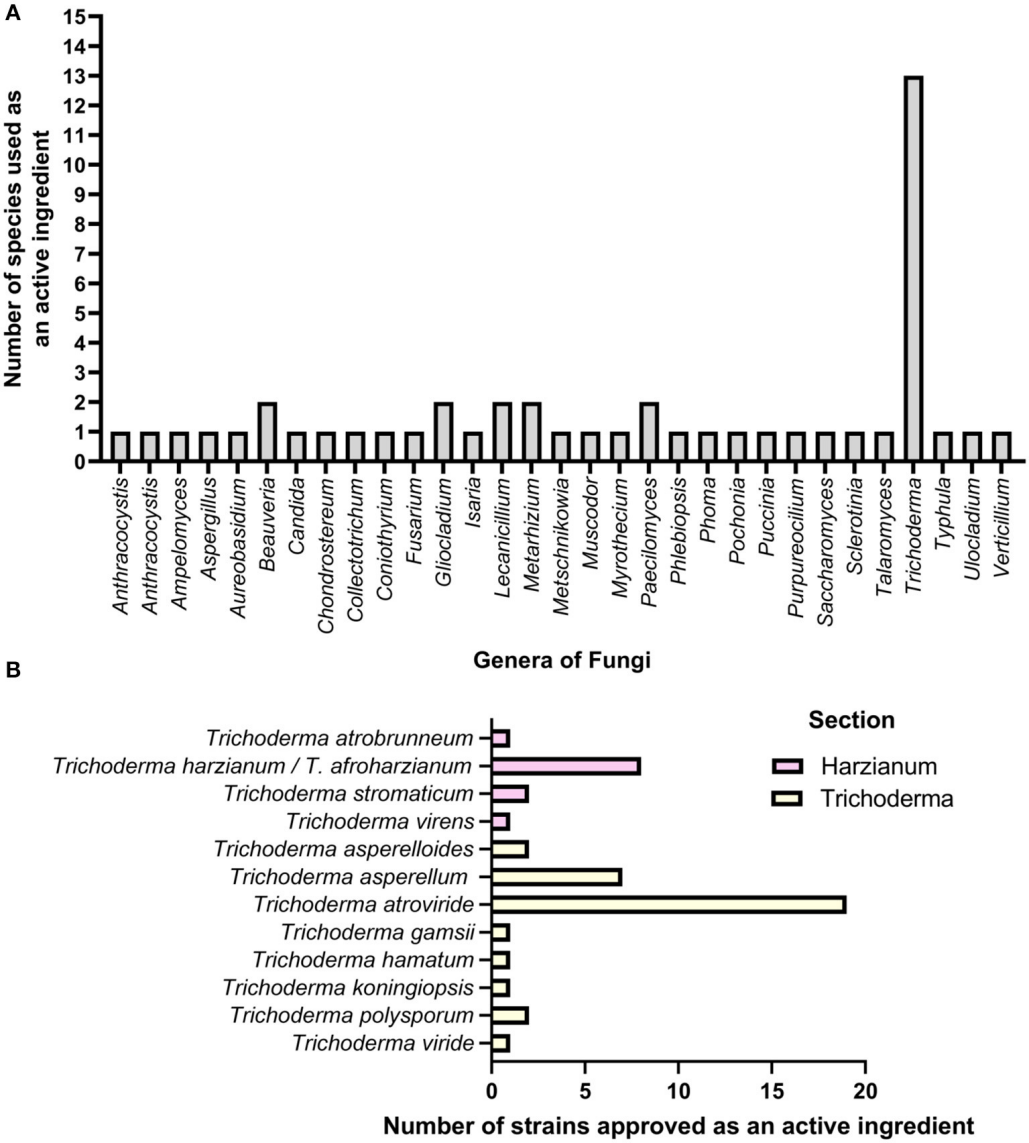
The application of fungi as an active ingredient in commercially available products. **(A)** Fungal genera used as active ingredients in commercially available products. **(B)** The number of *Trichoderma* strains approved as active ingredients in 26 commercially available pesticides produced by five separate companies. *T. harzianum* and *T. virens* were co-active ingredients in four pesticides. *T. asperellum* and *T. gamsii* were co-active ingredients in five pesticides. A combination of multiple strains of *T. atroviride* were used in four pesticides.

*Trichoderma* are asexual, spore-producing, fungicolous ascomycete fungi that are easily isolated, culturable in substrate media, and present in nearly all soils and other diverse habitats (Harman et al., 2004; Harman, 2006; Schmoll and Schuster, 2010; Druzhinina et al., 2011; Kubicek et al., 2019; Sun et al., 2019). *Trichoderma* species are generalists, as they can thrive on resources provided by plants, other fungi, and animals (Kubicek et al., 2019). In some scenarios, many species of *Trichoderma* may act as facultative endophytes (Druzhinina et al., 2011). *Trichoderma* species evolved as versatile biotrophic associates that promote plant health and growth and have shown biocontrol activity against various plant pathogens (Vinale et al., 2008a,b; Lorito et al., 2010). Thus, they have become popular choices as biological control products (Howell, 2003; Benítez et al., 2004; Vinale et al., 2008a,b; Lorito et al., 2010; Kubicek et al., 2011; Kumar and Ashraf, 2017; Mukhopadhyay and Kumar, 2020). Numerous *Trichoderma* species are biological control agents, biofertilizers, and biostimulants, and they produce secondary metabolites with biopesticide activities (Harman et al., 2004; Harman, 2006; Vinale et al., 2008a,b; Lorito et al., 2010; Schmoll and Schuster, 2010; Druzhinina et al., 2011; Mendoza-Mendoza et al., 2018; Kubicek et al., 2019; Thambugala et al., 2020). As of July 2020, there are 375 species with valid names (Cai and Druzhinina, 2021). As of April 2021, there were 453 records of *Trichoderma* species found on Index Fungorum (http://www.indexfungorum.org) and 337 taxonomic species with sequences on the National Center for Biotechnology Information (NCBI), excluding confer (Cf.) names and uncharacterized isolates. However, only a handful of *Trichoderma* species are used for their biocontrol properties, surpassing any other fungal genera used as biological control agents. Currently, 31 fungal genera are used as active ingredients in biological control products, of which 26 belong to the Ascomycota phylum and the rest are basidiomycetes (Figure 2A). *Trichoderma* has by far the highest number of species used as biocontrol agents, a total of 13 (Figure 2B). A similar study showed that ascomycetes have the highest number of bioactivities against plant pathogens, and *Trichoderma* was the most used active ingredient (Thambugala et al., 2020).

Within the species of *Trichoderma*, 30 strains are approved and used in current biocontrol products in Asia (Japan), European Union, North America (U.S.A.), Oceania (Australia and New Zealand), and South America (Brazil and Uruguay), as evidenced by the research conducted for this paper and the results in other literature (Kaewchai et al., 2009; van Lenteren et al., 2018; Supplementary Table 1). There are 14 root applications, 1 root and foliar application, and 2 foliar sprays available with *Trichoderma* species as active ingredients known to have antagonistic effects against 21 soil-borne pathogens and 7 foliar pathogens (Supplementary Table 1). This list includes only product labels that specifically indicate which *Trichoderma* species and strains are used.

The effects of *Trichoderma* species on other organisms are largely influenced by the production and secretion of metabolites, which have various established roles. Fungal metabolites have been reported to act either as communication signaling molecules between microorganisms and their hosts, or as defense agents in interactions with neighboring organisms. They were also shown to influence the development of the producing organism and to stimulate or inhibit the biosynthesis of other metabolites (Keller et al., 2005; Pusztahelyi et al., 2015; Macheleidt et al., 2016; Keller, 2019; Rokas et al., 2020). Genes responsible for the biosynthesis of secondary metabolites are often arranged into clusters (Keller, 2019). Those clusters are regulated by environmental signals and by transcriptional and epigenetics modulators (Keller, 2019). Different classes of secondary metabolites reported in fungi are indole alkaloids, non-ribosomal peptides (NRPs), polyketides, shikimic acid-derived compounds, and terpenoids (Keller et al., 2005; Pusztahelyi et al., 2015; Keller, 2019). Although *Trichoderma* is one of the mass producers of secondary metabolites with 23 identified families, classes, or compounds (Reino et al., 2008), and some with genetic accessibility (Schmoll and Schuster, 2010; Cardoza et al., 2011; Mukherjee et al., 2012; Keswani et al., 2014; Contreras-Cornejo et al., 2016; Zeilinger et al., 2016; Keller, 2019; Li et al., 2019; Vicente et al., 2020), little is known about the biosynthetic gene clusters responsible for the production of those metabolites. Moreover, the level of diversity among secondary metabolites produced across known *Trichoderma* species is still largely indefinite (Kubicek et al., 2019).

## Methods

**Figure 3:**
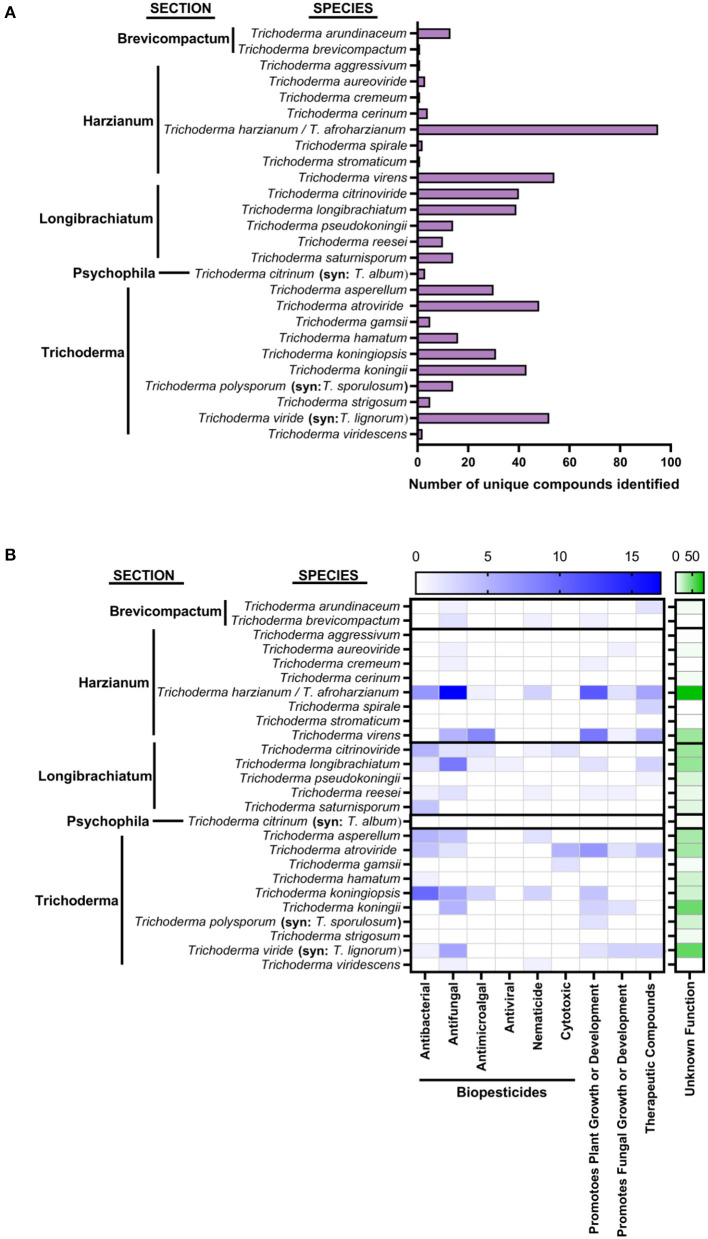
Number of known compounds identified in *Trichoderma* species **(A)** Species of *Trichoderma* organized by section, showing the number of unique compounds identified. **(B)** Their bioactivity refers to metabolites' activities. Metabolites with antibacterial, antifungal, antimicroalgal, antiviral, nematicide, and cytotoxic properties could be used as biopesticides. Metabolites that promote plant growth or development could be used as biofertilizers or biostimulants. Metabolites that promote the fungus' own growth and development have no assigned category. Therapeutic compounds have been identified for their potential use in the medical industry.

To provide a holistic view of metabolites produced by *Trichoderma* species, we summarized data provided by three recent reviews. An estimated 440 different molecules/non-volatile compounds/metabolites were identified and characterized from *Trichoderma* species (Keswani et al., 2014; Contreras-Cornejo et al., 2016; Li et al., 2019). The activities of those 440 projected compounds, reported in reviews by Keswani et al. (2014), Contreras-Cornejo et al. (2016), and Li et al. (2019), are assembled in Figure 3. If a compound has no activity reported, it is categorized as an “unknown function.” Compounds that function as biofertilizers or biostimulants are in the category “promoting plant growth or development.” Compounds with medical implications, such as being anti-tumor, anti-cancer, and so on, are placed in the “therapeutics” category. Other compounds are categorized based on their functions ascribed in previous reports. Species names were validated based on accepted *Trichoderma* names (Zhu and Zhuang, 2015; Li J. et al., 2018; Cai and Druzhinina, 2021) and Index Fungorum's current nomenclature (http://www.indexfungorum.org/) as of April 2021. The taxonomy of *Trichoderma* is cumbersome and there are multiple discrepancies in the nomenclature at the species level, thus leading to incorrect identifications of strains (Cai and Druzhinina, 2021). *Trichoderma* has infrageneric groups that are divided into sections or groups. Sections were assigned based on previous phylogenies data of *Trichoderma* (Druzhinina and Kubicek, 2005; Zhu and Zhuang, 2015; Li J. et al., 2018; Cai and Druzhinina, 2021). Pachybasium is paraphyletic (Druzhinina and Kubicek, 2005), and whether it is a section or group is indistinct. Therefore, all species belonging to Pachybasium “clade A” or “clade B” were placed in “Pachybasium.” Finally, *T. harzianum* strain T22, one of the commonly used active ingredient in commercial products, was determined to be *T. afroharzianum* (Chaverri et al., 2015; Cai and Druzhinina, 2021), the causal agent of *Trichoderma* ear rot on maize (Pfordt et al., 2020). Both species are part of the *T. harzianum* species complex (Chaverri et al., 2015). However, strain T22 still is reported in commercial products as *T. harzianum* or in the literature as *T. harzianum* or *T. afroharzianum*. Without further bioinformatic investigation, it is difficult to know of the reported strains used in commercial product or previous publications (other than strain T22) with biocontrol activity is *T. harzianum* or *T. afroharzianum*, so we used the term “*Trichoderma harzianum*/*T. afroharzianum*” in our analysis for Figures 2, 3 and Supplementary Table 1. However, for the antiSMASH and amPEPpy results coupled with the graph theoretic analysis, we used the genome of *T. harzianum* strain CBS 226.95 which is the neotype (Rifai, 1969; Druzhinina et al., 2018) and *T. afroharzianum* strain BFE349 (Landeis and Schmidt-Heydt, 2021).

## Results

Collectively, 27 described species of *Trichoderma* were reported to produce multiple compounds that were identified and characterized as (1) an enzyme that is part of a pathway to produce a secondary metabolite, (2) a described secondary metabolite, or (3) a compound with an unknown function as shown in Figure 3. Multiple species shared several of these compounds. Of these identified metabolites, several have been shown to have antibacterial or antifungal properties both *in vitro* and *in vivo* (Keswani et al., 2014; Contreras-Cornejo et al., 2016; Li et al., 2019); yet, most of these molecules' specific activity remains unknown (Contreras-Cornejo et al., 2016; Figure 3B). Many of these compounds are intermediates in the biosynthetic pathways of several *Trichoderma* species secondary metabolites that have agricultural and medical applications. Finally, there are biofumigants with antimicrobial and/or plant growth-promoting properties that are volatile organic compounds, as reviewed by Lee et al. (2016), Li N. et al. (2018), and Guo et al. (2019). However, they are not listed in Figure 3.

As shown in Figure 3A, the sections Harzianum, Trichoderma, and Longibrachiatum have the most identified compounds as of 2019. Harzianum has the most described functions for these identified compounds. Most of these compounds fall into the category biopesticides (as stated previously, Figure 3B). However, to our knowledge, none of these compounds have been used as active ingredients in commercial biopesticides.

Not surprisingly, the top five compounds frequently found, as shown in Figure 3, are mostly shared among various *Trichoderma* species (Reino et al., 2008). These are Koninginins, Trichorovins, Trichokonins, 6-Pentyl-2*H*-pyran-2-one, and Trichocaranes. Koninginins have structural similarities to compounds like flavonoids and vitamin E. They can inhibit phospholipase A2 (PLA2) and have been shown to have mycotoxic capabilities (Souza et al., 2008) as well as have antimicrobial properties (Reino et al., 2008). Koninginins have been found in *T. koningii, T. aureoviride, T. harzianum, T. kongingiopsis*, and a brown mutant of *T. viride* exposed to ultraviolet light (Reino et al., 2008). Recently they were identified from *Phomopsis stipata* (Biasetto et al., 2020). Trichorovins is an 11-residue peptaibol, originally described from *T. viride* (Fujita et al., 1994), that forms voltage-dependent and cation-selective ion channels in planar lipid bilayer membranes (Wada et al., 1996). Trichorovins have been found in *Trichoderma longibrachiatum; T. lixii; T. harzianum*, and *T. viride*. Trichokonins were described from *T. koningii* (Huang et al., 1995) and are broad-spectrum antimicrobial peptaibols with bioactivity over a wide pH and temperature range. They have no loss of activity even after autoclaving and are insensitive to proteolytic enzymes (Xiao-Yan et al., 2006). Trichokonins were found in *T. koningii, T. longibrachiatum*, and *T. pseudokoningii*. The category 6-Pentyl-2*H*-pyran-2-one are 2-pyranones, antifungal agents, with phytotoxic activity and antagonistic effects against multiple pathogenic fungi (Reino et al., 2008). This compound gives *Trichoderma* spp. a coconut aroma (Reino et al., 2008). They have been found in *T. atroviride, T. harzianum, T. koningii, T. viride, T. viridescens, T. asperellum*, and peaches (Parker et al., 1997; El-Sayed et al., 2014). Finally, Trichocaranes are metabolites with carotene skeletons that inhibit the growth of etiolated wheat coleoptiles (Macias et al., 2000). They appear to be unique to *T. virens*.

Besides secondary metabolites, antimicrobial peptides (AMPs) are another resource for biological products. AMPs, a cell defense mechanism produced by many organisms, are short and generally positively charged peptides that can directly kill microbial pathogens by modulating the host defense system (Mahlapuu et al., 2016; De Cesare et al., 2020). There has been increased AMP research over the years because of concerns regarding the advent of a “post-antibiotic era” (Mahlapuu et al., 2016). In addition, bacterial resistance to AMPs has been shown to be low or potentially negligible (Spohn et al., 2019). To date, there are more than 3,000 characterized AMPs based on their source, activity, structural characteristics, and amino acid composition (Wang et al., 2016; Huan et al., 2020). Many AMPs interact with membranes, causing cell wall inhibition and nucleic acid binding (De Cesare et al., 2020). Among other types, *Trichoderma* has a unique class of AMPs called peptaibols that include rare amino acids in their sequences, which provide resistance to the host or pathogen proteases and induce programmed cell death in plant fungal pathogens (Montesinos, 2007; Shi et al., 2012; Arinbasarova et al., 2017; Dotson et al., 2018; De Cesare et al., 2020; Sood et al., 2020). While the discovery of AMPs is not new, recent technological and computational advancements are expected to improve their classification, exploration, and characterization (De Cesare et al., 2020; Huan et al., 2020).

### Roadmap

As previously mentioned by Bale et al. (2008), it can be as much as 10 years before a newly discovered biological control agent is released. Therefore, we provide a roadmap to guide researchers with a thorough experimental plan to discover a novel product and implement it into the market. Our roadmap details two starting points, an omics road or biodesign road, to predict and identify putative natural products. Using reference genomes, the omics road queries candidate species for predicted backbone enzymes, putative metabolites, or annotated proteins relevant to biocontrol. Given the dynamic nature of genome expression, computational approaches, like machine-learning or graph theoretical methods, benefit greatly from the addition of functional genomics data (e.g., transcriptomics, proteomics, and metabolomics). In parallel or separately, the challenges of linking predictable gene clusters to their corresponding compounds (Kenshole et al., 2021) is addressed by following the biodesign road to extract putative metabolites, isolate them, and test for bioactivity. Both roads merge at the implementation step, where the metabolite characterized for specific bioactivity can be used as a biological control product. The implementation will determine the compound, its bioactivity, the gene(s) or biosynthetic pathways responsible for its production, and its potential use in a greenhouse or field setting. Collectively, the roadmap provides insightful experimental planning that might allow for faster approval of a novel biological control product into the market (Figure 10).

### Genomic Prediction Tools Highlight Natural Product Diversity Across *Trichoderma* Species

Advancements in high-throughput sequencing technologies have greatly reduced the cost of genotyping organisms, which has expanded genomic libraries for numerous fungi. With increased fungal genome data now available, advances in computing and bioinformatic algorithms are improving our understanding of fungal biology and evolution (Ma and Fedorova, 2010; Aguilar-Pontes et al., 2014; Grigoriev et al., 2014; Stajich, 2017) and new machine learning-based gene prediction tools are beginning to address the problem of gene function discovery (Chavali and Rhee, 2018; Mahood et al., 2020).

**Figure 4:**
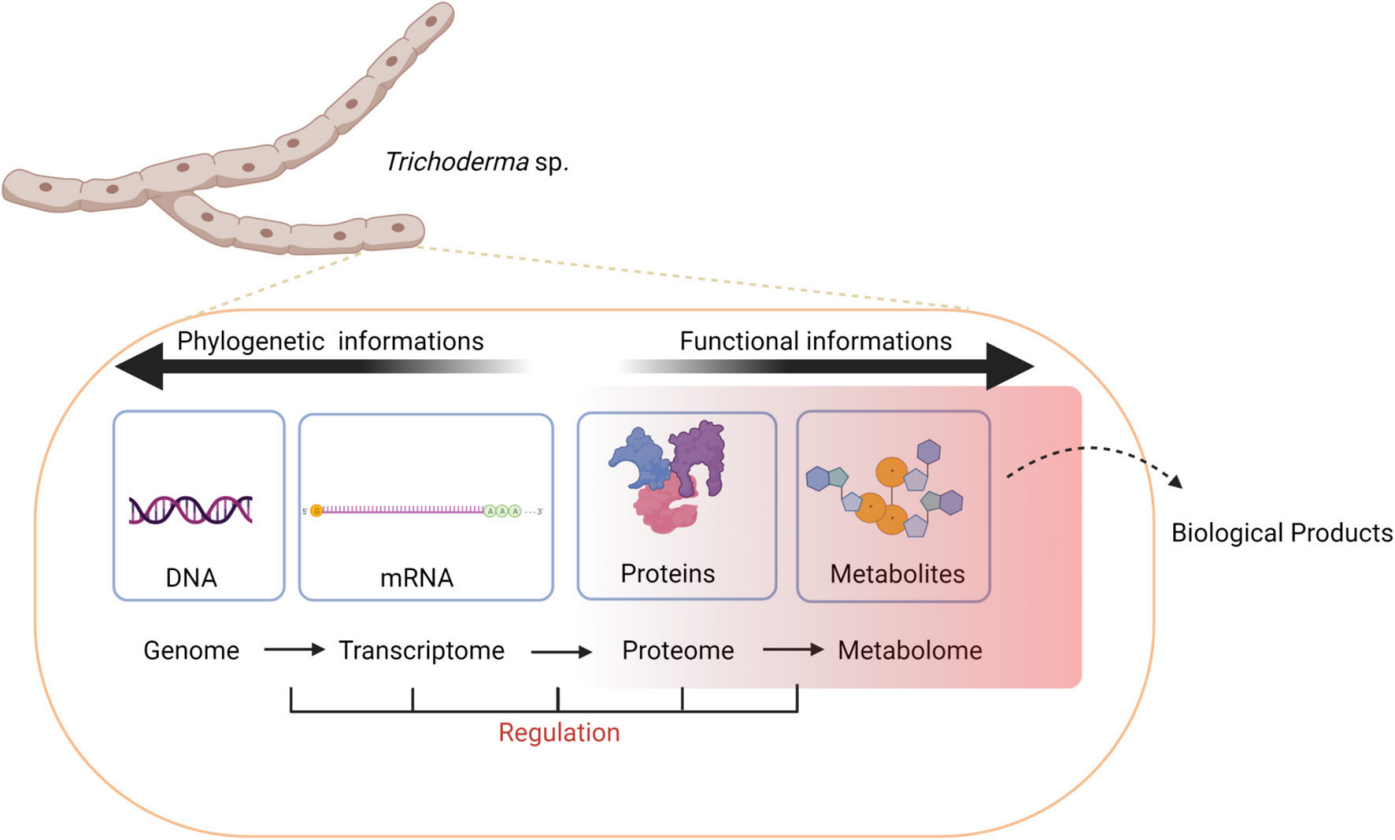
Natural products are the result of layers of regulation transcending the genome, transcriptome, proteome, and metabolome levels that are altered in response to changes in endogenous or environmental cues.

When using genomic prediction tools, it's important to note that several genes, which are often clustered together on the chromosomes, can be required for the biosynthesis of a single compound (Smedsgaard and Nielsen, 2005). Moreover, many gene clusters associated with the production of natural products respond to specific stimuli, such as environmental cues, nutrients, signaling compounds, or other stress factors (Hertweck, 2009) to become active (Gupta et al., 2014; Khan et al., 2020) (Figure 4). Hence, genomic predictions are often complemented with other omics measurements (e.g., transcriptome and proteome) to better understand what biological conditions are required for production of select natural products.

As of April of 2021, there were 25 species of *Trichoderma* available for download from the NCBI database (ncbi.nlm.nih.gov; Table 1). *Trichoderma cyanodichotomus, T. longibrachiatum, T. parareesei*, and *T. reesei* are type specimens; *T. asperellum* and *T. oligosporum* are holotype specimens; and *T. harzianum* is a neotype specimen. It is worth mentioning that the species *T. atrobrunneum* strain ITEM 908 and *T. atroviride* strain IMI 206040 are registered active ingredients in biological products (Supplementary Table 1). The rest of the sequenced strains are characterized species with no assigned type.

**Table 1:** List of *Trichoderma* species examined in this study.

**Species**	**Strain**	**Ecology/importance**	**Strain registered as microbial biological control agent?**	**References (publications or institutions)**	**NCBI BioProject number(s)**
*Trichoderma afroharzianum*	BFE349	Identifying secondary metabolite gene clusters	No	Landeis and Schmidt-Heydt, 2021	PRJNA682927
*Trichoderma arundinaceum*	IBT 40837	Produces terpenoid toxins-Trichothecenes	No	Proctor et al., 2018	PRJNA374046
*Trichoderma asperellum*	CBS 433.97	Biocontrol agent and plant growth promoter	No	Druzhinina et al., 2018	PRJNA453597, PRJNA207877
*Trichoderma atrobrunneum*	ITEM 908	Biocontrol agent and registered to produce commercial biopesticides	Yes	Fanelli et al., 2018	PRJNA428936
*Trichoderma atroviride*	IMI 206040	Mycoparasite of fungal pathogens	Yes	Kubicek et al., 2011	PRJNA264112, PRJNA19867
*Trichoderma brevicompactum*	IBT 40841	Producer of industrial enzymes and biocontrol agents	No	Proctor et al., 2018	PRJNA374046
*Trichoderma citrinoviride*	TUCIM 6016	Reference genome	No	Druzhinina et al., 2018	PRJNA453598
*Trichoderma cyanodichotomus*	TW21990-1	Novel species	No	Zhou et al., 2020	PRJNA598077
*Trichoderma erinaceum*	CRRI-T2N1	Biocontrol agent and plant growth promoter	No	Swain et al., 2018	PRJNA632694
*Trichoderma gamsii*	T6085	Suppression of plant diseases	No	Baroncelli et al., 2016	PRJNA342687
*Trichoderma guizhouense*	NJAU 4742	Mycoparasitism on soil-borne fungi, colonizes roots of plants, shows enhancement of nutrient uptake, promoting plant growth	No	Zhang et al., 2019	PRJNA314460
*Trichoderma hamatum*	GD12	Biocontrol agent and plant growth promoter	No	Studholme et al., 2013	PRJNA178391
*Trichoderma harzianum*	CBS 226.95	Biocontrol of plant pathogens	No	Druzhinina et al., 2018	PRJNA207867
*Trichoderma koningii*	JCM 1883	Cellulase enzyme production	No	Halliwell and Griffin, 1973; RIKEN Center for Life Science Technologies, Division of Genomic Technologies	PRJDB3615
*Trichoderma koningiopsis*	POS7	Enzyme production	No	Castrillo et al., 2017	PRJNA356137
*Trichoderma lentiforme*	CFAM-422	Producer of cellulases and hemicellulases	No	Steindorff et al., unpublished^1^	PRJNA473534
*Trichoderma lixii*	MUT3171	Mycoremediation	No	Venice et al., 2020	PRJNA514353
*Trichoderma longibrachiatum*	ATCC 18648	Biocontrol agent and nematicide activity	No	Xie et al., 2014	PRJNA207876
*Trichoderma oligosporum*	CGMCC 3.17527	Biocontrol agent (especially against white nose syndrome in bats)	No	Wang et al., unpublished^2^	PRJNA644467
*Trichoderma parareesei*	CBS 125925	Ancestor of industrial cellulase and hemicellulase producer *T. reessi*	No	Yang et al., 2015	PRJNA287603
*Trichoderma pleuroti*	TPhu1	Causes green mold disease of cultivated mushrooms	No	Urbán et al., 2016	PRJNA335988
*Trichoderma reesei*	QM6a/ATCC 13631	Used for cellulase production	No	Martinez et al., 2008	PRJNA225530, PRJNA15571
*Trichoderma virens*	IMV 00454	Isolated from Chernobyl Exclusion Zone	No	Singh et al., 2017	PRJNA355122
*Trichoderma virens*	Gv29-8	Mycoparasite	No	Kubicek et al., 2011	PRJNA264113, PRJNA19983
*Trichoderma viride*	Tv-1511	Cellulase enzymes for biological transformations, growth-promoting and biocontrol effects	No	Guo et al., 2020	PRJNA543939
*Cordyceps militaris*	CM01	Pathogen of insects, therapeutic agent	–	Zheng et al., 2011	PRJNA225510, PRJNA41129

1 *Steindorff, A. S., Formighieri, E. F., Midorikawa, G. E. O., Tamietti, M. S., Ramos, E. Z., Silva, A. S., et al. (unpublished). Genome analysis of cellulolytic fungus Trichoderma lentiforme CFAM-422*.

2 *Wang, C., Zeng, Z., and Zhuang, W. (unpublished). Comparative molecular evolution of chitinases in Ascomycota with emphasis on mycoparasitism lifestyle*.

Herein, we used machine-learning tools to examine the diversity of predicted natural products across available *Trichoderma* species' genomes and graph theory to interpret and qualify the results to highlight organisms having intriguing potential for natural product discovery. Currently, there are several tools available to mine genomic data for the presence of biosynthetic pathways associated with the production of natural product, which are often referred to as secondary metabolites (Fedorova et al., 2012; Chavali and Rhee, 2018), and the origin of specialized molecules like antimicrobial peptides (Xu et al., 2021). Using the widely-used antiSMASH (https://antismash.secondarymetabolites.org/#!/start) tool, we analyzed each publicly available *Trichoderma* genome for genome-wide identification, annotation, and analysis of secondary metabolite biosynthetic gene clusters (BGCs) (Blin et al., 2019). Additionally, each genome was interrogated by the software tool amPEPpy, which uses a random forest classifier to predict putative antimicrobial peptides based on protein sequence characteristics often attributed to antimicrobials (e.g., small open reading frames, positively charged, etc.) (Lawrence et al., 2020).

### Secondary Metabolite Exploration Through Genomic Mining and Computational Analysis

**Figure 5:**
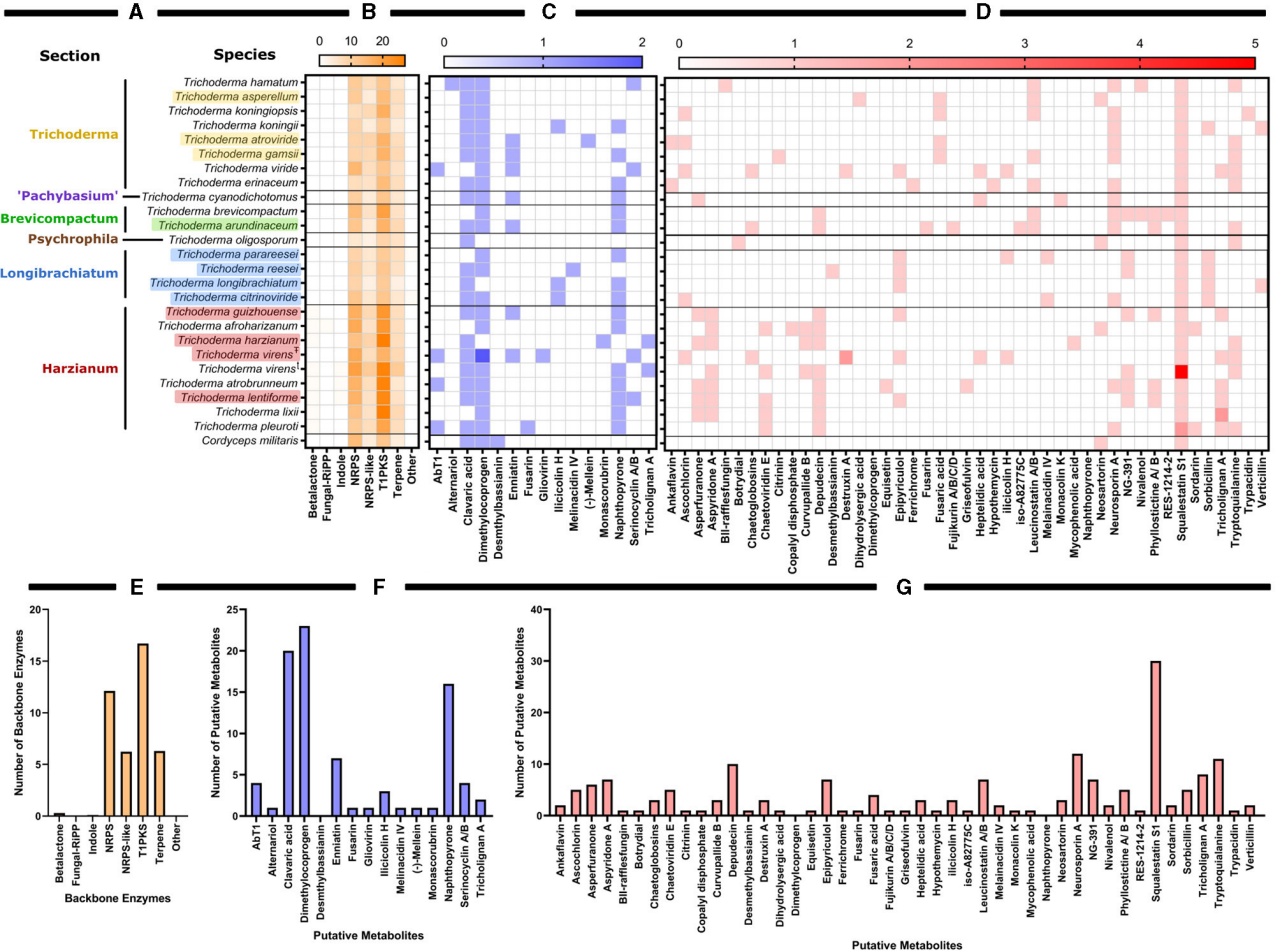
Predictive backbone enzymes an putative metabolites identified by antiSMASH for the available whole genomes of *Trichoderma* species. **(A)** Nucleotide genomes of *Trichoderma* spp. organized by their sections as determined by protein-based genome alignment as shown in Figure 6 and corresponding published data from Druzhinina and Kubicek (2005); Zhu and Zhuang (2015); Li J. et al. (2018), and Cai and Druzhinina (2021). Colored species names correspond with Figure 6. Black text species names correspond with the previous publications on *Trichoderma* taxonomy. Genomes are aligned with corresponding heat map showing **(B)** the number of backbone enzymes and **(C)** putative metabolites with a ≥75% or **(D)** <75% sequence match with known metabolites. **(E)** The total number of backbone enzymes found in all species examined. **(F)** The total number of putative metabolites with a sequence match ≥75% with known metabolites found in all species examined. **(G)** The total number of putative metabolites with a sequence match <75% with known metabolites found in all species examined. Strains of *T. virens* noted as (T) are strain Gv29-8 and as (Ŧ) are strain IMV 00454.

**Figure 6:**
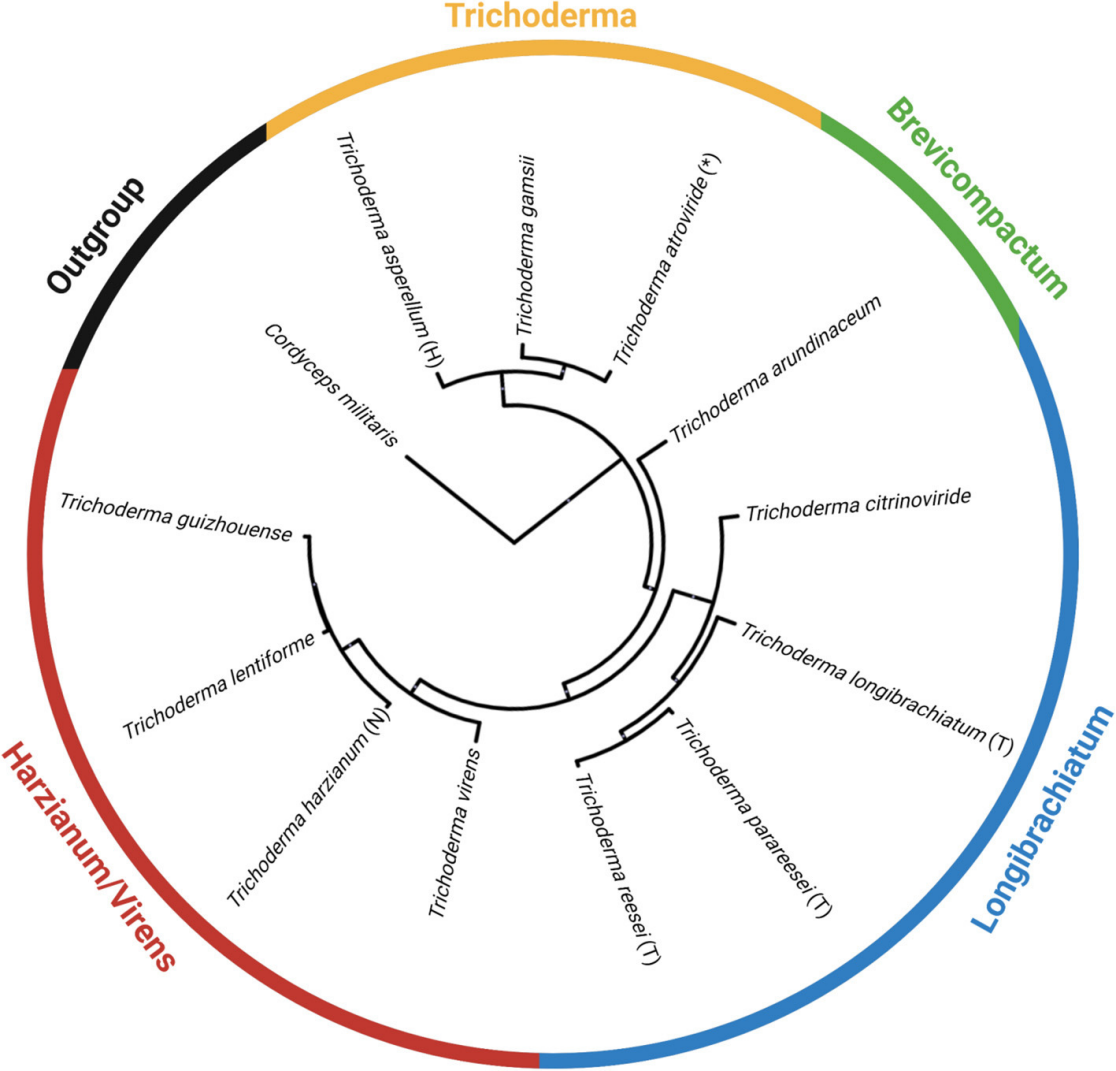
A circular phylogeny of *Trichoderma* spp. based on genomes available on NCBI as of April 2021. The phylogenetic tree is constructed from the identified orthologous protein groups from each *Trichoderma* spp. and one outgroup, *Cordyceps militaris*. The tree was exported and visualized in the interactive Tree of Life—iTOL (https://itol.embl.de/). An asterisk (*) means this strain is used as an active ingredient in commercially available biocontrol products. (T) is the type species; (H) is the holotype; and (N) is the neotype. Data are comparable to the chronogram orthologous protein alignment shown in Kubicek et al. (2019), with *T. arundinaceum*, a Brevicompactum representative as described in Degenkolb et al. (2008a).

Using antiSMASH v5.0, we estimated the total number of backbone enzymes and putative metabolites predicted for each genome, as shown in the heatmap generated in GraphPad Prism v 9.1.0 (221) (Figure 5). The backbone enzyme is the first enzyme in the metabolic pathway that catalyze the synthesis of the core structure, which later undergo several modifications by tailoring enzymes leading to the final product. We have organized the heatmap data (Figure 5) according to the circular phylogeny (Figure 6) produced based on genomes with protein sequences available and based on previous publications for taxonomic resolution within the *Trichoderma* genus for species not included in our phylogenic analysis. However, the taxonomic resolution for each section within *Trichoderma* is an ongoing investigation (Druzhinina and Kubicek, 2005; Cai and Druzhinina, 2021).

Based on *Trichoderma* comparative genomic screening, the Harzianum section has the highest number of backbone enzymes and putative metabolites predicted in those species. However, in commercial biocontrol products, Trichoderma is better represented than Harzianum, despite having fewer predicted backbone enzymes and putative metabolites (as shown in Figure 2B). Type 1 polyketide synthases (T1PKSs) followed by NRP synthetases (NRPSs), were the backbone enzymes most often predicted in *Trichoderma*. NRP is a type of antimicrobial peptide that will be further discussed in this review. This group of metabolites includes peptaibols, which are linear peptides containing between 7 and 20 amino acid residues. In general, polyketides are poorly investigated as biocontrol agents (Daguerre et al., 2017). Specifically, T1PKS have rarely been examined in *Trichoderma* and are proposed to be mostly orthologous groups (Baker et al., 2012). To further investigate these highly anticipated backbone enzymes, machine-learning tools like the DDAP database for screening of biosynthetic T1PKS pathways (https://tylii.github.io/ddap/) can be used. Additionally, NRP discovery and the comprehensive peptaibiotics database can be applied to uncover the identities of these NRPs in *Trichoderma* (Caboche et al., 2008; Stoppacher et al., 2013; Neumann et al., 2015; Flissi et al., 2020). Finally, the data shown in Figure 5 are comparable to those from a previous review in which polyketides, NRPs, and terpenes were largely represented in the genomes of *T. reesei, T. atroviride*, and *T. virens* (Zeilinger et al., 2016).

The top identified putative metabolites with sequence matches ≥75% were dimethyl bassianin (23 matches), clavaric acid (20 matches), napththopyrone (16 matches), enniatin (7 matches), and AbT1 and Serinocyclin A/B with 4 matches each. Dimethyl bassianin (PubChem name is Bassianin) is a pyridine that provides yellow pigment (Lagashetti et al., 2019). Clavaric acid is a triterpenoidal inhibitor with antitumor and antionocogenic activities (Jayasuriya et al., 1998). Napththopyrone is an aromatic polyketide with cytotoxic, antitumor, antimicrobial, and tyrosine kinase properties (Bokesch et al., 2010; Lu et al., 2014; Venice et al., 2020). It has been shown to have a moderate ability to inhibit multidrug transporters and is a pigment that protects fungi from a wide range of predators (Bokesch et al., 2010; Lu et al., 2014; Venice et al., 2020). Enniatin is a mycotoxin discovered in *Fusarium*. Enniatin B was reported to have antifungal activity toward *T. afroharzianum* strain T22 (Meca et al., 2010). AbT1 is a precursor of cyclic peptide antibiotic Aureobasidin A from the crucial industrial yeast, *Aureobasidium pullulans* (Slightom et al., 2009). Serinocyclin A/B is a cyclic heptapeptide reported to cause a sublethal locomotory defect in mosquito larvae (Krasnoff et al., 2007). The top five putative metabolites with sequence matches <75% were squalestatin S1 (syn: zaragozic acid, 30 matches), neurosporin A (12 matches), tryptoquialanine (11 matches), depudecin (10 matches), and tricholignan A (8 matches). Squalestatin S1 controls cholesterol biosynthesis. It targets squalene synthases and has a broad spectrum of antifungal properties (Bonsch et al., 2016; Lebe and Cox, 2019). Neurosporin A is involved in fungal sexual development, provides chemo-resistance to arthropod predation, and demonstrates significant insecticidal activity (Zhao et al., 2017). Tryptoquialanine is an indole alkaloid and a tremorgenic mycotoxin that can elicit intermittent or sustained tremors in vertebrates (Gao et al., 2011). Depudecin is a small linear polyketide known to be a histone deacetylase inhibitor. Moreover, this compound contributes to pathogenesis, has antiprotozoal activity, and provides a more significant fitness benefit (Reynolds et al., 2017). Tricholignan A is a redox-active ortho-hydroquinone that facilitates reductive iron assimilation and plays a potential role in promoting plant growth under iron-deficient conditions (Chen et al., 2019).

A list of the putative metabolites predicted in the genomes of *Trichoderma* species is presented in Table 2. A caveat for associating putative or known metabolites with *Trichoderma* species in Table 2 is that numerous strains have been misidentified at the species level for several reasons: taxonomic identification is missing, non-specific analytical methods were used, species no longer exist in culture, or they do not have DNA sequences available (Nielsen et al., 2005; Degenkolb et al., 2008b; Tijerino et al., 2011; Cai and Druzhinina, 2021). Altogether, this caveat has been problematic for predictive metabolomics in *Trichoderma*. Those putative metabolites, excluding ferrichrome, were described in 31 separate genera, of which 30 belong to the Ascomycota and 1 belongs to the Basidiomycota. Ferrichrome is found in numerous ascomycete and basidiomycete fungi (Haas, 2014). Considering that *Trichoderma* is an ascomycete fungus, one can speculate that these predicted putative metabolites found in other ascomycete fungi could be produced by *Trichoderma* species as well, particularly since they all share a most recent common ancestor (Schoch et al., 2009; Spatafora et al., 2017). Moreover, horizontal gene transfer of secondary metabolite gene clusters has been demonstrated between ascomycete species (Kroken et al., 2003; Patron et al., 2007; Khaldi et al., 2008; Cardoza et al., 2011; Slot and Rokas, 2011; Sieber et al., 2014; Dhillon et al., 2015; Tran et al., 2019; Rokas et al., 2020) and between ascomycete species and other microbes (Schmitt and Lumbsch, 2009; Lawrence et al., 2011). To investigate these putative metabolites in *Trichoderma*, it should be considered that sometimes antiSMASH does not accurately predict gene cluster borders. Therefore, researchers should consider looking at some neighboring genes when performing knockout or overexpression studies to characterize a biosynthetic gene cluster. Besides antiSMASH, there are other computational programs for secondary metabolite gene mining that are less commonly used, e.g., SMURF and ClusterFinder (Tran et al., 2019). Using other available programs for comparison might be useful to obtain more accurate biosynthetic gene cluster (BGC) border predictions. Finally, inconsistency in the nomenclature of metabolites in different biochemical databases used for genome-scale metabolic screening occurs and should be considered in the experimental design (Pham et al., 2019).

**Table 2:** A descriptive list of putative metabolites predicted from antiSMASH based on screening *Trichoderma* genomes.

**Putative metabolite name from antiSMASH result**	**Type**	**Function**	**Characterized in *Trichoderma* species?**	**Fungal genera where metabolite has been characterized**	**References**
AbT1	NRP	Precursor of the cyclic peptide antibiotic, Aureobasidin A	Not characterized in *Trichoderma*	*Aureobasidium*	Slightom et al., 2009; Wang et al., 2020
Alternariol	Terpene	Antifungal and phytotoxin	Found in *Trichoderma* sp. strain Jing-8, showed to be DPPH-radical-scavenging and growth inhibitory Antibiotic	*Alternaria*	Davis and Stack, 1994; Zhang et al., 2017; Li et al., 2019
Ankaflavin	Polyketide: Iterative Type 1	Yellow pigment, anti-inflammation, anti-oxidation, anti-diabetes, immunomodulation and antitumor agent	Not characterized in *Trichoderma*	*Monascus*	Cheng M. J. et al., 2010
Ascochlorin	Terpene + Polyketide	Antibiotic, inhibitor of mitochondrial cytochrome bc1 complex	Not characterized in *Trichoderma*	*Ascochyta*	Berry et al., 2010
Asperfuranone	Polyketide	Antitumor agent	Not characterized in *Trichoderma*	*Aspergillus*	Wang et al., 2010
Aspyridone A	NRP + Polyketide: Iterative type I	Antifungal	Similar in structure to Harzianopyridone, isolated from *T. harzianum*	*Aspergillus*	Wasil et al., 2013; Shenouda and Cox, 2021
BII-rafflesfungin	NRP	Antifungal	Not characterized in *Trichoderma*	*Phoma*	Sinha et al., 2019
Botrydial	Terpene	Antifungal and phytotoxin	Not characterized in *Trichoderma*, but it regulates expression of trichothecene biosynthesis in *T. arundianceum*	*Botrytis*	Malmierca et al., 2016
Chaetoglobosins	NRP + Polyketide: Iterative type I	Antitumor agent	Not characterized in *Trichoderma* but would not be surprising to be present in *Trichoderma* given the large number of polyketide present. Chaetoglobosins and Chaetoviridin biosynthesis is recently characterized in *Chaetomium globosum*	*Chaetomium*	Daguerre et al., 2017
Chaetoviridin E	Polyketide	Antibiotic and cytotoxic	Not characterized in *Trichoderma* but would not be surprising to be present in *Trichoderma* given the large number of polyketide present. Chaetoglobosins and Chaetoviridin biosynthesis is recently characterized in *Chaetomium globosum*	*Chaetomium*	Daguerre et al., 2017
Citrinin	Polyketide: Iterative Type 1	Antibiotic; mycotoxin	Not well-characterized in *Trichoderma*. *Trichoderma hamatum* has been shown to reduce the amount of citrinin produced by other fungus	*Aspergillus, Monascus, Penicillium*	Abd-Allah and Ezzat, 2005; Doughari, 2015
Clavaric acid	Terpene	Farnesyltransferase inhibitor	Not characterized in *Trichoderma*	*Hypholoma*	Jayasuriya et al., 1998
Copalyl disphosphate	Terpene	Tanshinone biosynthesis	Previous study predicted *T. afroharzianum* genome to contain BGC necessary for biosynthesis of this terpene molecule	*Phomopsis*	Toyomasu et al., 2007; Landeis and Schmidt-Heydt, 2021
Curvupallide B	NRP + Polyketide	Phytotoxin	Previously predicted to encoded by *T. afroharzianum*	*Curvularia*	Abraham et al., 1995; Landeis and Schmidt-Heydt, 2021
Depudecin	Polyketide: Iterative Type 1	Inhibitor of histone deacetylase, has anti-angiogenic activity	Not characterized in *Trichoderma*	*Alternaria*	Matsumoto et al., 1992
Desmethylbassianin	NRP + Polyketide: Iterative type I	Closely related to tenellin which are cytotoxic	Not characterized in *Trichoderma*	*Beauveria*	McInnes et al., 1974
Destruxin	NRP	Insecticide and antitumor agent	Not characterized in *Trichoderma*	*Metarhizium, Alternaria, Trichothecium, Aschersonia*	Pedras et al., 2002; Golo et al., 2014
Dihydrolysergic acid	Ergot Alkaloid	Vasorelaxant	Not characterized in *Trichoderma*	*Claviceps*	Arnold and Panaccione, 2017
Dimethylcoprogen (=N alpha-dimethylocoprogens)	NRP	Phytotoxin	Not characterized in *Trichoderma*	*Alternaria*	Jalal et al., 1988
Enniatin	NRP	Antibiotic, antihelmintic, antifungal, herbicidal, and insecticidal compound	Not characterized in Trichoderma, could be Enniatins-like genes	*Fusarium*	Prosperini et al., 2017
Epipyriculol	Polyketide	Phytotoxin	Not characterized in *Trichoderma*	*Pyricularia*	Masi et al., 2017
Equisetin	NRP + Polyketide	Antibiotic, cyctotoxic, has inhibitory effect on HIV-1 integrase	Trichosetin (an Equisetin homolog) isolated in a co-culture with *T. harzianum* and *Catharanthus roseus*	*Fusarium*	Singh et al., 1998; Marfori et al., 2002
Ferrichrome	Other	Siderophore	Found in *T. virens*	Majority of fungi can produce	Haas, 2014; Mukherjee et al., 2018
Fujikurin A/B/C/D	Polyketide	Need more characterization	Not characterized in *Trichoderma*	*Fusarium*	Von Bargen et al., 2015
Fusaric acid	Polyketide	Antibiotic	Not characterized in *Trichoderma*	*Fusarium*	Yabuta et al., 1939
Fusarin	NRP + Polyketide	Mycotoxin	Not characterized in *Trichoderma*	*Fusarium*	Niehaus et al., 2013
Gliovirin	NRP	Antibiotic	An antibiotic found in *T. virens* (isolated from the Gv strains) and *T. longibrachiatum*	*Trichoderma virens* (syn: *Gliocladium virens*)^T^	Stipanovic and Howell, 1982; Keswani et al., 2014; Contreras-Cornejo et al., 2016
Griseofulvin	Polyketide: Iterative Type 1	Antifungal	Not characterized in *Trichoderma*	*Penicillium*	Huber, 1967
Heptelidic acid (syn: Koningic acid)	Terpene	Antibiotic and antitumor agent	Antimicrobial found in *T. virens* and *T. viride*	*Chaetomium* and *Trichoderma virens* (syn: *Gliocladium virens*)^T^	Itoh et al., 1980
Hypothemycin	Polyketide	Antifungal and antitumor agent	Not characterized in *Trichoderma*	*Hypomyces*	Wee et al., 2006
Ilicicolin H	NRP + Polyketide	Antimicrobial	Not characterized in *Trichoderma*	*Clonostachys rosea* (syn: *Gliocladium roseum*)^l^	Schroers et al., 1999; Singh et al., 2012;
ISO-A82775C	Other	Cytotoxicity and antitumor	Not characterized in *Trichoderma*	*Pestalotiopsis*	Suzuki et al., 2017
Leucinostatin A/B	Polyketide	Antibiotic and anti-trypanosomal activity	Not characterized in *Trichoderma*	*Paecilomyces*	Cerrini et al., 1989
Melinacidin IV	Alkaloid	Antimicrobial	Not characterized in *Trichoderma*	*Westerdykella*	Ebead et al., 2012
(-)-Mellein	Polyketide	Neurotoxic effects	Antifungal found in *T. aggressivum*	*Aspergillus*	Moore et al., 1972; Krupke et al., 2003
Monacolin K	Polyketide	Chemical structure similar to lovastatin, a key enzyme in cholesterol lowering drugs	Not characterized in *Trichoderma*	*Aspergillus, Monascus*	Endo et al., 1986
Monascorubrin	Polyketide	Pigment	Not characterized in *Trichoderma*	*Monascus* and *Penicillium*	Woo et al., 2014
Mycophenolic acid	Terpene + Polyketide: Iterative Type I	Antibiotics; immunosuppressant medication	Not characterized in *Trichoderma*	*Penicillium*	Allison et al., 1993
Naphthopyrone	Polyketide	Non-toxic pigment, used for fungal defense; antimicrobial activity; protection against predation	Pigments found in ascomycete fungi, displays cytotoxic activity, predicted to be in *T. lixii*	*Aspergillus, Fusarium, Penicillium*	Xu et al., 2019; Venice et al., 2020
Neosartorin	Polyketide	Yellow pigment	Not characterized in *Trichoderma*	*Neosartorya*	Proksa et al., 1998
Neurosporin A	Polyketide	Insecticide; provide resistance to arthropod predation	Not characterized in *Trichoderma*	*Neurospora*	Zhao et al., 2017
NG-391	NRP + Polyketide	Mycotoxin	Not characterized in *Trichoderma*	*Metarhizium*	Donzelli et al., 2010
Nivalenol	Terpene	Mycotoxin	Nivalenol are trichothecenes and *Trichoderma* produces trichothecenes, mostly in the Brevicompactum section	*Fusarium*	Yamashita et al., 1995; Nielsen et al., 2005; Tijerino et al., 2011; Gutiérrez et al., 2020
Phyllostictine A/B	NRP + Polyketide	Phytotoxin, used as biocontrol herbicide	Not characterized in *Trichoderma*	*Phyllosticta*	Evidente et al., 2008
RES-1214-2	Polyketide	Endothelin antagonists	Not characterized in *Trichoderma*	*Pestalotiopsis*	Ogawa et al., 1995
Serinocyclins A/B	NRP	Sublethal toxic effect	Not characterized in *Trichoderma*	*Metarhizium*	Krasnoff et al., 2007
Sorbicillin	Polyketide	Cytotoxic, antioxidant, antiviral, and antimicrobial activity.	Found in *T. longibrachiatum* and *T. reesei*	*Clonostachys, Emericella, Penicillum, Trichoderma, Verticillium*	Keswani et al., 2014; Meng et al., 2016; Derntl et al., 2017
Sordarin	Polyketide	Antifungal	Predicted to be in *T. harizanum* because it has similar genes clusters, *sdnA* and *sdnO*, in genome	*Sordaria*	Hauser and Sigg, 1971; Baroncelli et al., 2016; Kudo et al., 2016
Squalestatin S1 (syn: Zaragozic acid)	Terpene	Antifungal	Not characterized in *Trichoderma*	*Sporormiella* and *Leptodontium*	Bergstrom et al., 1993; Bills et al., 1994
Tricholignan A	Polyketide: Iterative Type 1	Facilitate reductive iron assimilation in plants	Found in *T. harizanum* to facilitate reductive iron assimilation in plants	*Trichoderma*	Chen et al., 2019
Trypacidin	Polyketide	Cytotoxic, antibiotic	Not characterized in *Trichoderma*	*Aspergillus*	Gauthier et al., 2012
Tryptoquialanine	NRP	Phytotoxin, insecticide, antifungal	Genes responsible for the biosynthesis of Tryptoquialanine, homologs were found in the genome of *T. reesei*	*Penicillium*	Ariza et al., 2002; Gao et al., 2011; Costa et al., 2019a,b; Costa et al., 2021;
Verticillin	NRP	Cytotoxic and bacteriostatic activities	Not characterized in *Trichoderma*	*Clonostachys rosea* (syn: *Gliocladium roseum*)^l^, Paecilomyces, Verticillium^*^	Schenke et al., 2011; Kim and Movassaghi, 2015

*The backbone enzyme type, the function of the predicted metabolite, whether it has been identified in Trichoderma species, and which fungi produce these metabolites are described. Collectively, 31 genera were identified to produce these metabolites (excluding Trichoderma) where 30 genera belong to Ascomycota and one genus belong to Basidiomycota*.

T *Gliovirin and Heptelidic acid were initially described in Gliocladium virens which were transferred to Trichoderma*.

l *Ilicicolin H and potentially Verticillin A were initially described in Gliocladium roseum which was transferred to Clonostachys rosea*.

* *Verticillin A was initially described in Verticillium, however, the genes required to produce this metabolite are not found in Verticillium species (Schenke et al., 2011). Not characterized means the metabolite name has not been identified in Trichoderma*.

**Figure 7:**
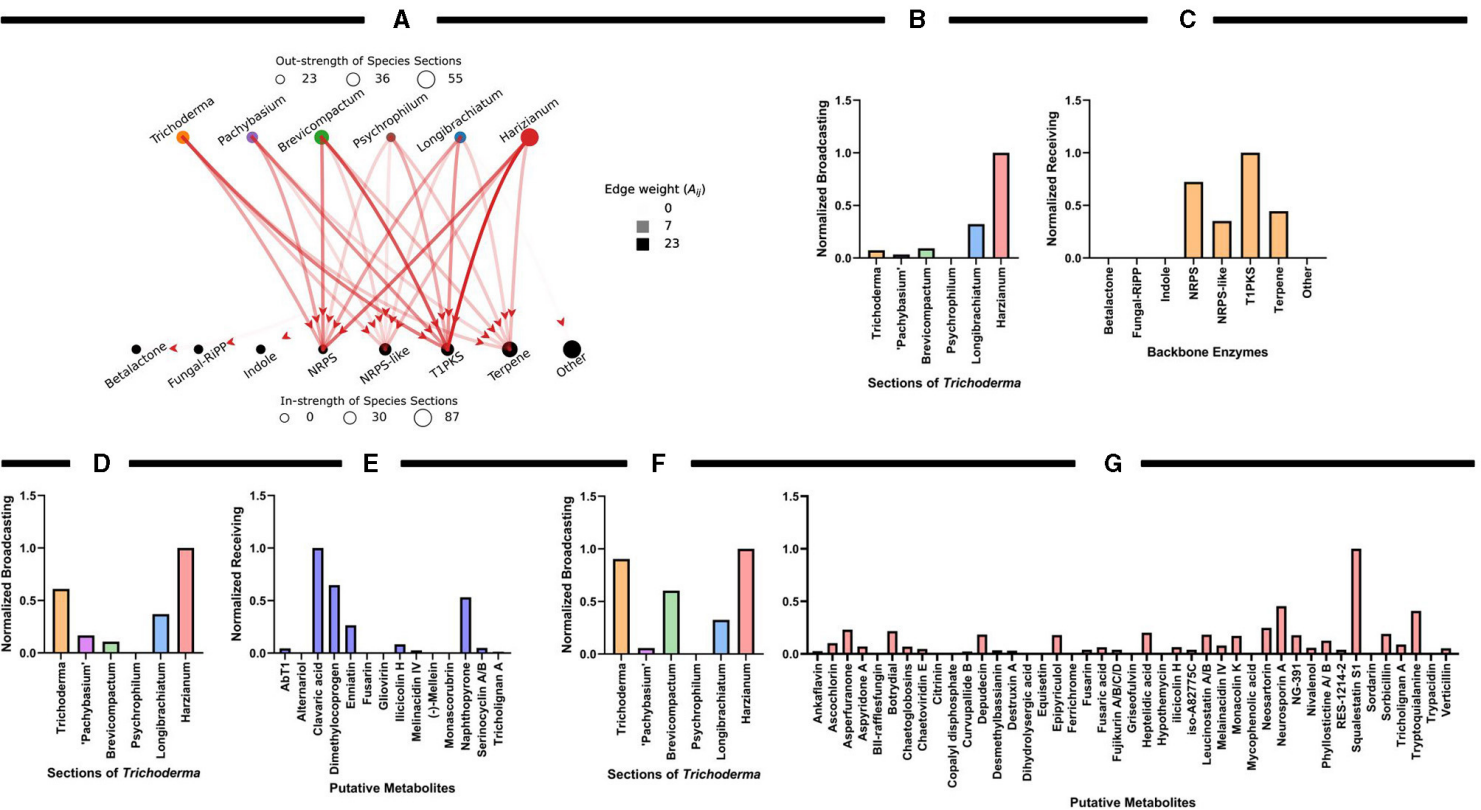
Graph theoretical analysis to characterize influential species sections of *Trichoderma* for identifying putative compounds or metabolites. **(A)** The weighted-directed graph representation of interactions among the sections and backbone enzymes. The edge transparency represents the average number of times an enzyme is identified by a section and the node size (circle radius) represents the total interaction strength of the sections and enzymes. **(B,C)** PageRank centrality measures for the sections and backbone enzymes, quantified by the min-max normalized (values between 0 and 1) broadcasting and receiving values, respectively. **(D,E)** Normalized broadcasting and receiving PageRank measures computed using the interactions among the sections and putative metabolites with a ≥75% match sequence; **(F,G)** represents the same for <75% matched sequences with known metabolites.

To quantify the uniqueness and predictive diversity of natural products across the *Trichoderma* sections or functional categories of secondary metabolites, we used graph theory as a robust mathematical platform to represent the relationships between or influence of antiSMASH results across the different *Trichoderma* sections. In graph theory analysis, a graph is comprised of nodes and edges (Newman, 2018) and, in this context, the sections and backbone enzymes represent the nodes, and the edges are weighted by the average number of times a category is observed by all the species in a section (Figure 7A). We used two different graph-theoretic measures to quantify and rank the importance of sections and backbone enzymes. These are (1) the strength and (2) PageRank of the nodes. The strength of the node is determined by the summation of all the edges from (out-strength) or to (in-strength) a node, representing the same values reported previously in Figure 5E. The PageRank measure (Page and Brin, 1998), which is the underlying method used by Google search engine to rank web pages based on the links between web pages, quantifies the relative importance of a node (section or backbone enzyme) based on the connections it has. We rank the sections and the enzymes for being the most influential and influenced nodes, respectively, using the directed PageRank measures broadcasting and receiving measures (Grindrod et al., 2011) as shown in Figures 7B,C. These values are min-max normalized (between 0 and 1). The above graph-theoretic analysis was performed separately for the interaction among the sections and putative metabolites with ≥75% and <75% matched sequences with known metabolites. We only report the min-max normalized broadcasting and receiving PageRank values for these analyses, as shown in Figures 7D–G.

Overall, the graph theory analysis highlighted notable interactions between organisms and secondary metabolite predictions that can assist with selecting organisms or natural products for further analyses. Interestingly, the Harzianum section has the most prevalence of predicted backbone enzymes and putative metabolites compared to all the other sections examined. This data supports what has been observed in several publications in identifying bioactive compounds (Figure 3) and why several biological products use species from this section are used as active ingredients (Figure 2B). The Trichoderma section showed a higher prediction of putative metabolites found in their genomes (Figures 7D,F), which may explain why species from the Trichoderma section have the most used active ingredient in commercial agricultural products (Figure 2B). While the Longibrachiatum section was shown to have a relatively high number of backbone enzymes found in their genomes (Figure 7B), species from Longibrachiatum are not listed as active ingredients in commercial agricultural labels (Figure 2B). It is interesting, though, that three well-studied species, *T. reesei, T. parareesei*, and *T. longibrachiatum* (Supplementary Table 1) are used in other industries. For example, *T. reesei* strain DSM 32338 was genetically modified to produce muramidase enzyme used as an additive for chickens and other minor poultry for fattening (Rychen et al., 2018). *Trichoderma reesei* is often genetically manipulated to overexpressed genes responsible for cellulases that are then used in the biotechnology industries (Druzhinina and Kubicek, 2017; Hinterdobler et al., 2021). *Trichoderma reesei* has been reported to sexually reproduce which can provide tools for fast and improved development of strains for industrial use (Seidl et al., 2009). However, there can be a pitfall in releasing these species in the environment as an active ingredient because it has the potential to sexually recombine with a native population. But alternatives like the sympatric species, *T. longibrachiatum*, and *T. parareesei* have a clonal lifestyle (Druzhinina et al., 2008, 2010; Atanasova et al., 2010), therefore could be suitable species to use as biological control products. *Trichoderma parareesei* have been shown to produce cellulases like, *T. reesei*, and have biostimulant activities by increasing seedling lateral root development on tomatoes and produced antagonistic effects against fungal foliar pathogen, *Botrytis cinerea* (Rubio et al., 2014). Several publications show the effects of *T. longibrachiatum* as a fungicide and nematicide on several crops, yet the efficacy is unknown (Migheli et al., 1998; Rojo et al., 2007; Zhang et al., 2015, 2017, 2018b). Our data suggest that by using genome-mining companioned with computational analysis, there is evidence of several known *Trichoderma* species that have not been fully explored for their use as a biological product in agriculture. This untapped resource could be part of a solution to overcoming pathogen resistance or other issues growers face today, mainly if the environmental conditions and origins of species isolation are known.

#### Antimicrobial Peptides Exploration Through Genomic Mining and Computational Analysis

To date, there were more than 30 computational methods for AMP prediction and identification as of 2021 (Xu et al., 2021). Of those computational methods, amPEPpy is a user-friendly, open-source, portable, multi-threaded command-line application for predicting AMPs through genome-based screening using a random forest classifier (Lawrence et al., 2020). amPEPpy was validated as predicting AMPs more accurately than other approaches (Xu et al., 2021). We used amPEPpy to predict promising antimicrobial agents (Lawrence et al., 2020) within the *Trichoderma* genomes. We compared predicted AMPs sequences to sequences available on NCBI as of April 2021 because there are no online tools that cross-link data generated from amPEPpy to numerous known AMPs, as antiSMASH does with secondary metabolites. Accession numbers generated by amPEPpy are found in Supplementary Table 2 and functional annotations were summarized by major keywords into categories. Each functional annotation categories represent the percentage of amino acid sequences that match the predicted AMP motif: 100, 99.9–95.0, 94.9–90.0, 89.9–80.0, 79.9–70, and 69.9–51.0%. Anything below 50.9% was not considered for further analysis. A heatmap was generated to reflect these results in GraphPad Prism v 9.1.0 (221), indicating the number of times each category was predicted.

Based on *Trichoderma* comparative genomic screening, the Harzianum section has the highest number of predicted AMPs. Interestingly, among all organisms a large percentage of AMPs were annotated as hypothetical proteins, and the large number of observed hypothetical proteins in *T. harzianum, T. virens, T. reesei, T. atroviride*, and *T. asperellum* is intriguing. In some instances, these hypothetical proteins have a high sequence homology between species. In fact, *T*. *lentiforme* (accession number KAF3073950) and *T. arundinaceum* (accession number RFU77176) were predicted to have a 100% match for an unknown AMP and currently are described as “hypothetical protein” on NCBI.

**Figure 8:**
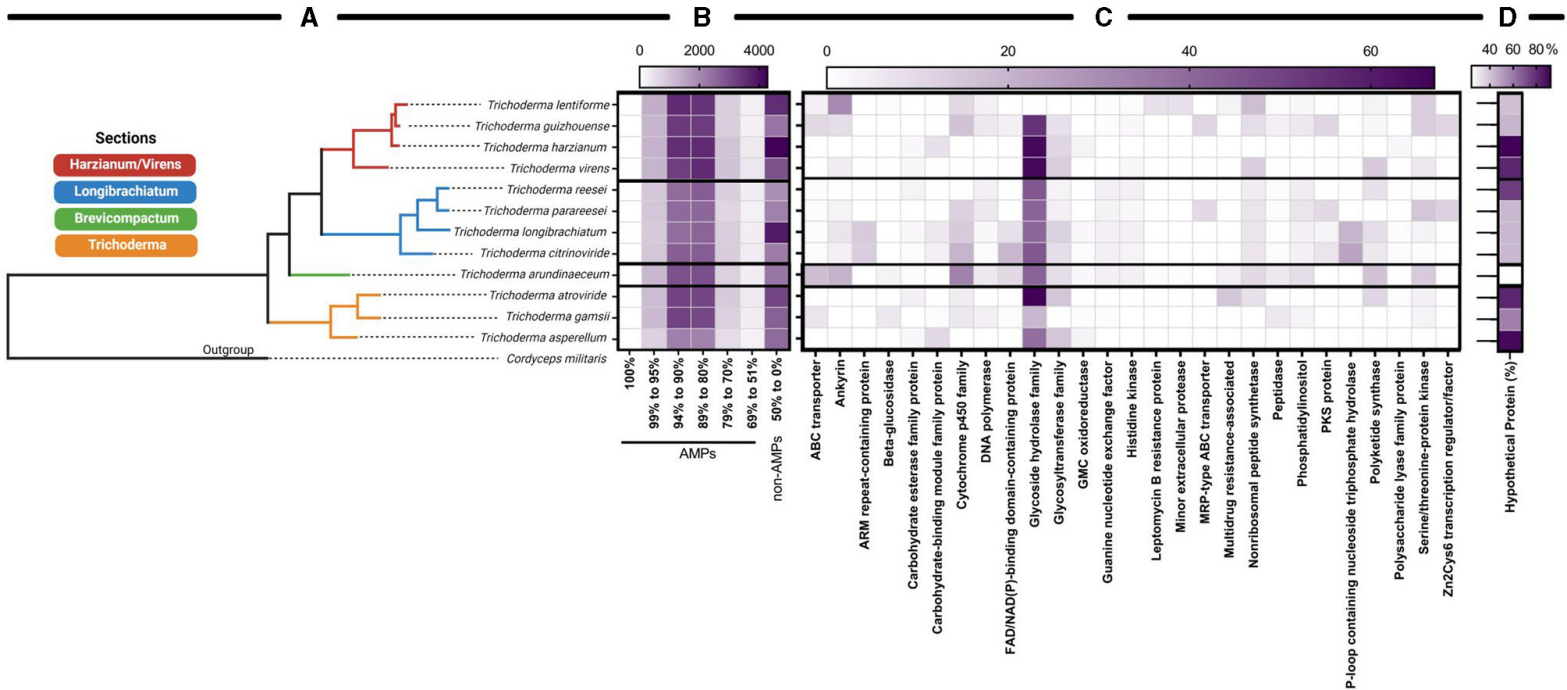
Predictive AMPs found in available genomes of species in *Trichoderma*. **(A)** A dendrogram based on the genomes of *Trichoderma* spp. with reference protein sequences and **(B)** organized by their sections and the corresponding heat map showing the numbers having a 100, 99–95, 94–90, 89–80, 79–70, 69–51, and ≤ 50% match with AMP identifying characters shared by species of *Trichoderma* within their sections. **(C)** The top five annotated accession numbers found across all species from 95 to 100% match of AMPs. **(D)** Percentage of hypothetical proteins from 95 to 100% match of AMPs. The outgroup is *Cordyceps militaris*.

Although hypothetical proteins dominated the annotations in the NCBI accession keyword search (Figure 8D), we listed the top five annotated proteins from each species. Among these, proteins annotated as non-ribosomal peptide synthetases (NRPSs) is a notable annotation because these proteins are known to produce AMPs in bacteria and fungi (Finking and Marahiel, 2004; Felnagle et al., 2008; De Cesare et al., 2020). Apart from this annotation, it was interesting to observed “glycoside hydrolase family” and “cytochrome P450 family” annotations as abundant keyword annotated protein names (Figure 8C). Interestingly, glycoside hydrolase family are found in many microbes and plants and are frequently associated with microbe-host interactions (Faure, 2002; Xu et al., 2004; Berlemont and Martiny, 2016; Snarr et al., 2017; Mendoza-Mendoza et al., 2018). Additionally, they have been shown to be required for needed for virulence in oomycetes and fungi triggering pathogen-associated molecular patterns (Mendoza-Mendoza et al., 2018; Tan et al., 2020; Zhang et al., 2021), formation of biofilms in bacteria (Szymańska et al., 2020), as a therapeutic for antibiofilm activity from pathogens across taxonomic kingdoms (Snarr et al., 2017), and in chemical defenses against herbivory (Xu et al., 2004). Glycoside hydrolases have also been shown to be involved in the production of secondary metabolites and peptaibols that have been found in 21 genomes of *Trichoderma* spp. (Fanelli et al., 2018; Mendoza-Mendoza et al., 2018). In a study to target activity-specific genetic markers for biocontrol from genomes of *Trichoderma* spp., Fanelli et al. (2018) compared protein family (Pfam) database, domains associated with stress tolerance and antagonistic activities, and found that 7 out of 15 Pfam domains with specific roles were CAZyme, containing hundreds of CAZymes proteins (Mistry et al., 2021). Two other Pfam domains with specific roles were the ABC transporter region and peptidase, which were also found in the results of this study (Fanelli et al., 2018). Like the glycoside hydrolases, cytochrome P450 superfamily have also been associated with antimicrobials (Geisler et al., 2013) and the production of primary secondary metabolites (Shin et al., 2018).

**Figure 9:**
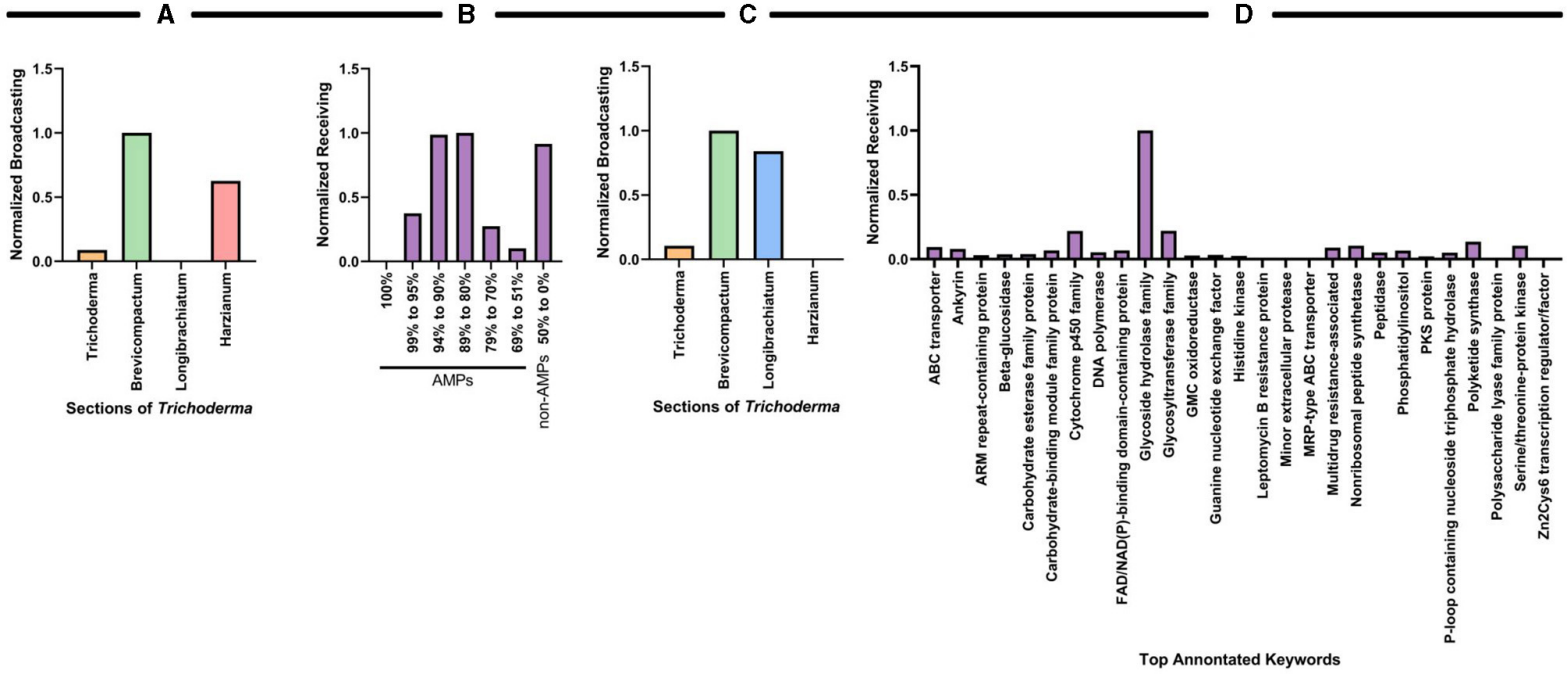
Graph theoretical analysis to characterize influential species sections of *Trichoderma* based on their probability of having AMP-related genes and keyword protein annotations. The PageRank measure for the sections with respect to the probable AMP-related gene are quantified by the normalized **(A)** broadcasting and **(B)** receiving values, respectively. The PageRank measure for the sections with respect to the keyword proteins are quantified by the normalized **(C)** broadcasting and **(D)** receiving values, respectively.

Like the antiSMASH analysis, we used graph-theoretic analysis to further interrogate the predictions from amPEPpy to quantify the importance (or ranking) of the sections with respect to their probability of having AMP-related genes or keyword protein annotations. Two separate analyses were performed using the average values for the species of each section from the data in Figures 8A,B. We measured the min-max normalized broadcasting PageRank of the sections and the receiving PageRank of their probability of having AMP-related genes (Figures 9A,B) and, correspondingly, the measures related to the keyword protein annotations (Figures 9C,D).

**Figure 10:**
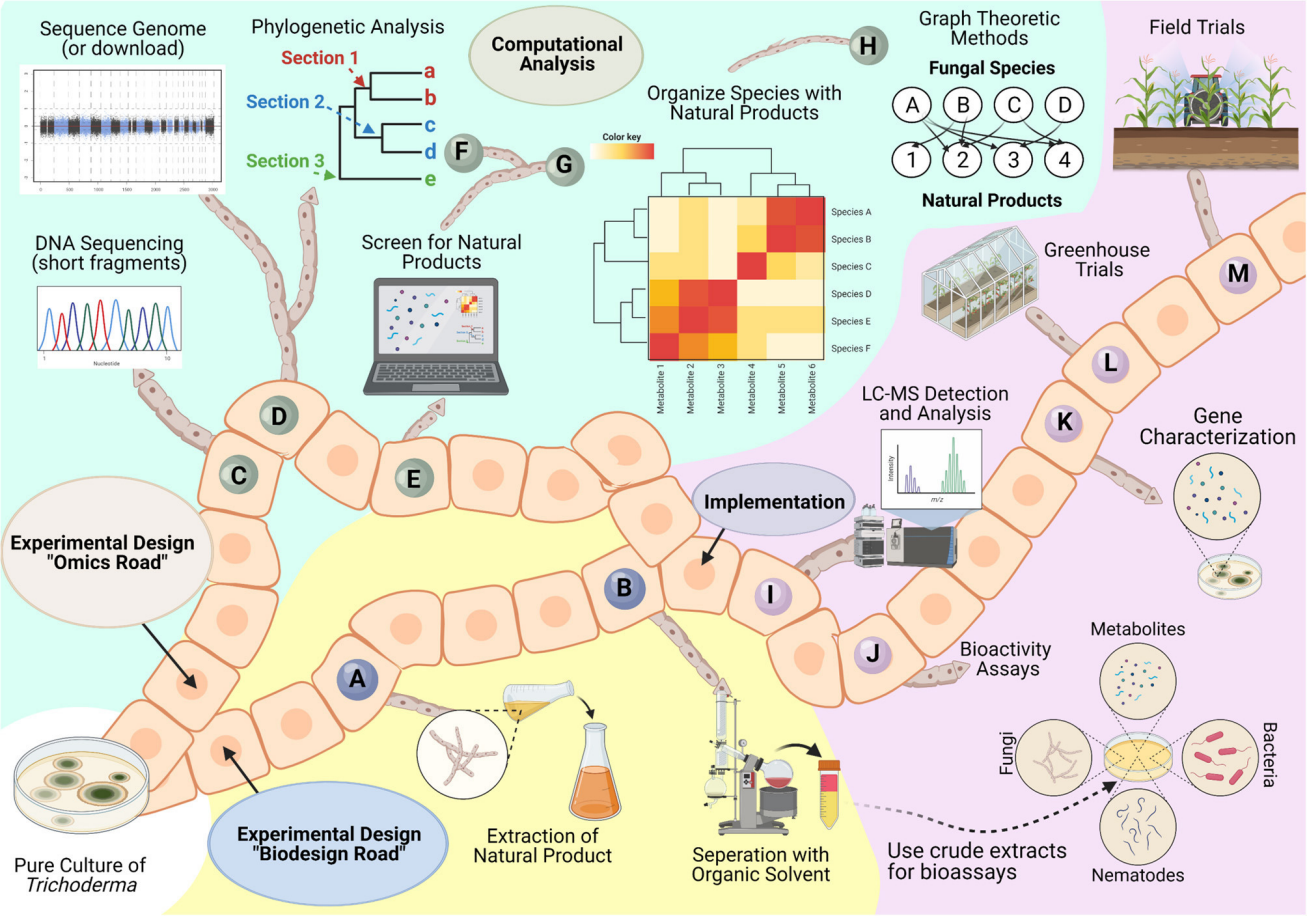
Roadmap for bioprospecting *Trichoderma* for natural products. Isolation of an axenic *Trichoderma* isolate is needed to initiate the roadmap. The biodesign road details the following general steps to extract and experimentally screen bioactive compounds: **(A)** Grow the culture in a liquid broth medium and collect the supernatant by filtration; **(B)** Use an appropriate organic solvent (e.g., ethyl acetate, methanol, etc.) to extract metabolites present in the filtrate. This crude exudate is composed of a mixture of natural products that can be further interrogated to identify targeted bioactivity properties. To predict putative natural products before extensive laboratory studies, then the “omics” road can be followed using the following steps: **(C)** Extract genomic DNA from the isolate and determine the species identity by amplifying and sequencing genetic markers like the internal transcribed spacer region (ITS), elongation factor 1-alpha (*tef1*), and RNA polymerase II subunit (*rbp2*) (Cai and Druzhinina, 2021); **(D)** Sequence reference genome of isolate; **(E)** Predict natural products using tools such as antiSMASH or amPEPpy. With this information, **(F**) comparative genomic analyses can be performed to determine which taxonomic section the target isolate belongs to, and **(G,H)** can be used to relate predictions across organisms to highlight the uniqueness and diversity of putative natural products across available *Trichoderma* by using graph theory. Both roads are led to an implementation step where experimental design aims to **(I)** selectively characterize predicted or isolated compound(s) by liquid or gas chromatography coupled to mass spectrometry, **(J)** evaluate bioactivity for selected compound(s), **(K)** determine the genes and proteins responsible for the production of the compound(s), **(L)** conduct trials to determine its potential usefulness in greenhouse conditions, and **(M)** test the compound effects in field trials.

The graph-theoretic analysis suggests that the Brevicompactum section, followed by Harzianum, had the most influence on the data (Figure 9A). Interestingly, there were several AMPs identified by amPEPpy that fall into the categories of 94–80% match based on current definitions of AMPs (Figure 9B). The graph-theoretic analysis also suggests that section Brevicompactum, closely followed by Longibrachiatum, have the most prevalence of predicted proteins potentially involved in AMP biosynthesis and production compared to all the other sections examined (Figure 9C). As illustrated in Figure 9D, graph theory results further substantiate the prevalence of “glycoside hydrolase family,” “cytochrome P450 family,” and “glycosyltransferase.”

Overall, by using whole-genome sequencing data available on NCBI, we were able to demonstrate the utility of genome prediction tools to identify putative secondary metabolites and AMPs that warrant further interrogation. Next, we describe how these inferred natural products can be experimentally interrogated with or without a priori knowledge.

## Discussion

### Experimental Framework to Characterize Natural Products in *Trichoderma* Species

In general, competition assays and choice or no-choice assays are often used as a first step for comparing the effects of a fungus on other microbes (Köhl et al., 2019) and would be useful in identifying negative association between an organism and the metabolic substances produced by another microbe (Fravel, 1988; Köhl et al., 2019). It is necessary to also understand how a particular fungus or natural product behaves in greenhouse conditions, which can be used to explore host-microbe interactions (Gibson et al., 1999) and efficacy against pathogens, host range, and environmental conditions before field trials. Next, with appropriate permits in-hand, field trial can be conducted to know how a biological product will perform in natural conditions, that are inherently more complex and less predictable. Often, off-target effects against non-target species, like insects, vertebrates, and weeds, as well as potential toxicity and carcinogenic effects on humans can be examined (Headrick and Goeden, 2001).

**Figure 11:**
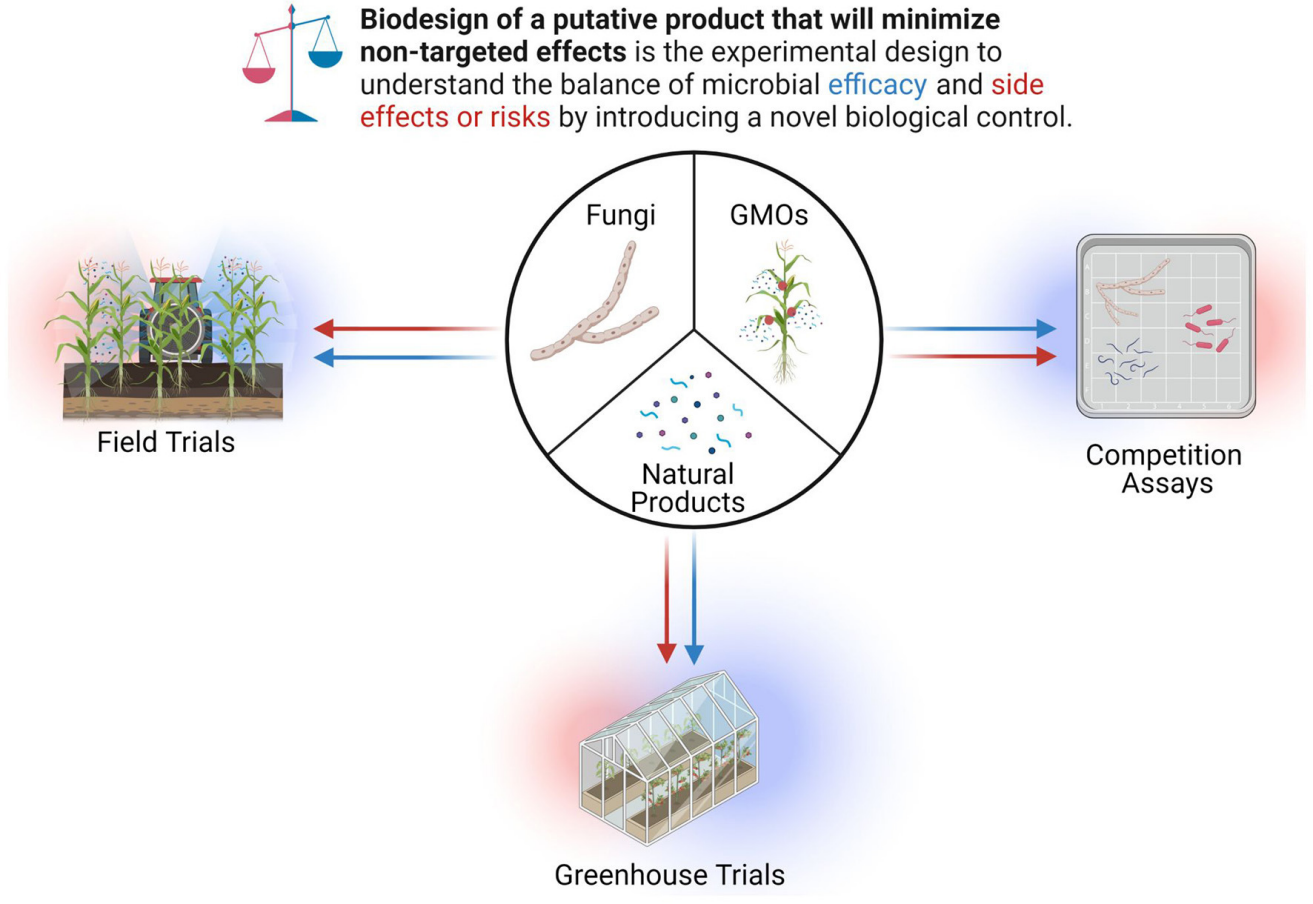
Biodesign of putative products to minimize untargeted effects.

Herein, we limit the scope of this entire process to an experimental framework for the rapid discovery and identification of natural product and therefore will not be describing topics related to greenhouse or field trials (Figure 11).

#### Extraction of Natural Products From *Trichoderma* Species

**Figure 12:**
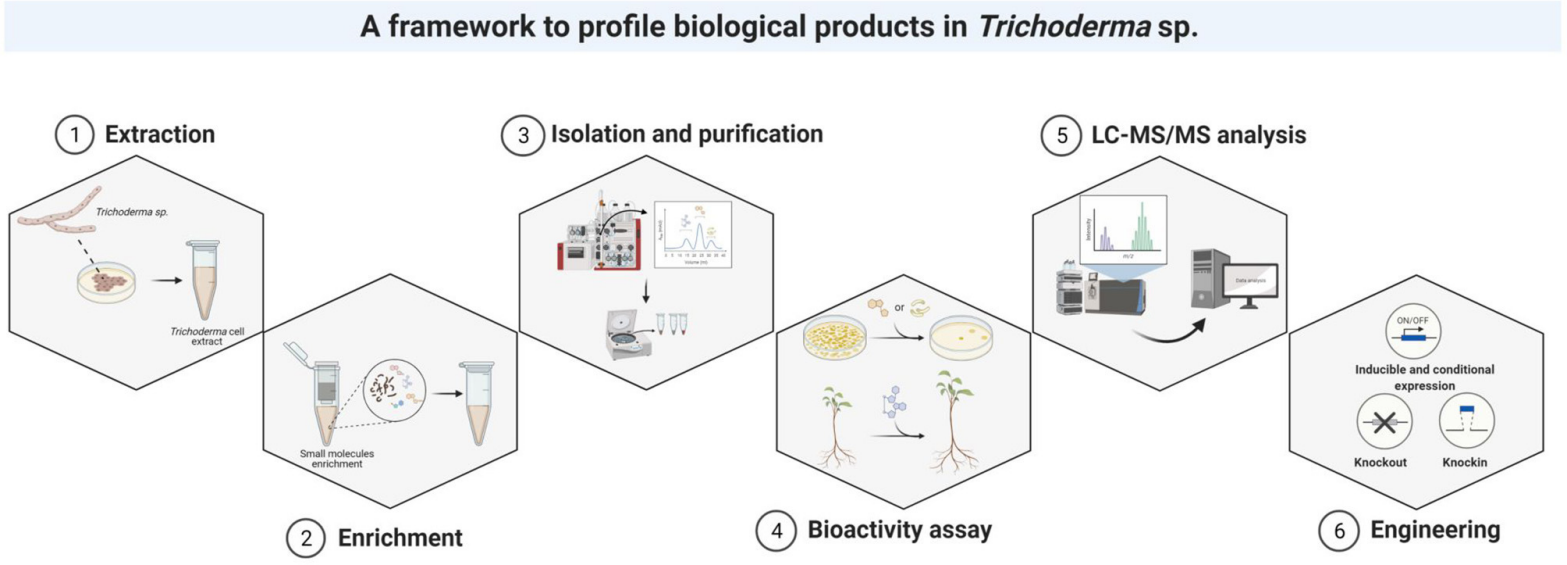
The framework for the identification of natural products in *Trichoderma* species.

Most *Trichoderma* isolates have been recovered from soils, from the fruiting bodies of other fungi, or from dead wood colonized by them (Druzhinina et al., 2011, Kubicek et al., 2019). The soil suspension, dilution, and aliquot methods are recommended for isolation of individual colonies. The most efficient substrate medium for the isolation and culture of *Trichoderma* isolates is potato dextrose agar supplemented with chloramphenicol, streptomycin, and rose bengal (Vargas Gil et al., 2009). Following the isolation and growth, the crude extract is collected to separate the desired natural products from the raw materials. The most common extraction methods are solvent extraction, distillation, pressing and sublimation (Zhang et al., 2018a) (Figure 12).

#### Enrichment, Isolation, and Purification of Natural Products

*Trichoderma* crude extracts contain a complex mixture of biological molecules, with the bioactive products representing <1% of the crude extract (Odendaal et al., 2011). The low abundance of naturally occurring biological products represents a major bottleneck in their discovery (Hanson and Howell, 2004; McMullin et al., 2017). To selectively isolate low-abundance biological products from the complex mixture, various enrichment strategies have been developed. Size exclusion ultrafiltration strategies, such as molecular weight cutoff spin column filters, represent common methods of enriching the low-molecular-weight fraction of a crude extract (Hanson and Howell, 2004; McMullin et al., 2017). In addition to molecular weight cutoff, gel-based separations, solvent extractions, and size exclusion chromatography are all frequently used for biological product enrichment (Rivera-Chávez et al., 2017; Song et al., 2018; Zhang et al., 2018a).

Although the enrichment step helps to increase the representation of low-abundance biological products, the resultant fraction may still have impurities and diverse biological molecules. Thus, this fraction can be further fractionated based on physicochemical properties (e.g., polarity, hydrophobicity, stability, solubility) to isolate and purify the specific target product with bioactivity (Rivera-Chávez et al., 2017). The fractionation step starts with separation and detection of the natural products *via* chromatographic methods followed by detection methods such as ultraviolet light detectors. Based on a specific retention time or window, natural products can be selectively or broadly collected, lyophilized, and resolubilized in the appropriate solvent and concentration. The detailed technologies and techniques for isolation and purification of natural products from microbes have been extensively reviewed and explained by previous methodology and review publications (Bucar et al., 2013; Joana Gil-Chávez et al., 2013; Gomes et al., 2017; Zhang et al., 2018a; Atanasov et al., 2021).

#### Bioactivity Assays of Natural Products

There are two primary routes that can be taken to apply the active ingredient of interest: using the whole fungus as the active ingredient or applying the natural product of the fungus, whether biosynthesized by the organism or chemically synthesized *in vitro*. In deciding between using the fungus or using the compound alone, it is useful to consider the mechanism by which it will carry out its effect.

Using the fungus itself, one mode of action is for the beneficial fungus to outcompete the pathogenic organisms for nutrients and space on the plant by colonizing the mutual areas (Ghorbanpour et al., 2018). For this to be effective, the fungus must have strong colonization capabilities and persist long enough to out-compete the pathogenic population. *Trichoderma* excels at root colonization because of its production of compounds such as hydrophobins, which allow its hyphal filaments to attach to a multitude of surfaces, aiding its ability to survive in the presence of other unwanted organisms (Guzmán-Guzmán et al., 2017). Furthermore, some *Trichoderma* species produce siderophores, which chelate iron, making it unavailable in the environment and potentially inhibiting the growth of competing organisms (Harman et al., 2004). In addition to the competition, *Trichoderma* can use mycoparasitic interactions to recognize and attack the parasite (Błaszczyk et al., 2014). One mechanism *Trichoderma* uses during its attack is to secrete various cell wall–degrading enzymes such as cellulase, pectinase, glucanase, lipase, and protease, which degrade several compounds, including chitin and glucan polysaccharides (Błaszczyk et al., 2014). Initially, lectins are used to recognize the pathogen and the hyphae coil around the pathogen and then penetrate the cell and use the enzymes previously mentioned to degrade the cell wall (Ghorbanpour et al., 2018). In addition to mycoparasitism, these cell wall degrading enzymes are involved in antibiosis, an antagonistic interaction in which *Trichoderma* produces these and other antimicrobial compounds, such as antibiotics and secondary metabolites, that exhibit a lethal effect on the parasitic organism (Błaszczyk et al., 2014); however, mycoparasitism and antibiosis may not be the primary mechanisms of biocontrol (Lorito et al., 2010). Included in this list of antimicrobials are compounds such as trichodermin, herizanolide, peptabiols, and epipolythiodioxopiperazines (Ghorbanpour et al., 2018). Two additional mechanisms include mycovirus-mediated cross-protection and induced systemic resistance. While these are different mechanisms—one is an infection with a non-virulent mycovirus and the other a beneficial organism inducing an immune response—they have similar overarching effects of priming/preparing the plant defense system for infection with pathogens (Ghorbanpour et al., 2018).

The other alternative is to use a natural product of the fungus as the active ingredient, which may allow for a more targeted outcome and circumvent the issues that may come with introducing a non-native organism to a new environment (Butt et al., 2001). These natural products, such as secondary metabolites and antimicrobial peptides, can also assist in the plant's defense mechanisms, function in an antibiotic manner, and compete against pathogens for nutrients such as metals (Keswani et al., 2019).

Once either the compound or the entire fungus is selected, a series of trials is necessary to validate its efficacy. For antimicrobial efficacy, it's important to establish a minimum inhibitory concentration with respect to the pathogenic organism targeted by the biocontrol agent and the commensal organisms that the product will encounter, determining if and how detrimental these are to them. To determine the minimum inhibitory concentration, the reconstituted samples from the fraction collectors in an appropriate solvent can be serially diluted and tested. Alternatively, a micro-titer broth dilution can be carried out (Aboobaker et al., 2019). This relatively easy screening method is a valuable tool, as any potential compounds can be immediately weeded out if they require an extremely high concentration to be successful. Additionally, dual-culture assays can be performed to determine the suppression of pathogenic growth directly (Lahlali and Hijri, 2010). Once initial screening has taken place, larger-scale experiments such as greenhouse trials can be carried out. During a greenhouse trial, data can be gathered on the effects of inoculating a plant of interest with the biocontrol agent; and preliminary conclusions will be reached regarding the overall health of the plant and any antagonistic effects (Perelló et al., 2003). Upon completion of greenhouse trials, larger-scale field experiments could be done to more closely mimic the conditions biocontrol agents encounter once they are deployed in nature. In these field experiments, larger-scale data, such as any effects on growth, seed production, and pathogen reduction, will be excellent indicators of market viability (Abdel-Fattah et al., 2007). Once the vetting process is completed and the biocontrol agent has been thoroughly assessed, mass production issues must be explored to ensure the product is effective and lucrative, including growth conditions of the fungus, synthetic synthesis of individual compounds vs. extraction from the fungus, formula optimization for prolonged shelf life, and determination of a dispersal method.

#### Natural Product Identification and Characterization

Following the detection and isolation of fractions with relevant bioactivity, biological product identification can be carried out with chromatography-coupled mass spectrometry (MS) for complex samples or direct injection/infusion for pure or low-complex mixtures. For chromatographic techniques, gas chromatography (GC) and liquid chromatography (LC) represent the common choices. GC is commonly used for volatile and polar small compounds, while LC allows the identification of mid-polar to non-polar metabolites. The choice of technique used can dictate what types of small molecules can be identified and this needs to be made on a case-by-case basis. The biological products predicted using prediction tools like antiSMASH and amPEPpy from the *Trichoderma* genome can help provide information needed to select suitable techniques for biological product profiling.

Alongside the chromatographic techniques, MS represents a key experimental platform that is applied in the discovery, and characterization of natural products. Generally, MS-based approaches used to measure natural products is broadly categorized as metabolomics. Metabolomics, either targeted or untargeted, can be used to identify the natural products in *Trichoderma*. The advantages and disadvantages of these approaches have been reviewed extensively (Roberts et al., 2012; Schrimpe-Rutledge et al., 2016). For the identification, quantification, and structural elucidation of natural products, MS data are searched against the spectral libraries.

A significant challenge in the sensitive identification of biological products *via* MS includes the lack of representative databases or spectral libraries. Thus, the characterization of biological products with the use of *de novo* search strategies (Cheng Q. et al., 2010). *De novo* sequencing algorithms derive biological product structures, molecular formulas, or sequences using only fragmented ionic information from the tandem mass spectra, thus providing the complete profiling of all biological products. For metabolomics, SIRIUS 4 is one of the leading *de novo* software, using deep neural networks for metabolite identification from high-resolution tandem MS data (Dührkop et al., 2019). It integrates high-resolution isotope pattern analysis and fragmentation trees with structural elucidation to provide a combined and coherent assessment of molecular structures from MS/MS data for large datasets (Dührkop et al., 2019). This tool allows the identification of the molecular formula of a query compound with very high accuracy; no spectral or structural databases are required for this step of the analysis because all theoretically possible molecular formulas are considered, allowing one to overcome the limitations of the current structural databases (Dührkop et al., 2019). For peptide-based biological products, PEAKS is well-established search software that also utilized machine-learning and combines *de novo* sequencing with traditional database searching (Ma et al., 2003; Zhang et al., 2012).

#### Genetic Engineering

Several transformation systems, relying on effective selectable markers, have been developed to genetically modify different *Trichoderma* species. The most widely used and optimized procedures for doing so are based on protoplasts (Gruber et al., 1990; Li et al., 2017) and *Agrobacterium tumefaciens*-mediated transformation (Zhong et al., 2007; Yang et al., 2011). Other less common genetic approaches are reported to be used in studying *Trichoderma*, such as Biolistics (gene gun) (Te'o et al., 2002), and electroporation (Wanka, 2021). The most investigated species in genetic engineering studies to produce enzymes or metabolites is *T. reesei* (Liu et al., 2015). Genetic transformation systems have also been developed for other *Trichoderma* species, including *T. harzianum* (Goldman et al., 1990; Zeilinger, 2004), *T. viride* (Manczinger et al., 1997), *T. atroviride* (Zeilinger, 2004; Calcáneo-Hernández et al., 2020), and *T. longibrachiatum* (Sánchez-Torres et al., 1994). In recent years, the CRISPR/Cas9-mediated genome editing system has been successfully employed in *T. reesei* and delivered in the format of either plasmid (Liu et al., 2015) or ribonucleoprotein complexes (Hao and Su, 2019). Finally, the transformation methods developed for *Trichoderma* species can be used for overexpression experiments on putative backbone genes or transcription factors as a strategy to increase the production and allow the characterization of specific metabolites produced in low amounts or not produced under standard culture conditions.

### Methods to Implement Computational Analysis Into Biological Products for Integrated Pest Management System

#### Fungi as an Active Ingredient

For numerous biological products, the active ingredient uses reactivated lyophilized cells of the fungus itself. To propose new *Trichoderma* species as active ingredients requires a fundamental understanding of the fungus behavior within an environment. Therefore, the approach should consider the fungus lifecycle, its interactions with other microbes and organisms, its responses to abiotic and biotic stresses, its pathogenicity behavior on cash crops, and its ability to promote the growth of competing weeds that can serve as vectors for other plant pathogens. Regarding *Trichoderma* species, several were shown to promote plant growth under abiotic and biotic stress conditions. Although all studies reviewed concentrated on how the fungus helped a plant in stress conditions, few or none focused on how stress affected the survivability of the fungus. The benefits of establishing a new species or strain as an active ingredient reside in getting more specific and long-lasting effects, cost-efficient disease management, and higher yields of cash crops. Particularly in *Trichoderma*, there is a strong history of species used as successful active ingredients (Figure 2, Supplementary Table 1). Another consideration is the effectiveness of a strain within a species or the efficacy of mixing different strains or species of *Trichoderma* and fungicides as formulations and active ingredients.

Importantly, due to the genetic diversity of *Trichoderma* strains, they can be unpredictable in terms of mechanisms and biocontrol improvement in different environmental settings (Benítez et al., 2004) and therefore should not be generalized at the species level. For this reason, companies use a particular strain in their products. Combinations of *Trichoderma* strains or species have been shown to better promote growth of cash crops in agriculture and forestry than a single strain or species (Chirino-Valle et al., 2016; Halifu et al., 2019; Chen et al., 2021). As such, there are several commercially available products with mixed strains or species of *Trichoderma* for better biocontrol, like Bioten® WP, BioWorks® Rootshield® *Plus*^+^ WP; Kiwivax®, Vinevax™ Bio-Dowel, and Vinevax™ Pruning Wound Dressing, to name a few (Supplementary Table 1). In addition, there are genetically altered hybrid strains between closely related *Trichoderma* species created by protoplast fusion (Stasz et al., 1988). These mutant strains had shown better biocontrol efficacy by selecting secondary metabolites or enzymes used for antimicrobial activity than individual species (Stasz, 1990; Hanson and Howell, 2002). However, to our knowledge, it is unknown if there are any commercial products or field experiments to test the efficacy of these modified fungal strains. Finally, mixing biological control agents (like *Trichoderma*) with fungicides (or derivatives of natural products) has been shown to reduce disease pressure (Ons et al., 2020). Since biological controls are dependent on environmental conditions, combining a biological product with fungicides can increase efficacy (Ons et al., 2020). However, researchers should examine if the *Trichoderma* strains are susceptible to the fungicide before using them in a mixed application. Moreover, there are few studies investigating biological products durability to control plant pathogens (Bardin et al., 2015), and it is known that there are pathogens resistant to some fungicides (Hahn, 2014; Hollomon, 2015). Therefore, further studies are needed to determine the durability and environmental sustainability by combining both biological products with fungicides.

Some disadvantages to applying active fungal strains in a field is that a fungus could sexually recombine with a native population, introducing unknown ecological consequences, or simply be incapable of surviving in the newly introduced environment. Thus, understanding the fungal lifecycle is critical to designing a new biological product. Fungal clones can expand their populations but still lack adaptability because of short-term survival mechanisms (Drenth et al., 2019). Sexual recombination is suitable for long-term survival and adaptability to a changing environment (Drenth et al., 2019). However, when non-native fungi capable of sexually reproducing are introduced into an environment, they can reproduce with a native population, creating hybridization and potentially having non-target effects. Hybridization has been shown in numerous pathogenic fungi and has led to the decline of several host crops or ornamental plants and targeting of new hosts (Schardl and Craven, 2003; Depotter et al., 2016; Stukenbrock, 2016). Alternatively, hybridization in symbiotic fungi has been beneficial for plant adaptation (particularly in grasses) for nutrient acquisition and stress conditions (Hamilton et al., 2010; Saari and Faeth, 2012; Oberhofer et al., 2014; Jia et al., 2016). Therefore, a screen of potential mating type genes within a species should be considered with the proposition of knocking out the mating genes if possible.

Fungi, in particular *Trichoderma*, have been used as active ingredients in bioherbicides to control weeds; however, there are no reports of their natural products being used (Kremer, 2005; Triolet et al., 2020). There are ongoing investigations to understand the mechanisms involved in controlling weed populations without affecting the crop of interest (Harding and Raizada, 2015). The main limiting factors for using fungi as bioherbicides are their narrow host range, their specific biotic requirements for good efficacy, and their possible non-targeted effects (Kremer, 2005). Therefore, the extraction of compounds possessing herbicide properties from these fungi can be a fruitful endeavor for overcoming these limitations (Kremer, 2005; Harding and Raizada, 2015; Triolet et al., 2020).

In the promising bioactive ingredient from a *Trichoderma* species (Figure 3), much bioactivity has been observed in the laboratory. However, the potential for transferring these species from the lab to the field is vague.

#### Natural Products as an Active Ingredient

Natural products deriving from microbes have also been used as active ingredients (Singh and Yadav, 2020) primarily those released during antibiosis interactions. Antibiosis is the secretion of natural products (e.g., secondary metabolites, AMPs) that have antimicrobial properties in the vicinity of other microbes (Howell, 1998). Secreted molecules might be the key to triggering a response from a host plant or *Trichoderma* itself. For example, the production of peptides or small molecules from a pathogenic fungus and its perception through G protein-coupled receptors might trigger a mycoparasitism response in *Trichoderma* species (Druzhinina et al., 2011). Several classes of molecules secreted by *Trichoderma*, like xylanases, peptaibols, swollenin, and cerato-platanins, have been reported to act as microbe-associated molecular patterns that trigger immunity in plants (Howell, 2003; Druzhinina et al., 2011; Newman et al., 2013). Owing to the difficulty of mass-producing biological products using fungi as active ingredients, and the agrochemical effects on the environment, natural products appear as promising solutions for implementation in integrated pest management (Dayan et al., 2009; Yan et al., 2018; Singh and Yadav, 2020; Triolet et al., 2020). To propose a new natural product as an active ingredient, a fundamental understanding of the molecule chemistry and behavior within an environment is needed. The main benefits of this approach are that these natural products could be used in combination with existing pesticides with known identities, bioactivities, and effects on plant growth promotion and defenses. An example of a natural product used in pesticides is the quinone outside inhibitor (Q_o_I) family of fungicides derived from the group of natural products called “strobilurin” (Balba, 2007; Vincelli, 2012). This group was first isolated from the wood-rotting fungus *Strobilurus tenacellus* (Anke et al., 1977). Q_o_I fungicides inhibit mitochondrial responses by binding at the Q_o_ site of the cytochrome bc1 enzyme complex. Q_o_I fungicides are a preventative measure through translaminar movement and are known to result in plant growth enhancements (Balba, 2007; Vincelli, 2012). Q_o_I fungicides pose less risk to human health than alternative pesticides (Balba, 2007; Vincelli, 2012). Unfortunately, there are resistant Q_o_I fungicide populations because of disease pressure in growers' fields, creating a need for alternative sources of natural products (https://www.frac.info/). As shown in Figure 5 and Table 2, *Trichoderma citrinoviridie, T. virens*, and *T. viride* all have BGCs with a 50% sequence match, and *T. atroviride* and *T. koningiopsis* both have BGCs with a 37% match with the cluster encoding the putative metabolite Ascochlorin. Berry et al. (2010) have demonstrated that Ascoholorin, isolated from the pathogenic fungus *Ascochyta viciae*, is a cyctochrome bc1 inhibitor acting on both Qi and Qo sites. Currently, Ascoholorin has not been identified or characterized in *Trichoderma* species. However, based on our bioinformatic results, there might possibly be an Ascoholorin-like compound in *Trichoderma* species, which could be investigated as a biopesticide.

To our knowledge, there are minimal caveats for introducing natural products into the environment; yet they might present some unknown non-targeted effects on beneficial microbes, livestock, and humans. Natural products are potentially unstable in extremely cold temperatures; therefore, they may not overwinter in the field, allowing more pathogenic inoculum to increase. They might also become denatured after long exposure to environmental factors, such as ultraviolet radiation.

#### Genetically Modified Crops With Biosynthetic Gene Clusters

Genetically modified organisms (GMOs) are engineered to produce a natural product by inserting a gene of interest into the target plant genome. This can enhance a plant's ability to fight against pathogenic insects and resist herbicides. The implementation of GMO crops with natural products derived from microbes started over 30 years ago. Currently, several GMO crops engineered with natural products, such as corn, soybeans, papaya, tomatoes, rice, and cotton, are available. The world's first GMO crop, FLAVR SAVR, was a virus-resistant tomato variety that suppressed the enzyme polygalacturonase, which dissolves cell-wall pectin and causes the fruit to become soft (Bruening and Lyons, 2000). Currently, three GMO crops are commonly used: *Bacillus thuringiensis* corn (Bt corn), Roundup Ready crops, and the Rainbow papaya, all approved by the United States Environmental Protection Agency and the United States Department of Agriculture. Bt corn is a hybrid plant bioengineered with a broad-spectrum insecticide in which the active ingredient is crystal (Cry) protein toxins (Gewin, 2003; Koch et al., 2015). Roundup Ready crops (soybeans, corn, alfalfa, cotton, and sorghum), genetically modified to contain a protein-coding for a glyphosate-insensitive enzyme, can kill competing weeds and reduce the source of inoculum for many plant pathogens. The Rainbow papaya has a gene from a papaya ringspot virus inserted into the fruit to make it resistant to the same virus, using the same logic as vaccination in mammalians.

Previous successes have shown that producing natural biological products directly from GMO crops is a feasible procedure. To our knowledge, there are no reports of inserting BGCs from *Trichoderma* into GMO crops. Thus, this idea may represent a new niche to explore in agriculture. However, caution is essential in creating GMO crops, as there are many rules and regulations to follow depending on the agricultural system for each country.

## Conclusions

**Figure 13:**
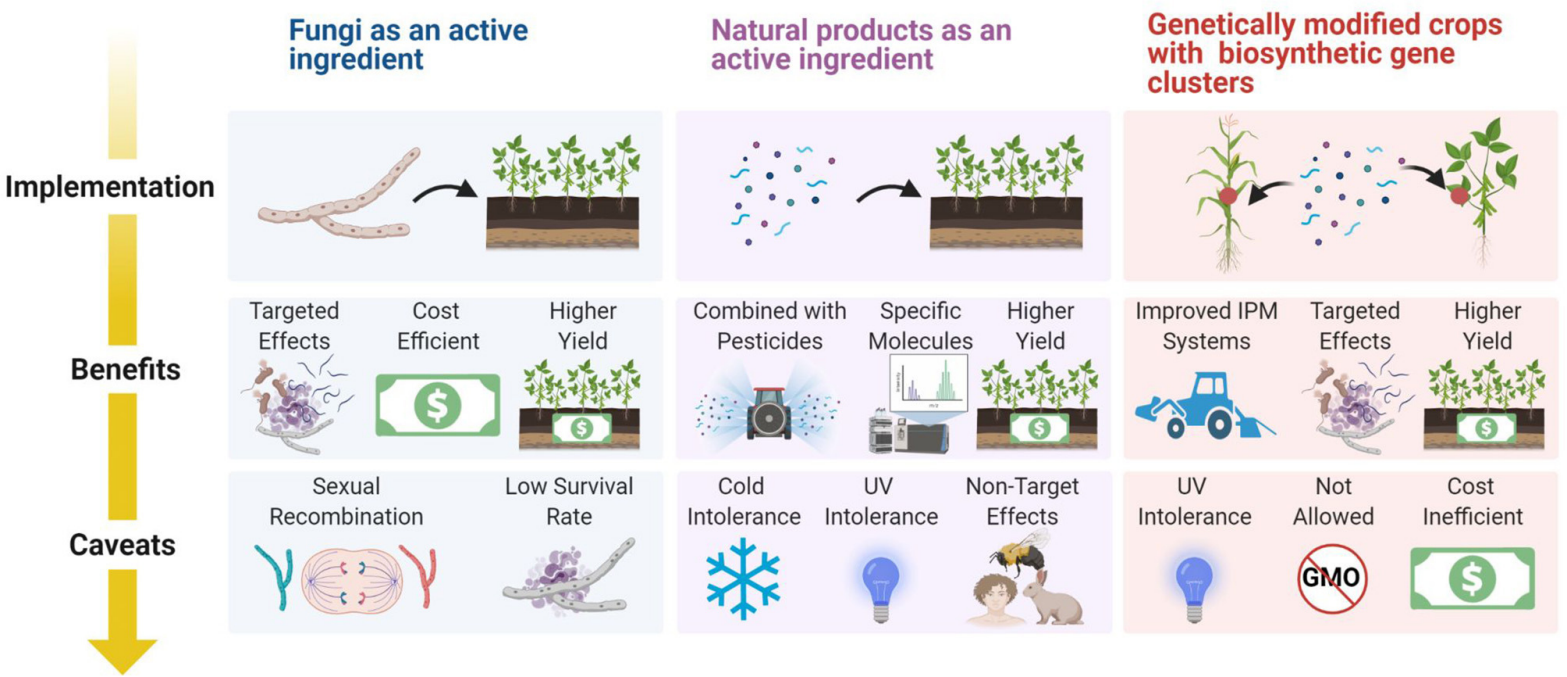
Considerations for how to introduce biological products from *Trichoderma* into field applications.

The success of any biological product will rely on its efficacy, cost efficiency, stability, and profitability. Our roadmap outlines the multifaceted abilities of *Trichoderma* as the prominent biological control agent. In addition, we demonstrate how to use omics and machine learning to identify new or existing products that can be used in the market and how to extract and implement those products while listing the benefits and caveats (Figure 13).

However, efficiently, and effectively translating laboratory observations to field applications should be linked to a purpose tied to a specific market or reason. For example, renewable and sustainable biofuels and bioproducts from plant biomass are a steadily growing industry representing alternative fuel sources to petroleum. Identifying novel or existing *Trichoderma* species or natural products to help promote terrestrial plants for higher biofuel and bioproduct production or the promotion of associated beneficial microbes can improve efforts to advance the use of bioenergy. Experiments have shown that *Trichoderma* species can improve the growth parameters of bioenergy crops like hybrid grasses (Chirino-Valle et al., 2016). In addition, there is a need to understand how using biocontrol products to influence environmental microbiome processes that drive global carbon cycling will impact climate change. Biological control could be a way to biologically mitigate heavy metals, radionuclides, or agrochemicals present in the soil, coastal sediments, freshwater, and subsurfaces.

Another example is sustainable agriculture, where the results should address farmers' and industry partners' needs and concerns. Farmers will adopt sustainable programs when there is a short-term economic outcome and if doing so benefits their farms, the environment, or both (Piñeiro et al., 2020). Ultimately, any biological control implementation should follow the goals of integrated pest management—economic viability, environmental safety, and social acceptability. These goals address the needs and concerns of the producer, the seller, and the consumer (Dara, 2019). For some engineered products, like GMOs, an understanding of the geopolitical climate is necessary for developing research and economic plans.

As outlined in this systematic review, new issues will arise as more biological products are developed, marketed, and implemented into agricultural or environmental settings. We have observed that several known compounds have been identified from *Trichoderma* species. However, these results do not translate into active ingredients available on the market. We suggest that the implementation team involved in developing products should be multidisciplinary. For example, for a promising biological product to be marketed, an ideal implementation team should include, but not be limited to, researchers that know the market/economy, chemistry, computational analysis, and the integral dynamics of an agricultural microcosm (e.g., plants, microbes, insects, etc.). Lastly, there are numerous new publications that are made yearly based on novel features from global studies on *Trichoderma* isolates. With this extensive dataset, there is need for additional high-quality genomes and representation of the various sections within *Trichoderma*. Altogether, this comprehensive roadmap can serve as guidance for the future production of biological products from *Trichoderma* and could be used for other fungal systems.

## Data Availability Statement

The original contributions presented in the study are included in the article/Supplementary Materials, further inquiries can be directed to the corresponding authors.

## Author Contributions

TR, HS, MS, JL, and PA designed research. TR, HS, MG, JE, MS, JL, and PA performed research and wrote the manuscript. MG conducted graph-theory analysis. JE and TR conducted phylogeny analysis. TR and HS analyzed antiSMASH results. TR and MS analyzed amPEPpy results. All authors contributed to the article and approved the submitted version.

## Funding

This research was sponsored by the Genomic Science Program, US Department of Energy (DOE), Office of Science, Biological and Environmental Research, as part of the Secure Ecosystem Engineering and Design and the Plant Microbe Interfaces Scientific Focus Areas at the Oak Ridge National Laboratory (ORNL). ORNL was managed by UT-Battelle LLC for DOE under contract DE-AC05-00OR22725. To perform the graph-theoretic analysis, MG used the resources of the Oak Ridge Leadership Computing Facility at ORNL, which was supported by the DOE Office of Science under Contract No. DE-AC05-00OR22725.

## Conflict of Interest

The authors declare that the research was conducted in the absence of any commercial or financial relationships that could be construed as a potential conflict of interest.

## Licenses and Permissions

This manuscript has been authored by UT-Battelle, LLC, under contract DE-AC05-00OR22725 with the US Department of Energy (DOE). The US government retains and the publisher, by accepting the article for publication, acknowledges that the US government retains a nonexclusive, paid-up, irrevocable, worldwide license to publish or reproduce the published form of this manuscript, or allow others to do so, for US government purposes. DOE will provide public access to these results of federally sponsored research in accordance with the DOE Public Access Plan (http://energy.gov/downloads/doe-public-access-plan).

## Publisher's Note

All claims expressed in this article are solely those of the authors and do not necessarily represent those of their affiliated organizations, or those of the publisher, the editors and the reviewers. Any product that may be evaluated in this article, or claim that may be made by its manufacturer, is not guaranteed or endorsed by the publisher.

## Supplementary Material

Supplementary Table 1 List of products that uses *Trichoderma* strains used as an active ingredient.

Supplementary Table 2 List of accession numbers and probability to be an AMP for each *Trichoderma* species examined with amPEPpy.

## References

[B1] Abd-Allah E. F. Ezzat S. M. (2005). Natural occurrences of citrinin in rice grains and its biocontrol by *Trichoderma hamatum*. Phytoparasitica 33, 73–84. 10.1007/BF02980928

[B2] Abdel-Fattah G. M. Shabana Y. M. Ismail A. E. Rashad Y. M. (2007). *Trichoderma harzianum*: a biocontrol agent against *Bipolaris oryzae*. Mycopathologia 164, 81–89. 10.1007/s11046-007-9032-9 17592758

[B3] Aboobaker Z. Viljoen A. Chen W. Crous P. W. Maharaj V. J. van Vuuren S. (2019). Endophytic fungi isolated from *Pelargonium sidoides* DC: antimicrobial interaction and isolation of a bioactive compound. South African J. Bot. 122, 535–542. 10.1016/j.sajb.2019.01.011

[B4] Abraham W. R. Meyer H. Abate D. (1995). Curvupallides, a new class of alkaloids from the fungus *Curvularia pallescens*. Tetrahedron 51, 4947–4952. 10.1016/0040-4020(95)98692-B

[B5] Aguilar-Pontes M. V. de Vries R. P. Zhou M. (2014). (Post-)Genomics approaches in fungal research. Brief. Funct. Genomics 13, 424–439. 10.1093/bfgp/elu028 25037051

[B6] Allison A. C. Kowalski W. J. Muller C. D. Eugui E. M. (1993). Mechanisms of action of mycophenolic acid. Ann. N. Y. Acad. Sci. 696, 63–87. 10.1111/j.1749-6632.1993.tb17143.x 7906496

[B7] Anke T. Oberwinkler F. Steglich W. Schramm G. (1977). The strobilurins - new antifungal antibiotics from the basidiomycete *Strobilurus tenacellus*. J. Antibiot. 30, 806–810. 10.7164/antibiotics.30.806 563391

[B8] Arinbasarova A. Y. Baskunov B. P. Medentsev A. G. (2017). A low-molecular mass antimicrobial peptide from *Trichoderma* cf. *aureoviride* Rifai VKM F-4268D. Microbiol. Russian Fed. 86, 289–291. 10.1134/S0026261717020059 30299885

[B9] Ariza M. R. Larsen T. O. Petersen B. O. Duus J. Barrero A. F. (2002). *Penicillium digitatum* metabolites on synthetic media and citrus fruits. J. Agric. Food Chem. 50, 6361–6365. 10.1021/jf020398d 12381117

[B10] Arnold S. L. Panaccione D. G. (2017). Biosynthesis of the pharmaceutically important fungal ergot alkaloid dihydrolysergic acid requires a specialized allele of cloA. Appl. Environ. Microbiol. 83. 10.1128/AEM.00805-17 28476772 PMC5494617

[B11] Atanasov A. G. Zotchev S. B. Dirsch V. M. Orhan I. E. Banach M. Rollinger J. M. . (2021). Natural products in drug discovery: advances and opportunities. Nat. Rev. Drug Discov. 20, 200–216. 10.1038/s41573-020-00114-z 33510482 PMC7841765

[B12] Atanasova L. Jaklitsch W. M. Komoń-Zelazowska M. Kubicek C. P. Druzhinina I. S. (2010). Clonal species *Trichoderma parareesei* sp. nov. likely resembles the ancestor of the cellulase producer Hypocrea jecorina/T. reesei. Appl. Environ. Microbiol. 76, 7259–7267. 10.1128/AEM.01184-10 20817800 PMC2976259

[B13] Baker S. E. Perrone G. Richardson N. M. Gallo A. Kubicek C. P. (2012). Phylogenomic analysis of polyketide synthase-encoding genes in Trichoderma. Microbiology 158, 147–154. 10.1099/mic.0.053462-0 22096146

[B14] Balba H. (2007). Review of strobilurin fungicide chemicals. J. Environ. Sci. Heal. Part B Pestic. Food Contam. Agric. Wastes 42, 441–451. 10.1080/03601230701316465 17474024

[B15] Bale J. S. Van Lenteren J. C. Bigler F. (2008). Biological control and sustainable food production. Philos. Trans. R. Soc. B Biol. Sci. 363, 761–776. 10.1098/rstb.2007.2182 17827110 PMC2610108

[B16] Bardin M. Ajouz S. Comby M. Lopez-Ferber M. Graillot B. Siegwart M. . (2015). Is the efficacy of biological control against plant diseases likely to be more durable than that of chemical pesticides? Front. Plant Sci. 6:566. 10.3389/fpls.2015.00566 26284088 PMC4515547

[B17] Baroncelli R. Zapparata A. Piaggeschi G. Sarrocco S. Vannacci G. (2016). Draft whole-genome sequence of *Trichoderma gamsii* T6085, a promising biocontrol agent of Fusarium head blight on wheat. Genome Announc. 4:e01747-15. 10.1128/genomeA.01747-15 26893428 PMC4759075

[B18] Barratt B. I. P. Moran V. C. Bigler F. van Lenteren J. C. (2018). The status of biological control and recommendations for improving uptake for the future. Biocontrology 63, 155–167. 10.1007/s10526-017-9831-y

[B19] Begum N. Qin C. Ahanger M. A. Raza S. Khan M. I. Ashraf M. . (2019). Role of arbuscular mycorrhizal fungi in plant growth regulation: implications in abiotic stress tolerance. Front. Plant Sci. 10:1068. 10.3389/fpls.2019.01068 31608075 PMC6761482

[B20] Benítez T. Rincón A. M. Limón M. C. Codón A. C. (2004). Biocontrol mechanisms of *Trichoderma* strains. Int. Microbiol. 7, 249–260. 10.2436/im.v7i4.9480 15666245

[B21] Benjamin E. O. Wesseler J. H. H. (2016). A socioeconomic analysis of biocontrol in integrated pest management: a review of the effects of uncertainty, irreversibility and flexibility. NJAS Wageningen J. Life Sci. 77, 53–60. 10.1016/j.njas.2016.03.002

[B22] Bergstrom J. D. Kurtz M. M. Rew D. J. Amend A. M. Karkas J. D. Bostedor R. G. . (1993). Zaragozic acids: a family of fungal metabolites that are picomolar competitive inhibitors of squalene synthase. Proc. Natl. Acad. Sci. U.S.A. 90, 80–84. 10.1073/pnas.90.1.80 8419946 PMC45603

[B23] Berlemont R. Martiny A. C. (2016). Glycoside hydrolases across environmental microbial communities. PLoS Comput. Biol. 12:e1005300. 10.1371/journal.pcbi.1005300 27992426 PMC5218504

[B24] Berry E. A. Huang L. Lee D. W. Daldal F. Nagai K. Minagawa N. (2010). Ascochlorin is a novel, specific inhibitor of the mitochondrial cytochrome bc1 complex. Biochim. Biophys. Acta - Bioenerg. 1797, 360–370. 10.1016/j.bbabio.2009.12.003 20025846 PMC2819552

[B25] Biasetto C. R. Somensi A. Sordi R. Chapla V. M. Ebrahimi S. N. Silva G. H. . (2020). The new koninginins T-U from *Phomopsis stipata*, an endophytic fungus isolated from *Styrax camporum* Pohl. Phytochem. Lett. 36, 106–110. 10.1016/j.phytol.2020.01.019

[B26] Bills G. F. Peláez F. Polishook J. D. Diez-Matas M. T. Harris G. H. Clapp W. H. . (1994). Distribution of zaragozic acids (squalestatins) among filamentous ascomycetes. Mycol. Res. 98, 733–739. 10.1016/S0953-7562(09)81046-0

[B27] Błaszczyk L. Siwulski M. Sobieralski K. Lisiecka J. Jedryczka M. (2014). *Trichoderma* spp. - application and prospects for use in organic farming and industry. J. Plant Prot. Res. 54, 309–317. 10.2478/jppr-2014-0047

[B28] Blin K. Shaw S. Steinke K. Villebro R. Ziemert N. Lee S. Y. . (2019). AntiSMASH 5.0: Updates to the secondary metabolite genome mining pipeline. Nucleic Acids Res. 47, W81–W87. 10.1093/nar/gkz310 31032519 PMC6602434

[B29] Bokesch H. R. Cartner L. K. Fuller R. W. Wilson J. A. Henrich C. J. Kelley J. A. . (2010). Inhibition of ABCG2-mediated drug efflux by naphthopyrones from marine crinoids. Bioorganic Med. Chem. Lett. 20, 3848–3850. 10.1016/j.bmcl.2010.05.057 20627559 PMC2924950

[B30] Bonsch B. Belt V. Bartel C. Duensing N. Koziol M. Lazarus C. M. . (2016). Identification of genes encoding squalestatin S1 biosynthesis and: *in vitro* production of new squalestatin analogues. Chem. Commun. 52, 6777–6780. 10.1039/c6cc02130a 27056201

[B31] Bruening G. Lyons M. J. (2000). The case of the FLAVR SAVR tomato. Calif. Agric. 54, 6–7.

[B32] Bucar F. Wube A. Schmid M. (2013). Natural product isolation-how to get from biological material to pure compounds. Nat. Prod. Rep. 30, 525–545. 10.1039/c3np20106f 23396532

[B33] Butt T. M. Jackson C. Magan N. (2001). Fungi as Biocontrol Agents: Progress, Problems, and Potential. Wallingford: CABI.

[B34] Caboche S. Pupin M. Leclère V. Fontaine A. Jacques P. Kucherov G. (2008). NORINE: a database of nonribosomal peptides. Nucleic Acids Res. 36:D326. 10.1093/nar/gkm792 17913739 PMC2238963

[B35] Cai F. Druzhinina I. S. (2021). In honor of John Bissett: authoritative guidelines on molecular identification of *Trichoderma*. Fungal Divers 107, 1–69. 10.1007/s13225-020-00464-4

[B36] Calcáneo-Hernández G. Rojas-Espinosa E. Landeros-Jaime F. Cervantes-Chávez J. A. Esquivel-Naranjo E. U. (2020). An efficient transformation system for *Trichoderma atroviride* using the pyr4 gene as a selectable marker. Brazil. J. Microbiol. 51, 1631–1643. 10.1007/s42770-020-00329-7 32627116 PMC7688867

[B37] Cardoza R. E. Malmierca M. G. Hermosa M. R. Alexander N. J. McCormick S. P. Proctor R. H. . (2011). Identification of loci and functional characterization of trichothecene biosynthesis genes in filamentous fungi of the genus *Trichoderma*. Appl. Environ. Microbiol. 77, 4867–4877. 10.1128/AEM.00595-11 21642405 PMC3147405

[B38] Castrillo M. L. Bich G. ángel Modenutti C. Turjanski A. Zapata P. D. Villalba L. L. (2017). First whole-genome shotgun sequence of a promising cellulase secretor, *Trichoderma koningiopsis* strain POS7. Genome Announc. 5:e00823-17. 10.1128/genomeA.00823-17 28912309 PMC5597750

[B39] Cerrini S. Lamba D. Scatturin A. Rossi C. Ughetto G. (1989). The crystal and molecular structure of the α-helical nonapeptide antibiotic leucinostatin A. Biopolymers 28, 409–420. 10.1002/bip.360280138 2720117

[B40] Chavali A. K. Rhee S. Y. (2018). Bioinformatics tools for the identification of gene clusters that biosynthesize specialized metabolites. Brief. Bioinformatics 19, 1022–1034. 10.1093/bib/bbx020 28398567 PMC6171489

[B41] Chaverri P. Branco-Rocha F. Jaklitsch W. Gazis R. Degenkolb T. Samuels G. J. (2015). Systematics of the *Trichoderma harzianum* species complex and the re-identification of commercial biocontrol strains. Mycologia 107, 558–590. 10.3852/14-147 25661720 PMC4885665

[B42] Chen D. Hou Q. Jia L. Sun K. (2021). Combined use of two *Trichoderma* strains to promote growth of pakchoi (*Brassica chinensis* L.). Agronomy 11:726. 10.3390/agronomy11040726

[B43] Chen M. Arato M. Borghi L. Nouri E. Reinhardt D. (2018). Beneficial services of arbuscular mycorrhizal fungi – from ecology to application. Front. Plant Sci. 9:1270. 10.3389/fpls.2018.01270 30233616 PMC6132195

[B44] Chen M. Liu Q. Gao S. S. Young A. E. Jacobsen S. E. Tang Y. (2019). Genome mining and biosynthesis of a polyketide from a biofertilizer fungus that can facilitate reductive iron assimilation in plant. Proc. Natl. Acad. Sci. U.S.A. 116, 5499–5504. 10.1073/pnas.1819998116 30842286 PMC6431147

[B45] Cheng M. J. Wu M. Der Chen I. S. Chen C. Y. Lo W. L. Yuan G. F. (2010). Secondary metabolites from the red mould rice of *Monascus purpureus* BCRC 38113. Nat. Prod. Res. 24, 1719–1725. 10.1080/14786410902941477 19459082

[B46] Cheng Q. Cao Y. Jiang C. Xu L. Wang M. Zhang S. . (2010). Identifying secreted proteins of *Marssonina brunnea* by degenerate PCR. Proteomics 10, 2406–2417. 10.1002/pmic.200900844 20391531

[B47] Chirino-Valle I. Kandula D. Littlejohn C. Hill R. Walker M. Shields M. . (2016). Potential of the beneficial fungus *Trichoderma* to enhance ecosystem-service provision in the biofuel grass *Miscanthus* × *giganteus* in agriculture. Sci. Rep. 6, 1–8. 10.1038/srep25109 27117716 PMC4846873

[B48] Contreras-Cornejo H. A. Macías-Rodríguez L. Del-Val E. Larsen J. (2016). Ecological functions of *Trichoderma* spp. and their secondary metabolites in the rhizosphere: interactions with plants. FEMS Microbiol. Ecol. 92:fiw036. 10.1093/femsec/fiw036 26906097

[B49] Costa J. H. Bazioli J. M. Barbosa L. D. Dos Santos Júnior P. L. T. Reis F. C. G. Klimeck T. . (2021). Phytotoxic tryptoquialanines produced *in vivo* by *Penicillium digitatum* are exported in extracellular vesicles. MBio 12, 1–16. 10.1128/mBio.03393-20 33563828 PMC7885104

[B50] Costa J. H. Bazioli J. M. de Vilhena Araújo E. Vendramini P. H. de Freitas Porto M. C. Eberlin M. N. . (2019a). Monitoring indole alkaloid production by *Penicillium digitatum* during infection process in citrus by Mass Spectrometry Imaging and molecular networking. Fungal Biol. 123, 594–600. 10.1016/j.funbio.2019.03.002 31345413

[B51] Costa J. H. Wassano C. I. Angolini C. F. F. Scherlach K. Hertweck C. Pacheco Fill T. (2019b). Antifungal potential of secondary metabolites involved in the interaction between citrus pathogens. Sci. Rep. 9, 1–11. 10.1038/s41598-019-55204-9 31819142 PMC6901458

[B52] Daguerre Y. Edel-Hermann V. Steinberg C. (2017). Fungal genes and metabolites associated with the biocontrol of soil-borne plant pathogenic fungi, in Fungal Metabolites, eds Mérillon J. M. Ramawat K. G. (New York, NY: Springer International Publishing), 33–104.

[B53] Dara S. K. (2019). The new integrated pest management paradigm for the modern age. J. Integr. Pest Manag. 10, 1–9. 10.1093/jipm/pmz010

[B54] Davis V. M. Stack M. E. (1994). Evaluation of alternariol and alternariol methyl ether for mutagenic activity in *Salmonella typhimurium*. Appl. Environ. Microbiol. 60, 3901–3902. 10.1128/aem.60.10.3901-3902.1994 7986060 PMC201908

[B55] Dayan F. E. Cantrell C. L. Duke S. O. (2009). Natural products in crop protection. Bioorganic Med. Chem. 17, 4022–4034. 10.1016/j.bmc.2009.01.046 19216080

[B56] De Cesare G. B. Cristy S. A. Garsin D. A. Lorenz M. C. (2020). Antimicrobial peptides: a new frontier in antifungal therapy. MBio 11, 1–21. 10.1128/mBio.02123-20 33144376 PMC7642678

[B57] Degenkolb T. Dieckmann R. Nielsen K. F. Gräfenhan T. Theis C. Zafari D. . (2008a). The *Trichoderma* Brevicompactum clade: a separate lineage with new species, new peptaibiotics, and mycotoxins. Mycol. Prog. 7, 177–219. 10.1007/s11557-008-0563-3

[B58] Degenkolb T. von Döhren H. Nielsen K. F. Samuels G. J. Brückner H. (2008b). Recent advances and future prospects in peptaibiotics, hydrophobin, and mycotoxin research, and their importance for chemotaxonomy of *Trichoderma* and *Hypocrea*. Chem. Biodivers. 5, 671–680. 10.1002/cbdv.200890064 18493954

[B59] Depotter J. R. L. Seidl M. F. Wood T. A. Thomma B. P. H. J. (2016). Interspecific hybridization impacts host range and pathogenicity of filamentous microbes. Curr. Opin. Microbiol. 32, 7–13. 10.1016/j.mib.2016.04.005 27116367

[B60] Derntl C. Guzmán-Chávez F. Mello-de-Sousa T. M. Busse H. J. Driessen A. J. M. Mach R. L. . (2017). *In vivo* study of the sorbicillinoid gene cluster in *Trichoderma reesei*. Front. Microbiol. 8:2037. 10.3389/fmicb.2017.02037 29104566 PMC5654950

[B61] Dhillon B. Feau N. Aerts A. L. Beauseigle S. Bernier L. Copeland A. . (2015). Horizontal gene transfer and gene dosage drives adaptation to wood colonization in a tree pathogen. Proc. Natl. Acad. Sci. U.S.A. 112, 3451–3456. 10.1073/pnas.1424293112 25733908 PMC4371944

[B62] Dias D. A. Urban S. Roessner U. (2012). A historical overview of natural products in drug discovery. Metabolites 2, 303–336. 10.3390/metabo2020303 24957513 PMC3901206

[B63] Donzelli B. G. G. Krasnoff S. B. Churchill A. C. L. Vandenberg J. D. Gibson D. M. (2010). Identification of a hybrid PKS-NRPS required for the biosynthesis of NG-391 in *Metarhizium robertsii*. Curr. Genet. 56, 151–162. 10.1007/s00294-010-0288-0 20355253

[B64] Dotson B. R. Soltan D. Schmidt J. Areskoug M. Rabe K. Swart C. . (2018). The antibiotic peptaibol alamethicin from *Trichoderma* permeabilises *Arabidopsis* root apical meristem and epidermis but is antagonised by cellulase-induced resistance to alamethicin. BMC Plant Biol. 18:165. 10.1186/s12870-018-1370-x 30097019 PMC6086028

[B65] Doughari J. (2015). The occurrence, properties and significance of citrinin mycotoxin. J. Plant Pathol. Microbiol. 6:321. 10.4172/2157-7471.1000321

[B66] Drenth A. McTaggart A. R. Wingfield B. D. (2019). Fungal clones win the battle, but recombination wins the war. IMA Fungus 10, 1–6. 10.1186/s43008-019-0020-8 32647622 PMC7325676

[B67] Druzhinina I. Kubicek C. P. (2005). Species concepts and biodiversity in *Trichoderma* and *Hypocrea*: from aggregate species to species clusters. J. Zhejiang Univ. Sci. 6B, 100–112. 10.1631/jzus.2005.B0100 15633245 PMC1389624

[B68] Druzhinina I. S. Chenthamara K. Zhang J. Atanasova L. Yang D. Miao Y. . (2018). Massive lateral transfer of genes encoding plant cell wall-degrading enzymes to the mycoparasitic fungus *Trichoderma* from its plant-associated hosts. PLoS Genet. 14:e1007322. 10.1371/journal.pgen.1007322 29630596 PMC5908196

[B69] Druzhinina I. S. Komoń-Zelazowska M. Atanasova L. Seidl V. Kubicek C. P. (2010). Evolution and ecophysiology of the industrial producer *Hypocrea jecorina* (Anamorph *Trichoderma reesei*) and a new sympatric agamospecies related to it. PLoS ONE 5:e9191. 10.1371/journal.pone.0009191 20169200 PMC2820547

[B70] Druzhinina I. S. Komoń-Zelazowska M. Kredics L. Hatvani L. Antal Z. Belayneh T. . (2008). Alternative reproductive strategies of *Hypocrea orientalis* and genetically close but clonal *Trichoderma Iongibrachiatum*, both capable of causing invasive mycoses of humans. Microbiology 154, 3447–3459. 10.1099/mic.0.2008/021196-0 18957598

[B71] Druzhinina I. S. Kubicek C. P. (2017). Genetic engineering of *Trichoderma reesei* cellulases and their production. Microb. Biotechnol. 10, 1485–1499. 10.1111/1751-7915.12726 28557371 PMC5658622

[B72] Druzhinina I. S. Seidl-Seiboth V. Herrera-Estrella A. Horwitz B. A. Kenerley C. M. Monte E. . (2011). *Trichoderma*: the genomics of opportunistic success. Nat. Rev. Microbiol. 9, 749–759. 10.1038/nrmicro2637 21921934

[B73] Dubos R. J. (1939). Studies on a bactericidal agent extracted from a soil bacillus: I. Preparation of the agent. Its activity *in vitro*. J. Exp. Med. 70, 1–10. 10.1084/jem.70.1.1 19870884 PMC2133784

[B74] Dubos R. J. Cattaneo C. (1939). Studies on a bactericidal agent extracted from a soil bacillus iii. Preearation and activity of a protein-free fraction. J. Exp. Med. 70, 249–256. 10.1084/jem.70.3.249 19870906 PMC2133820

[B75] Dührkop K. Fleischauer M. Ludwig M. Aksenov A. A. Melnik A. V. Meusel M. . (2019). SIRIUS 4: a rapid tool for turning tandem mass spectra into metabolite structure information. Nat. Methods 16, 299–302. 10.1038/s41592-019-0344-8 30886413

[B76] Ebead G. A. Overy D. P. Berrué F. Kerr R. G. (2012). *Westerdykella reniformis* sp. nov., producing the antibiotic metabolites melinacidin IV and chetracin B. IMA Fungus 3, 189–201. 10.5598/imafungus.2012.03.02.11 23355972 PMC3539322

[B77] El-Sayed A. M. Mitchell V. J. Suckling D. M. (2014). 6-Pentyl-2H-pyran-2-one: a potent peach-derived kairomone for New Zealand Flower Thrips, *Thrips obscuratus*. J. Chem. Ecol. 40, 50–55. 10.1007/s10886-014-0379-3 24435661

[B78] Endo A. Hasumi K. Yamada A. Shimoda R. Takeshima H. (1986). The synthesis of compactin (ml-236b) and monacolin k in fungi. J. Antibiot. 39, 1609–1610. 10.7164/antibiotics.39.1609 3793631

[B79] Evidente A. Cimmino A. Andolfi A. Vurro M. Zonno M. C. Cantrell C. L. . (2008). Phyllostictines A-D, oxazatricycloalkenones produced by *Phyllosticta cirsii*, a potential mycoherbicide for *Cirsium arvense* biocontrol. Tetrahedron 64, 1612–1619. 10.1016/j.tet.2007.12.010

[B80] Fanelli F. Liuzzi V. C. Logrieco A. F. Altomare C. (2018). Genomic characterization of *Trichoderma atrobrunneum* (*T. harzianum* species complex) ITEM 908: insight into the genetic endowment of a multi-target biocontrol strain. BMC Genomics 19:662. 10.1186/s12864-018-5049-3 30200883 PMC6131884

[B81] Faure D. (2002). The family-3 glycoside hydrolases: from housekeeping functions to host-microbe interactions. Appl. Environ. Microbiol. 68, 1485–1490. 10.1128/AEM.68.4.1485-1490.2002 11916659 PMC123870

[B82] Fedorova N. D. Moktali V. Medema M. H. (2012). Bioinformatics approaches and software for detection of secondary metabolic gene clusters. Methods Mol. Biol. 944, 23–45. 10.1007/978-1-62703-122-6_2 23065606

[B83] Felnagle E. A. Jackson E. E. Chan Y. A. Podevels A. M. Berti A. D. McMahon M. D. . (2008). Nonribosomal peptide synthetases involved in the production of medically relevant natural products. Mol. Pharm. 5, 191–211. 10.1021/mp700137g 18217713 PMC3131160

[B84] Finking R. Marahiel M. A. (2004). Biosynthesis of nonribosomal peptides. Annu. Rev. Microbiol. 58, 453–488. 10.1146/annurev.micro.58.030603.123615 15487945

[B85] Flissi A. Ricart E. Campart C. Chevalier M. Dufresne Y. Michalik J. . (2020). Norine: update of the nonribosomal peptide resource. Nucleic Acids Res. 48, D465–D469. 10.1093/nar/gkz1000 31691799 PMC7145658

[B86] Fravel D. R. (1988). Role of antibiosis in the biocontrol of plant diseases. Annu. Rev. Phytopathol. 26, 75–91.

[B87] Fujita T. Wada S. I. Iida A. Nishimura T. Kanai M. Toyama N. (1994). Fungal metabolites. XIII. Isolation and structural elucidation of new peptaibols, Trichodecenins-I and -II, from *Trichoderma viride*. Chem. Pharm. Bull. 42, 489–494. 10.1248/cpb.42.489 8004694

[B88] Gao X. Chooi Y. H. Ames B. D. Wang P. Walsh C. T. Tang Y. (2011). Fungal indole alkaloid biosynthesis: genetic and biochemical investigation of the tryptoquialanine pathway in *Penicillium aethiopicum*. J. Am. Chem. Soc. 133, 2729–2741. 10.1021/ja1101085 21299212 PMC3045477

[B89] Gauthier T. Wang X. Dos Santos J. Fysikopoulos A. Tadrist S. Canlet C. . (2012). Trypacidin, a spore-borne toxin from *Aspergillus fumigatus*, is cytotoxic to lung cells. PLoS ONE 7:e0029906. 10.1371/journal.pone.0029906 22319557 PMC3272003

[B90] Geisler K. Hughes R. K. Sainsbury F. Lomonossoff G. P. Rejzek M. Fairhurst S. . (2013). Biochemical analysis of a multifunctional cytochrome P450 (CYP51) enzyme required for synthesis of antimicrobial triterpenes in plants. Proc. Natl. Acad. Sci. U.S.A. 110, E3360–E3367. 10.1073/pnas.1309157110 23940321 PMC3761579

[B91] Gewin V. (2003). Genetically modified corn - environmental benefits and risks. PLoS Biol. 1:e8. 10.1371/journal.pbio.0000008 14551906 PMC212689

[B92] Ghorbanpour M. Omidvari M. Abbaszadeh-Dahaji P. Omidvar R. Kariman K. (2018). Mechanisms underlying the protective effects of beneficial fungi against plant diseases. Biol. Control 117, 147–157. 10.1016/j.biocontrol.2017.11.00622623151

[B93] Gibson D. J. Connolly J. Hartnett D. C. Weidenhamer J. D. (1999). Designs for greenhouse studies of interactions between plants. J. Ecol. 87, 1–16. 10.1046/j.1365-2745.1999.00321.x

[B94] Goldman G. H. Van Montagu M. Herrera-Estrella A. (1990). Transformation of *Trichoderma harzianum* by high-voltage electric pulse. Curr. Genet. 17, 169–174. 10.1007/BF00312863

[B95] Golo P. S. Gardner D. R. Grilley M. M. Takemoto J. Y. Krasnoff S. B. Pires M. S. . (2014). Production of destruxins from *Metarhizium* spp. fungi in artificial medium and in endophytically colonized cowpea plants. PLoS ONE 9:e104946. 10.1371/journal.pone.0104946 25127450 PMC4134251

[B96] Gomes A. R. Duarte A. C. Rocha-Santos T. A. P. (2017). Analytical techniques for discovery of bioactive compounds from marine fungi, in Fungal Metabolites, eds Mérillon J. M. Ramawat K. G. (New York, NY: Springer International Publishing), 415–434.

[B97] Grigoriev I. V. Nikitin R. Haridas S. Kuo A. Ohm R. Otillar R. . (2014). MycoCosm portal: gearing up for 1000 fungal genomes. Nucleic Acids Res. 42, D699–704. 10.1093/nar/gkt1183 24297253 PMC3965089

[B98] Grindrod P. Parsons M. C. Higham D. J. Estrada E. (2011). Communicability across evolving networks. Phys. Rev. E Stat. Nonlinear Soft Matter Phys. 83:046120. 10.1103/PhysRevE.83.046120 21599253

[B99] Gruber F. Visser J. Kubicek C. P. de Graaff L. H. (1990). The development of a heterologous transformation system for the cellulolytic fungus *Trichoderma reesei* based on a pyrG-negative mutant strain. Curr. Genet. 18, 71–76. 10.1007/BF00321118 2245476

[B100] Guo K. Sui Y. Li Z. Huang Y. Zhang H. (2020). *Trichoderma viride* Tv-1511 colonizes arabidopsis leaves and promotes arabidopsis growth by modulating the MAP kinase 6-mediated activation of plasma membrane H+-ATPase. J. Plant Growth Regul. 39, 1261–1276. 10.1007/s00344-019-10063-6

[B101] Guo Y. Ghirardo A. Weber B. Schnitzler J. P. Philipp Benz J. Rosenkranz M. (2019). *Trichoderma* species differ in their volatile profiles and in antagonism toward ectomycorrhiza Laccaria bicolor. Front. Microbiol. 10:891. 10.3389/fmicb.2019.00891 31105677 PMC6499108

[B102] Gupta S. Ellis S. E. Ashar F. N. Moes A. Bader J. S. Zhan J. . (2014). Transcriptome analysis reveals dysregulation of innate immune response genes and neuronal activity-dependent genes in autism. Nat. Commun. 5, 1–8. 10.1038/ncomms6748 25494366 PMC4270294

[B103] Gutiérrez S. McCormick S. P. Cardoza R. E. Lindo L. Alexander N. J. Proctor R. H. (2020). *Trichoderma* trichothecenes, in New and Future Developments in Microbial Biotechnology and Bioengineering, eds Gupta V. G. Rodriguez-Couto S. (New York, NY: Elsevier), 281–301.

[B104] Guzmán-Guzmán P. Alemán-Duarte M. I. Delaye L. Herrera-Estrella A. Olmedo-Monfil V. (2017). Identification of effector-like proteins in *Trichoderma* spp. and role of a hydrophobin in the plant-fungus interaction and mycoparasitism. BMC Genet. 18:16. 10.1186/s12863-017-0481-y 28201981 PMC5310080

[B105] Haas H. (2014). Fungal siderophore metabolism with a focus on *Aspergillus fumigatus*. Nat. Prod. Rep. 31, 1266–1276. 10.1039/c4np00071d 25140791 PMC4162504

[B106] Hahn M. (2014). The rising threat of fungicide resistance in plant pathogenic fungi: botrytis as a case study. J. Chem. Biol. 7, 133–141. 10.1007/s12154-014-0113-1 25320647 PMC4182335

[B107] Halifu S. Deng X. Song X. Song R. (2019). Effects of two *Trichoderma* strains on plant growth, rhizosphere soil nutrients, and fungal community of *Pinus sylvestris* var. mongolica annual seedlings. Forests 10, 1–17. 10.3390/f10090758

[B108] Halliwell G. Griffin M. (1973). The nature and mode of action of the cellulolytic component C1 of *Trichoderma koningii* on native cellulose. Biochem. J. 135, 587–594. 10.1042/bj1350587 4798312 PMC1165873

[B109] Hamilton C. E. Dowling T. E. Faeth S. H. (2010). Hybridization in endophyte symbionts alters host response to moisture and nutrient treatments. Microb. Ecol. 59, 768–775. 10.1007/s00248-009-9606-9 19921327

[B110] Hanson L. E. Howell C. R. (2002). Biocontrol efficacy and other characteristics of protoplast fusants between *Trichoderma koningii* and *T. virens*. Mycol. Res. 106, 321–328. 10.1017/S0953756202005592

[B111] Hanson L. E. Howell C. R. (2004). Elicitors of plant defense responses from biocontrol strains of *Trichoderma virens*. Phytopathology 94, 171–176. 10.1094/PHYTO.2004.94.2.171 18943540

[B112] Hao Z. Su X. (2019). Fast gene disruption in *Trichoderma reesei* using *in vitro* assembled Cas9/gRNA complex. BMC Biotechnol. 19. 10.1186/s12896-018-0498-y 30626373 PMC6325762

[B113] Harding D. P. Raizada M. N. (2015). Controlling weeds with fungi, bacteria and viruses: a review. Front. Plant Sci. 6:659. 10.3389/fpls.2015.00659 26379687 PMC4551831

[B114] Harman G. E. (2006). Overview of mechanisms and uses of *Trichoderma* spp. Phytopathology 96, 190–194. 10.1094/PHYTO-96-01 18943924

[B115] Harman G. E. Howell C. R. Viterbo A. Chet I. Lorito M. (2004). *Trichoderma* species - Opportunistic, avirulent plant symbionts. Nat. Rev. Microbiol. 2, 43–56. 10.1038/nrmicro797 15035008

[B116] Hauser D. Sigg H. P. (1971). Isolierung und Abbau von Sordarin. 1. Mitteilung über Sordarin. Helv. Chim. Acta 54, 1178–1190. 10.1002/hlca.19710540427 5095217

[B117] Hayat R. Ali S. Amara U. Khalid R. Ahmed I. (2010). Soil beneficial bacteria and their role in plant growth promotion: a review. Ann. Microbiol. 60, 579–598. 10.1007/s13213-010-0117-1

[B118] Headrick D. H. Goeden R. D. (2001). Biological control as a tool for ecosystem management. Biol. Control 21, 249–257. 10.1006/bcon.2001.0939

[B119] Hertweck C. (2009). Hidden biosynthetic treasures brought to light. Nat. Chem. Biol. 5, 450–452. 10.1038/nchembio0709-450 19536102

[B120] Hinterdobler W. Li G. Spiegel K. Basyouni-Khamis S. Gorfer M. Schmoll M. (2021). *Trichoderma reesei* isolated from Austrian soil with high potential for biotechnological application. Front. Microbiol. 12:552301. 10.3389/fmicb.2021.552301 33584603 PMC7876326

[B121] Hollomon D. W. (2015). Fungicide resistance: 40 years on and still a major problem, in Fungicide Resistance in Plant Pathogens, eds Ishii H. Hollomon D. W. (Tokyo: Springer Japan), 3–11.

[B122] Howell C. R. (1998). The role of antibiosis in biocontrol. Trichoderma and Gliocladium 2, 173–184.

[B123] Howell C. R. (2003). Mechanisms employed by *Trichoderma* species in the biological control of plant diseases: the history and evolution of current concepts. Plant Dis. 87, 4–10. 10.1094/PDIS.2003.87.1.4 30812698

[B124] Huan Y. Kong Q. Mou H. Yi H. (2020). Antimicrobial peptides: classification, design, application and research progress in multiple fields. Front. Microbiol. 11:2559. 10.3389/fmicb.2020.582779 33178164 PMC7596191

[B125] Huang Q. Tezuka Y. Hatanaka Y. Kikuchi T. Nishi A. Tubaki K. (1995). Studies on metabolites of mycoparasitic fungi. IV. minor peptaibols of *Trichoderma koningii*. Chem. Pharm. Bull. 43, 1663–1667. 10.1248/cpb.43.1663 8536339

[B126] Huber F. M. (1967). Griseofulvin, in Mechanism of Action, eds Gottlieb D. Shaw P. D. (Berlin: Springer Berlin Heidelberg), 181–189.

[B127] Itoh Y. Kodama K. Furuya K. Takahashi S. Haneishi T. Takiguchi Y. . (1980). A new sesquiterpene antibiotic, heptel1dic acid producing organisms, fermentation, isolation and characterization. J. Antibiot. 33, 468–473. 10.7164/antibiotics.33.468 7191847

[B128] Jalal M. A. F. Love S. K. van der Helm D. (1988). Nα-Dimethylcoprogens: three novel trihydroxamate siderophores from pathogenic fungi. Biol. Met. 1, 4–8. 10.1007/BF01128011 2978957

[B129] Jayasuriya H. Silverman K. C. Zink D. L. Jenkins R. G. Sanchez M. Pelaez F. . (1998). Clavaric acid: a triterpenoid inhibitor of farnesyl-protein transferase from *Clavariadelphus truncatus*. J. Nat. Prod. 61, 1568–1570. 10.1021/np980200c 9868169

[B130] Jia T. Oberhofer M. Shymanovich T. Faeth S. H. (2016). Effects of hybrid and non-hybrid *Epichloë* endophytes and their associated host genotypes on the response of a native grass to varying environments. Microb. Ecol. 72, 185–196. 10.1007/s00248-016-0743-7 26909796

[B131] Joana Gil-Chávez G. Villa J. A. Fernando Ayala-Zavala J. Basilio Heredia J. Sepulveda D. Yahia E. M. . (2013). Technologies for extraction and production of bioactive compounds to be used as nutraceuticals and food ingredients: an overview. Compr. Rev. Food Sci. Food Saf. 12, 5–23. 10.1111/1541-4337.12005

[B132] Kaewchai S. Soytong K. Hyde K. D. (2009). Mycofungicides and fungal biofertilizers. Fungal Divers. 38, 25–50.

[B133] Keller N. P. (2019). Fungal secondary metabolism: regulation, function and drug discovery. Nat. Rev. Microbiol. 17, 167–180. 10.1038/s41579-018-0121-1 30531948 PMC6381595

[B134] Keller N. P. Turner G. Bennett J. W. (2005). Fungal secondary metabolism - from biochemistry to genomics. Nat. Rev. Microbiol. 3, 937–947. 10.1038/nrmicro1286 16322742

[B135] Kenshole E. Herisse M. Michael M. Pidot S. J. (2021). Natural product discovery through microbial genome mining. Curr. Opin. Chem. Biol. 60, 47–54. 10.1016/j.cbpa.2020.07.010 32853968

[B136] Keswani C. Mishra S. Sarma B. K. Singh S. P. Singh H. B. (2014). Unraveling the efficient applications of secondary metabolites of various *Trichoderma* spp. Appl. Microbiol. Biotechnol. 98, 533–544. 10.1007/s00253-013-5344-5 24276619

[B137] Keswani C. Singh H. B. Hermosa R. García-Estrada C. Caradus J. He Y. W. . (2019). Antimicrobial secondary metabolites from agriculturally important fungi as next biocontrol agents. Appl. Microbiol. Biotechnol. 103, 9287–9303. 10.1007/s00253-019-10209-2 31707442

[B138] Khaldi N. Collemare J. Lebrun M. H. Wolfe K. H. (2008). Evidence for horizontal transfer of a secondary metabolite gene cluster between fungi. Genome Biol. 9:R18. 10.1186/gb-2008-9-1-r18 18218086 PMC2395248

[B139] Khan I. Xie W. L. Yu Y. C. Sheng H. Xu Y. Wang J. Q. . (2020). Heteroexpression of *Aspergillus nidulans* laeA in marine-derived fungi triggers upregulation of secondary metabolite biosynthetic genes. Mar. Drugs 18:652. 10.3390/md18120652 33352941 PMC7766385

[B140] Kim J. Movassaghi M. (2015). Biogenetically-inspired total synthesis of epidithiodiketopiperazines and related alkaloids. Acc. Chem. Res. 48, 1159–1171. 10.1021/ar500454v 25843276 PMC4408872

[B141] Kingston D. G. I. (2011). Modern natural products drug discovery and its relevance to biodiversity conservation. J. Nat. Prod. 74, 496–511. 10.1021/np100550t 21138324 PMC3061248

[B142] Koch M. S. Ward J. M. Levine S. L. Baum J. A. Vicini J. L. Hammond B. G. (2015). The food and environmental safety of Bt crops. Front. Plant Sci. 6:283. 10.3389/fpls.2015.00283 25972882 PMC4413729

[B143] Köhl J. Kolnaar R. Ravensberg W. J. (2019). Mode of action of microbial biological control agents against plant diseases: relevance beyond efficacy. Front. Plant Sci. 10:845. 10.3389/fpls.2019.00845 31379891 PMC6658832

[B144] Krasnoff S. B. Keresztes I. Gillilan R. E. Szebenyi D. M. E. Donzelli B. G. G. Churchill A. C. L. . (2007). Serinocyclins A and B, cyclic heptapeptides from *Metarhizium anisopliae*. J. Nat. Prod. 70, 1919–1924. 10.1021/np070407i 18044842

[B145] Kremer R. J. (2005). The role of bioherbicides in weed management. Biopestic. Int. 1, 127–141.

[B146] Kroken S. Glass N. L. Taylor J. W. Yoder O. C. Turgeon B. G. (2003). Phylogenomic analysis of type I polyketide synthase genes in pathogenic and saprobic ascomycetes. Proc. Natl. Acad. Sci. U.S.A. 100, 15670–15675. 10.1073/pnas.2532165100 14676319 PMC307626

[B147] Krupke O. A. Castle A. J. Rinker D. L. (2003). The North American mushroom competitor, *Trichoderma aggressivum* cf. aggressivum, produces antifungal compounds in mushroom compost that inhibit mycelial growth of the commercial mushroom Agaricus bisporus. Mycol. Res. 107, 1467–1475. 10.1017/S0953756203008621 15000247

[B148] Kubicek C. P. Herrera-Estrella A. Seidl-Seiboth V. Martinez D. A. Druzhinina I. S. Thon M. . (2011). Comparative genome sequence analysis underscores mycoparasitism as the ancestral life style of *Trichoderma*. Genome Biol. 12, 1–15. 10.1186/gb-2011-12-4-r40 21501500 PMC3218866

[B149] Kubicek C. P. Steindorff A. S. Chenthamara K. Manganiello G. Henrissat B. Zhang J. . (2019). Evolution and comparative genomics of the most common *Trichoderma* species. BMC Genomics 20:485. 10.1186/s12864-019-5680-7 31189469 PMC6560777

[B150] Kudo F. Matsuura Y. Hayashi T. Fukushima M. Eguchi T. (2016). Genome mining of the sordarin biosynthetic gene cluster from *Sordaria araneosa* Cain ATCC 36386: Characterization of cycloaraneosene synthase and GDP-6-deoxyaltrose transferase. J. Antibiot. 69, 541–548. 10.1038/ja.2016.40 27072286

[B151] Kulimushi S. M. Muiru W. M. Mutitu E. W. (2021). Potential of *Trichoderma* spp., *Bacillus subtilis* and *Pseudomonas fluorescens* in the management of early blight in tomato. Biocontrol Sci. Technol. 10.1080/09583157.2021.1900784. [Epub ahead of print].

[B152] Kumar M. Ashraf S. (2017). Role of *Trichoderma* spp. as a biocontrol agent of fungal plant pathogens, in Probiotics and Plant Health, eds Kumar V. Kumar M. Sharma S. Prasad R. (Singapore: Springer), 497–506.

[B153] Lagashetti A. C. Dufossé L. Singh S. K. Singh P. N. (2019). Fungal pigments and their prospects in different industries. Microorganisms 7:604. 10.3390/microorganisms7120604 31766735 PMC6955906

[B154] Lahlali R. Hijri M. (2010). Screening, identification and evaluation of potential biocontrol fungal endophytes against *Rhizoctonia solani* AG3 on potato plants. FEMS Microbiol. Lett. 311, 152–159. 10.1111/j.1574-6968.2010.02084.x 20738401

[B155] Landeis A. Schmidt-Heydt M. (2021). Sequencing and analysis of the entire genome of the mycoparasitic fungus *Trichoderma afroharzianum*. Microbiol. Resour. Announc. 10:e00211-21. 10.1128/mra.00211-21 33858929 PMC8050971

[B156] Lawrence D. P. Kroken S. Pryor B. M. Arnold A. E. (2011). Interkingdom gene transfer of a hybrid NPS/PKS from bacteria to filamentous ascomycota. PLoS ONE 6:e28231. 10.1371/journal.pone.0028231 22140558 PMC3226686

[B157] Lawrence T. J. Carper D. L. Spangler M. K. Carrell A. A. Rush T. A. Minter S. J. . (2020). amPEPpy 1.0: a portable and accurate antimicrobial peptide prediction tool. Bioinformatics 3, 1–3. 10.1093/bioinformatics/btaa917 33135060

[B158] Lebe K. E. Cox R. J. (2019). Oxidative steps during the biosynthesis of squalestatin S1. Chem. Sci. 10, 1227–1231. 10.1039/c8sc02615g 30774923 PMC6349020

[B159] Lee S. Yap M. Behringer G. Hung R. Bennett J. W. (2016). Volatile organic compounds emitted by *Trichoderma* species mediate plant growth. Fungal Biol. Biotechnol. 3, 1–14. 10.1186/s40694-016-0025-7 28955466 PMC5611631

[B160] Li D. Tang Y. Lin J. Cai W. (2017). Methods for genetic transformation of filamentous fungi. Microb. Cell Fact. 16:168. 10.1186/s12934-017-0785-7 28974205 PMC5627406

[B161] Li J. Wu Y. Chen K. Wang Y. Hu J. Wei Y. . (2018). *Trichoderma cyanodichotomus* sp. nov., a new soil-inhabiting species with a potential for biological control. Can. J. Microbiol. 64, 1020–1029. 10.1139/cjm-2018-0224 30199653

[B162] Li N. Alfiky A. Wang W. Islam M. Nourollahi K. Liu X. . (2018). Volatile compound-mediated recognition and inhibition between *Trichoderma* biocontrol agents and *Fusarium oxysporum*. Front. Microbiol. 9:2614. 10.3389/fmicb.2018.02614 30455673 PMC6231246

[B163] Li M. F. Li G. H. Zhang K. Q. (2019). Non-volatile metabolites from *Trichoderma* spp. Metabolites 9:58. 10.3390/metabo9030058 30909487 PMC6468342

[B164] Liu R. Chen L. Jiang Y. Zhou Z. Zou G. (2015). Efficient genome editing in filamentous fungus *Trichoderma reesei* using the CRISPR/Cas9 system. Cell Discov. 1:15007. 10.1038/celldisc.2015.7 27462408 PMC4860831

[B165] Lorito M. Woo S. L. Harman G. E. Monte E. (2010). Translational research on *Trichoderma*: from'omics to the field. Annu. Rev. Phytopathol. 48, 395–417. 10.1146/annurev-phyto-073009-114314 20455700

[B166] Lu S. Tian J. Sun W. Meng J. Wang X. Fu X. . (2014). Bis-naphtho-γ-pyrones from fungi and their bioactivities. Molecules 19, 7169–7188. 10.3390/molecules19067169 24886942 PMC6270783

[B167] Ma B. Zhang K. Hendrie C. Liang C. Li M. Doherty-Kirby A. . (2003). PEAKs: Powerful software for peptide *de novo* sequencing by tandem mass spectrometry. Rapid Commun. Mass Spectrom. 17, 2337–2342. 10.1002/rcm.1196 14558135

[B168] Ma L. J. Fedorova N. D. (2010). A practical guide to fungal genome projects: strategy, technology, cost and completion. Mycology 1, 9–24. 10.1080/21501201003680943

[B169] Macheleidt J. Mattern D. J. Fischer J. Netzker T. Weber J. Schroeckh V. . (2016). Regulation and role of fungal secondary metabolites. Annu. Rev. Genet. 50, 371–392. 10.1146/annurev-genet-120215-035203 27732794

[B170] Macias F. A. Varela R. M. Simonet A. M. Cutler H. G. Cutler S. J. Eden M. A. . (2000). Bioactive carotanes from *Trichoderma virens*. J. Nat. Prod. 63, 1197–1200. 10.1021/np000121c 11000018

[B171] Mahlapuu M. Håkansson J. Ringstad L. Björn C. (2016). Antimicrobial peptides: an emerging category of therapeutic agents. Front. Cell. Infect. Microbiol. 6:194. 10.3389/fcimb.2016.00194 28083516 PMC5186781

[B172] Mahood E. H. Kruse L. H. Moghe G. D. (2020). Machine learning: a powerful tool for gene function prediction in plants. Appl. Plant Sci. 8. 10.1002/aps3.11376 32765975 PMC7394712

[B173] Malmierca M. G. Izquierdo-Bueno I. Mccormick S. P. Cardoza R. E. Alexander N. J. Moraga J. . (2016). Botrydial and botcinins produced by *Botrytis cinerea* regulate the expression of *Trichoderma arundinaceum* genes involved in trichothecene biosynthesis. Mol. Plant Pathol. 17, 1017–1031. 10.1111/mpp.12343 26575202 PMC6638445

[B174] Manczinger L. Komonyi O. Antal Z. Ferenczy L. (1997). A method for high-frequency transformation of *Trichoderma viride*. J. Microbiol. Methods 29, 207–210. 10.1016/S0167-7012(97)00026-2

[B175] Marfori E. C. Kajiyama S. Fukusaki E. I. Kobayashi A. (2002). Trichosetin, a novel tetramic acid antibiotic produced in dual culture of *Trichoderma harzianum* and *Catharanthus roseus* callus. Zeitschrift Naturforsch. Sect. C J. Biosci. 57, 465–470. 10.1515/znc-2002-5-611 12132686

[B176] Martinez D. Berka R. M. Henrissat B. Saloheimo M. Arvas M. Baker S. E. . (2008). Genome sequencing and analysis of the biomass-degrading fungus *Trichoderma reesei* (syn. *Hypocrea jecorina*). Nat. Biotechnol. 26, 553–560. 10.1038/nbt1403 18454138

[B177] Masi M. Meyer S. Górecki M. Mandoli A. Di Bari L. Pescitelli G. . (2017). Pyriculins A and B, two monosubstituted hex-4-ene-2,3-diols and other phytotoxic metabolites produced by *Pyricularia grisea* isolated from buffelgrass (*Cenchrus ciliaris*). Chirality 29, 726–736. 10.1002/chir.22744 28902437

[B178] Matsumoto M. Matsutani S. Sugita K. Yoshida H. Hayashi F. Terui Y. . (1992). Depudecin: a novel compound inducing the flat phenotypi of nih3t3 cells doubly transformed by ras-and src-oncogene, produced by *Alternaria brassicicola*. J. Antibiot. 45, 879–885. 10.7164/antibiotics.45.879 1500354

[B179] McInnes A. G. Smith D. G. Wat C. K. Vining L. C. Wright J. L. C. (1974). Tenellin and bassianin, metabolites of *Beauveria* species. Structure elucidation with 15N- and doubly 13C-enriched compounds using 13C nuclear magnetic resonance spectroscopy. J. Chem. Soc. Chem. Commun. 8, 281–282. 10.1039/C39740000281

[B180] McMullin D. R. Renaud J. B. Barasubiye T. Sumarah M. W. Miller J. D. (2017). Metabolites of *Trichoderma* species isolated from damp building materials. Can. J. Microbiol. 63, 621–632. 10.1139/cjm-2017-0083 28384416

[B181] Meca G. Soriano J. M. Gaspari A. Ritieni A. Moretti A. Mañes J. (2010). Antifungal effects of the bioactive compounds enniatins A, A1, B, B1. Toxicon 56, 480–485. 10.1016/j.toxicon.2010.04.013 20417654

[B182] Meena M. Swapnil P. Zehra A. Aamir M. Dubey M. K. Goutam J. . (2017). Beneficial microbes for disease suppression and plant growth promotion, in Plant-Microbe Interactions in Agro-Ecological Perspectives, eds Singh D. Singh H. Prabha R. (Singapore: Springer), 395–432.

[B183] Mendoza-Mendoza A. Zaid R. Lawry R. Hermosa R. Monte E. Horwitz B. A. . (2018). Molecular dialogues between *Trichoderma* and roots: role of the fungal secretome. Fungal Biol. Rev. 32, 62–85. 10.1016/j.fbr.2017.12.001

[B184] Meng J. Wang X. Xu D. Fu X. Zhang X. Lai D. . (2016). Sorbicillinoids from fungi and their bioactivities. Molecules 21:715. 10.3390/molecules21060715 27258245 PMC6273499

[B185] Migheli Q. González-Candelas L. Dealessi L. Camponogara A. Ramón-Vidal D. (1998). Transformants of *Trichoderma longibrachiatum* overexpressing the β-1,4-endoglucanase gene *egl1* show enhanced biocontrol of *Pythium ultimum* on cucumber. Phytopathology 88, 673–677. 10.1094/PHYTO.1998.88.7.673 18944939

[B186] Mistry J. Chuguransky S. Williams L. Qureshi M. Salazar G. A. Sonnhammer E. L. L. . (2021). Pfam: the protein families database in 2021. Nucleic Acids Res. 49, D412–D419. 10.1093/nar/gkaa913 33125078 PMC7779014

[B187] Montesinos E. (2007). Antimicrobial peptides and plant disease control. FEMS Microbiol. Lett. 270, 1–11. 10.1111/j.1574-6968.2007.00683.x 17371298

[B188] Moore J. H. Davis N. D. Diener U. L. (1972). Mellein and 4-hydroxymellein production by *Aspergillus ochraceus* Wilhelm. Appl. Microbiol. 23, 1067–1072. 10.1128/aem.23.6.1067-1072.1972 5064985 PMC380508

[B189] Mukherjee P. K. Horwitz B. A. Kenerley C. M. (2012). Secondary metabolism in *Trichoderma*- a genomic perspective. Microbiology 158, 35–45. 10.1099/mic.0.053629-0 21998165

[B190] Mukherjee P. K. Hurley J. F. Taylor J. T. Puckhaber L. Lehner S. Druzhinina I. . (2018). Ferricrocin, the intracellular siderophore of *Trichoderma virens*, is involved in growth, conidiation, gliotoxin biosynthesis and induction of systemic resistance in maize. Biochem. Biophys. Res. Commun. 505, 606–611. 10.1016/j.bbrc.2018.09.170 30278887

[B191] Mukhopadhyay R. Kumar D. (2020). *Trichoderma*: a beneficial antifungal agent and insights into its mechanism of biocontrol potential. Egypt. J. Biol. Pest Control 30, 1–8. 10.1186/s41938-020-00333-x

[B192] Neumann N. K. N. Stoppacher N. Zeilinger S. Degenkolb T. Brückner H. Schuhmacher R. (2015). The peptaibiotics database - a comprehensive online resource. Chem. Biodivers. 12, 743–751. 10.1002/cbdv.201400393 26010663

[B193] Newman D. J. Cragg G. M. (2020). Natural products as sources of new drugs over the nearly four decades from 01/1981 to 09/2019. J. Nat. Prod. 83, 770–803. 10.1021/acs.jnatprod.9b01285 32162523

[B194] Newman M. (2018). Networks. Oxford: Oxford University Press.

[B195] Newman M. A. Sundelin T. Nielsen J. T. Erbs G. (2013). MAMP (microbe-associated molecular pattern) triggered immunity in plants. Front. Plant Sci. 4:139. 10.3389/fpls.2013.00139 23720666 PMC3655273

[B196] Niehaus E. M. Kleigrewe K. Wiemann P. Studt L. Sieber C. M. K. Connolly L. R. . (2013). Genetic manipulation of the *Fusarium fujikuroi* fusarin gene cluster yields insight into the complex regulation and fusarin biosynthetic pathway. Chem. Biol. 20, 1055–1066. 10.1016/j.chembiol.2013.07.004 23932525

[B197] Nielsen K. F. Gräfenhan T. Zafari D. Thrane U. (2005). Trichothecene production by *Trichoderma brevicompactum*. J. Agric. Food Chem. 53, 8190–8196. 10.1021/jf051279b 16218663

[B198] Oberhofer M. Güsewell S. Leuchtmann A. (2014). Effects of natural hybrid and non-hybrid *Epichloë* endophytes on the response of *Hordelymus europaeus* to drought stress. New Phytol. 201, 242–253. 10.1111/nph.12496 24102453

[B199] Odendaal A. Y. Trader D. J. Carlson E. E. (2011). Chemoselective enrichment for natural products discovery. Chem. Sci. 2, 760–764. 10.1039/c0sc00620c 24926410 PMC4051302

[B200] Ogawa T. Ando K. Aotani Y. Shinoda K. Tanaka T. Tsukuda E. . (1995). RES-1214-1 and-2, novel non-peptidic endothelin type A receptor antagonists produced by *Pestalotiopsis* sp. J. Antibiot. 48, 1401–1406. 10.7164/antibiotics.48.1401 8557594

[B201] Ons L. Bylemans D. Thevissen K. Cammue B. P. A. (2020). Combining biocontrol agents with chemical fungicides for integrated plant fungal disease control. Microorganisms 8, 1–19. 10.3390/microorganisms8121930 33291811 PMC7762048

[B202] Page L. Brin S. (1998). The anatomy of a large-scale hypertextual Web search engine. Comput. Networks 30, 107–117. 10.1016/s0169-7552(98)00110-x

[B203] Parker S. R. Cutler H. G. Jacyno J. M. Hill R. A. (1997). Biological activity of 6-pentyl-2H-pyran-2-one and its analogs. J. Agric. Food Chem. 45, 2774–2776. 10.1021/jf960681a

[B204] Patron N. J. Waller R. F. Cozijnsen A. J. Straney D. C. Gardiner D. M. Nierman W. C. . (2007). Origin and distribution of epipolythiodioxopiperazine (ETP) gene clusters in filamentous ascomycetes. BMC Evol. Biol. 7, 1–15. 10.1186/1471-2148-7-174 17897469 PMC2045112

[B205] Pedras M. S. C. Irina Zaharia L. I. Ward D. E. (2002). The destruxins: synthesis, biosynthesis, biotransformation, and biological activity. Phytochemistry 59, 579–596. 10.1016/S0031-9422(02)00016-X 11867090

[B206] Perelló A. Mónaco C. Simón M. R. Sisterna M. Dal Bello G. (2003). Biocontrol efficacy of *Trichoderma* isolates for tan spot of wheat in Argentina. Crop Prot. 22, 1099–1106. 10.1016/S0261-2194(03)00143-1

[B207] Pfordt A. Schiwek S. Karlovsky P. von Tiedemann A. (2020). *Trichoderma afroharzianum* Ear Rot–a new disease on maize in Europe. Front. Agron. 2, 547758. 10.3389/fagro.2020.547758

[B208] Pham N. van Heck R. G. A. van Dam J. C. J. Schaap P. J. Saccenti E. Suarez-Diez M. (2019). Consistency, inconsistency, and ambiguity of metabolite names in biochemical databases used for genome-scale metabolic modelling. Metabolites 9:28. 10.3390/metabo9020028 30736318 PMC6409771

[B209] Pimentel D. Zuniga R. Morrison D. (2005). Update on the environmental and economic costs associated with alien-invasive species in the United States. Ecol. Econ. 52, 273–288. 10.1016/j.ecolecon.2004.10.002

[B210] Piñeiro V. Arias J. Dürr J. Elverdin P. Ibáñez A. M. Kinengyere A. . (2020). A scoping review on incentives for adoption of sustainable agricultural practices and their outcomes. Nat. Sustain. 3, 809–820. 10.1038/s41893-020-00617-y

[B211] Proctor R. H. McCormick S. P. Kim H. S. Cardoza R. E. Stanley A. M. Lindo L. . (2018). Evolution of structural diversity of trichothecenes, a family of toxins produced by plant pathogenic and entomopathogenic fungi. PLoS Pathog. 14:e1006946. 10.1371/journal.ppat.1006946 29649280 PMC5897003

[B212] Proksa B. Uhrín D. Liptaj T. Šturdíková M. (1998). Neosartorin, an ergochrome biosynthesized by *Neosartorya fischeri*. Phytochemistry 48, 1161–1164. 10.1016/S0031-9422(98)00169-1

[B213] Prosperini A. Berrada H. Ruiz M. J. Caloni F. Coccini T. Spicer L. J. . (2017). A review of the mycotoxin enniatin B. Front. Public Heal. 5:304. 10.3389/fpubh.2017.00304 29201864 PMC5697211

[B214] Pusztahelyi T. Holb I. J. Pócsi I. (2015). Secondary metabolites in fungus-plant interactions. Front. Plant Sci. : 573. 10.3389/fpls.2015.00573 PMC4527079 26300892

[B215] Reddy G. C. Goyal R. K. Puranik S. Waghmar V. Vikram K. V. Sruthy K. S. (2020). Biofertilizers toward sustainable agricultural development, in Plant Microbe Symbiosis, eds Varma A. Tripathi S. Prasad R. (Cham: Springer International Publishing), 115–128.

[B216] Reino J. L. Guerrero R. F. Hernández-Galán R. Collado I. G. (2008). Secondary metabolites from species of the biocontrol agent *Trichoderma*. Phytochem. Rev. 7, 89–123. 10.1007/s11101-006-9032-232457712

[B217] Reynolds H. T. Slot J. C. Divon H. H. Lysøe E. Proctor R. H. Brown D. W. (2017). Differential retention of gene functions in a secondary metabolite cluster. Mol. Biol. Evol. 34, 2002–2015. 10.1093/molbev/msx145 28460114

[B218] Rifai M. A. (1969). A revision of the genus *Trichoderma*. Mycol. Pap. 116, 1–116.

[B219] Rivera-Chávez J. Raja H. A. Graf T. N. Gallagher J. M. Metri P. Xue D. . (2017). Prealamethicin F50 and related peptaibols from *Trichoderma arundinaceum*: validation of their authenticity via *in situ* chemical analysis. RSC Adv. 7, 45733–45741. 10.1039/c7ra09602j 29379602 PMC5786278

[B220] Roberts L. D. Souza A. L. Gerszten R. E. Clish C. B. (2012). Targeted metabolomics. Curr. Protoc. Mol. Biol. 1:Unit30.2. 10.1002/0471142727.mb3002s98 22470063 PMC3334318

[B221] Rojo F. G. Reynoso M. M. Ferez M. Chulze S. N. Torres A. M. (2007). Biological control by *Trichoderma* species of *Fusarium solani* causing peanut brown root rot under field conditions. Crop Prot. 26, 549–555. 10.1016/j.cropro.2006.05.006

[B222] Rokas A. Mead M. E. Steenwyk J. L. Raja H. A. Oberlies N. H. (2020). Biosynthetic gene clusters and the evolution of fungal chemodiversity. Nat. Prod. Rep. 37, 868–878. 10.1039/c9np00045c 31898704 PMC7332410

[B223] Rossman A. Y. (2009). The impact of invasive fungi on agricultural ecosystems in the United States, in Ecological Impacts of Non-Native Invertebrates and Fungi on Terrestrial Ecosystems, eds Langor D. W. Sweeney J. (Dordrecht: Springer Netherlands), 97–107. 10.1007/s10530-008-9326-y PMC7088337 32214880

[B224] Rouphael Y. Colla G. (2020). Editorial: biostimulants in agriculture. Front. Plant Sci. 11:40. 10.3389/fpls.2020.00040 32117379 PMC7010726

[B225] Rubio M. B. Quijada N. M. Pérez E. Domínguez S. Monte E. Hermosa R. (2014). Identifying beneficial qualities of 831 *Trichoderma parareesei* for plants. Appl. Environ. Microbiol. 80, 1864–1873. 10.1128/AEM.03375-13 24413597 PMC3957631

[B226] Rychen G. Aquilina G. Azimonti G. Bampidis V. Bastos M. de L. . (2018). Safety and efficacy of muramidase from *Trichoderma reesei* DSM 32338 as a feed additive for chickens for fattening and minor poultry species. EFSA J. 16:5342. 10.2903/j.efsa.2018.5342 32625978 PMC7009659

[B227] Saari S. Faeth S. H. (2012). Hybridization of *Neotyphodium* endophytes enhances competitive ability of the host grass. New Phytol. 195, 231–236. 10.1111/j.1469-8137.2012.04140.x 22489964

[B228] Sánchez-Torres P. González R. Pérez-González J. A. González-Candelas L. Ramón D. (1994). Development of a transformation system for *Trichoderma longibrachiatum* and its use for constructing multicopy transformants for theegl1 gene. Appl. Microbiol. Biotechnol. 41, 440–446. 10.1007/bf01982533 7765105

[B229] Schardl C. L. Craven K. D. (2003). Interspecific hybridization in plant-associated fungi and oomycetes: a review. Mol. Ecol. 12, 2861–2873. 10.1046/j.1365-294X.2003.01965.x 14629368

[B230] Schenke D. Böttcher C. Lee J. Scheel D. (2011). Verticillin A is likely not produced by *Verticillium* sp. J. Antibiot. 64, 523–524. 10.1038/ja.2011.36 21522159

[B231] Schmitt I. Lumbsch H. T. (2009). Ancient horizontal gene transfer from bacteria enhances biosynthetic capabilities of fungi. PLoS ONE 4:e4437. 10.1371/journal.pone.0004437 19212443 PMC2636887

[B232] Schmoll M. Schuster A. (2010). Biology and biotechnology of *Trichoderma*. Appl. Microbiol. Biotechnol. 87, 787–799. 10.1007/s00253-010-2632-1 20461510 PMC2886115

[B233] Schoch C. L. Sung G. H. López-Giráldez F. Townsend J. P. Miadlikowska J. Hofstetter V. . (2009). The ascomycota tree of life: a phylum-wide phylogeny clarifies the origin and evolution of fundamental reproductive and ecological traits. Syst. Biol. 58, 224–239. 10.1093/sysbio/syp020 20525580

[B234] Schrimpe-Rutledge A. C. Codreanu S. G. Sherrod S. D. McLean J. A. (2016). Untargeted metabolomics strategies—challenges and emerging directions. J. Am. Soc. Mass Spectrom. 27, 1897–1905. 10.1007/s13361-016-1469-y 27624161 PMC5110944

[B235] Schroers H. J. Samuels G. J. Seifert K. A. Gams W. (1999). Classification of the mycoparasite *Gliocladium roseum* in *Clonostachys* as *C. rosea*, its relationship to Bionectria ochroleuca, and notes on other *Gliocladium*-like fungi. Mycologia 91, 365–385. 10.2307/3761383

[B236] Seidl V. Seibel C. Kubicek C. P. Schmoll M. (2009). Sexual development in the industrial workhorse *Trichoderma reesei*. Proc. Natl. Acad. Sci. U.S.A. 106, 13909–13914. 10.1073/pnas.0904936106 19667182 PMC2728994

[B237] Shenouda M. L. Cox R. J. (2021). Molecular methods unravel the biosynthetic potential of: *Trichoderma* species. RSC Adv. 11, 3622–3635. 10.1039/d0ra09627j 35424278 PMC8694227

[B238] Shi M. Chen L. Wang X. W. Zhang T. Zhao P. B. Song X. Y. . (2012). Antimicrobial peptaibols from *Trichoderma pseudokoningii* induce programmed cell death in plant fungal pathogens. Microbiology 158, 166–175. 10.1099/mic.0.052670-0 22053006

[B239] Shin J. Kim J. E. Lee Y. W. Son H. (2018). Fungal cytochrome p450s and the p450 complement (Cypome) of *Fusarium graminearum*. Toxins 10:112. 10.3390/toxins10030112 29518888 PMC5869400

[B240] Sieber C. M. K. Lee W. Wong P. Münsterkötter M. Mewes H. W. Schmeitzl C. . (2014). The *Fusarium graminearum* genome reveals more secondary metabolite gene clusters and hints of horizontal gene transfer. PLoS ONE 9:e110311. 10.1371/journal.pone.0110311 25333987 PMC4198257

[B241] Singh J. Yadav A. N. (2020). Natural Bioactive Products in Sustainable Agriculture. Singapore: Springer Nature.

[B242] Singh S. B. Liu W. Li X. Chen T. Shafiee A. Card D. . (2012). Antifungal spectrum, *in vivo* efficacy, and structure-activity relationship of ilicicolin H. ACS Med. Chem. Lett. 3, 814–817. 10.1021/ml300173e 24900384 PMC4025731

[B243] Singh S. B. Zink D. L. Goetz M. A. Dombrowski A. W. Polishook J. D. Hazuda D. J. (1998). Equisetin and a novel opposite stereochemical homolog phomasetin, two fungal metabolites as inhibitors of HIV-1 integrase. Tetrahedron Lett. 39, 2243–2246. 10.1016/S0040-4039(98)00269-X

[B244] Singh N. K. Blachowicz A. Romsdahl J. Wang C. Torok T. Venkateswaran K. (2017). Draft genome sequences of several fungal strains selected for exposure to microgravity at the international space station. Genome Announc. 5:e01602-16. 10.1128/genomeA.01602-16 28408692 PMC5391430

[B245] Sinha S. Nge C. E. Leong C. Y. Ng V. Crasta S. Alfatah M. . (2019). Genomics-driven discovery of a biosynthetic gene cluster required for the synthesis of BII-Rafflesfungin from the fungus *Phoma* sp. F3723. BMC Genomics 20, 1–18. 10.1186/s12864-019-5762-6 31088369 PMC6518819

[B246] Slightom J. L. Metzger B. P. Luu H. T. Elhammer A. P. (2009). Cloning and molecular characterization of the gene encoding the Aureobasidin A biosynthesis complex in *Aureobasidium pullulans* BP-1938. Gene 431, 67–79. 10.1016/j.gene.2008.11.011 19084058

[B247] Slot J. C. Rokas A. (2011). Horizontal transfer of a large and highly toxic secondary metabolic gene cluster between fungi. Curr. Biol. 21, 134–139. 10.1016/j.cub.2010.12.020 21194949

[B248] Smedsgaard J. Nielsen J. (2005). Metabolite profiling of fungi and yeast: from phenotype to metabolome by MS and informatics. J. Exp. Botany 56, 273–286. 10.1093/jxb/eri068 15618299

[B249] Snarr B. D. Baker P. Bamford N. C. Sato Y. Liu H. Lehoux M. . (2017). Microbial glycoside hydrolases as antibiofilm agents with cross-kingdom activity. Proc. Natl. Acad. Sci. U.S.A. 114, 7124–7129. 10.1073/pnas.1702798114 28634301 PMC5502622

[B250] Song Y. P. Miao F. P. Fang S. T. Yin X. L. Ji N. Y. (2018). Halogenated and nonhalogenated metabolites from the marine-alga-endophytic fungus *Trichoderma asperellum* CF44-2. Mar. Drugs 16:266. 10.3390/md16080266 30072624 PMC6117674

[B251] Sood M. Kapoor D. Kumar V. Sheteiwy M. S. Ramakrishnan M. Landi M. . (2020). *Trichoderma*: the “secrets” of a multitalented biocontrol agent. Plants 9, 1–25. 10.3390/plants9060762 32570799 PMC7355703

[B252] Souza A. D. L. Rodrigues-Filho E. Souza A. Q. L. Pereira J. O. Calgarotto A. K. Maso V. . (2008). Koninginins, phospholipase A2 inhibitors from endophytic fungus *Trichoderma koningii*. Toxicon 51, 240–250. 10.1016/j.toxicon.2007.09.009 17983638

[B253] Spatafora J. W. Aime M. C. Grigoriev I. V. Martin F. Stajich J. E. Blackwell M. (2017). The fungal tree of life: from molecular systematics to genome-scale phylogenies. Microbiol. Spectr. 5. 10.1128/microbiolspec.funk-0053-2016 28917057 PMC11687545

[B254] Spohn R. Daruka L. Lázár V. Martins A. Vidovics F. Grézal G. . (2019). Integrated evolutionary analysis reveals antimicrobial peptides with limited resistance. Nat. Commun. 10, 1–13. 10.1038/s41467-019-12364-6 31586049 PMC6778101

[B255] Stajich J. E. (2017). Fungal genomes and insights into the evolution of the Kingdom. Microbiol. Spectr. 5:2016. 10.1128/microbiolspec.funk-0055-2016 28820125 PMC6078396

[B256] Stark L. A. (2010). Beneficial microorganisms: countering microbephobia. CBE Life Sci. Educ. 9, 387–389. 10.1187/cbe.10-09-0119 21123679 PMC2995750

[B257] Stasz A. T. E. Harman G. E. Weeden N. F. (1988). Protoplast preparation and fusion in two biocontrol strains of Trichoderma harzianum. Mycologia 80, 141–150. 10.2307/3807788

[B258] Stasz T. E. (1990). Genetic improvement of fungi by protoplast fusion for biological control of plant pathogens. Can. J. Plant Pathol. 12, 322–327. 10.1080/07060669009501007

[B259] Stipanovic R. D. Howell C. R. (1982). The structure of gliovirin, a new antibiotic from *Gliocladium virens*. J. Antibiot. 35, 1326–1330. 10.7164/antibiotics.35.1326 6890954

[B260] Stoppacher N. Neumann N. K. N. Burgstaller L. Zeilinger S. Degenkolb T. Brückner H. . (2013). The comprehensive peptaibiotics database. Chem. Biodivers. 10, 734–743. 10.1002/cbdv.201200427 23681723

[B261] Studholme D. J. Harris B. Le Cocq K. Winsbury R. Perera V. Ryder L. . (2013). Investigating the beneficial traits of *Trichoderma hamatum* GD12 for sustainable agriculture-insights from genomics. Front. Plant Sci. 4:258. 10.3389/fpls.2013.00258 23908658 PMC3726867

[B262] Stukenbrock E. H. (2016). The role of hybridization in the evolution and emergence of new fungal plant pathogens. Phytopathology 106, 104–112. 10.1094/PHYTO-08-15-0184-RVW 26824768

[B263] Sun J. Z. Liu X. Z. McKenzie E. H. C. Jeewon R. Liu (Jack) J. K. Zhang X. L. . (2019). Fungicolous fungi: terminology, diversity, distribution, evolution, and species checklist. Fungal Divers. 95, 337–430. 10.1007/s13225-019-00422-9

[B264] Suzuki T. Watanabe S. Kobayashi S. Tanino K. (2017). Enantioselective total synthesis of (+)-Iso-A82775C, a proposed biosynthetic precursor of chloropupukeananin. Org. Lett. 19, 922–925. 10.1021/acs.orglett.7b00085 28128567

[B265] Swain H. Adak T. Mukherjee A. K. Mukherjee P. K. Bhattacharyya P. Behera S. . (2018). Novel *Trichoderma* strains isolated from tree barks as potential biocontrol agents and biofertilizers for direct seeded rice. Microbiol. Res. 214, 83–90. 10.1016/j.micres.2018.05.015 30031485

[B266] Szymańska M. Karakulska J. Sobolewski P. Kowalska U. Grygorcewicz B. Böttcher D. . (2020). Glycoside hydrolase (PelAh) immobilization prevents *Pseudomonas aeruginosa* biofilm formation on cellulose-based wound dressing. Carbohydr. Polym. 246:116625. 10.1016/j.carbpol.2020.116625 32747262

[B267] Tan X. Hu Y. Jia Y. Hou X. Xu Q. Han C. . (2020). A conserved glycoside hydrolase family 7 cellobiohydrolase PsGH7a of *Phytophthora sojae* is required for full virulence on soybean. Front. Microbiol. 11:1285. 10.3389/fmicb.2020.01285 32714289 PMC7343703

[B268] Te'o V. S. J. Bergquist P. L. Nevalainen K. M. H. (2002). Biolistic transformation of *Trichoderma reesei* using the Bio-Rad seven barrels Hepta Adaptor system. J. Microbiol. Methods 51, 393–399. 10.1016/S0167-7012(02)00126-4 12223300

[B269] Thambugala K. M. Daranagama D. A. Phillips A. J. L. Kannangara S. D. Promputtha I. (2020). Fungi vs. fungi in biocontrol: an overview of fungal antagonists applied against fungal plant pathogens. Front. Cell. Infect. Microbiol. 10:718. 10.3389/fcimb.2020.604923 33330142 PMC7734056

[B270] Tijerino A. Elena Cardoza R. Moraga J. Malmierca M. G. Vicente F. Aleu J. . (2011). Overexpression of the trichodiene synthase gene tri5 increases trichodermin production and antimicrobial activity in *Trichoderma brevicompactum*. Fungal Genet. Biol. 48, 285–296. 10.1016/j.fgb.2010.11.012 21145409

[B271] Toyomasu T. Tsukahara M. Kaneko A. Niida R. Mitsuhashi W. Dairi T. . (2007). Fusicoccins are biosynthesized by an unusual chimera diterpene synthase in fungi. Proc. Natl. Acad. Sci. U.S.A. 104, 3084–3088. 10.1073/pnas.0608426104 17360612 PMC1805559

[B272] Tran P. N. Yen M. R. Chiang C. Y. Lin H. C. Chen P. Y. (2019). Detecting and prioritizing biosynthetic gene clusters for bioactive compounds in bacteria and fungi. Appl. Microbiol. Biotechnol. 103, 3277–3287. 10.1007/s00253-019-09708-z 30859257 PMC6449301

[B273] Triolet M. Guillemin J. P. Andre O. Steinberg C. (2020). Fungal-based bioherbicides for weed control: a myth or a reality? Weed Res. 60, 60–77. 10.1111/wre.12389

[B274] Urbán P. Miao Y. Fekete C. Hatvani L. Büchner R. Vágvölgyi C. . (2016). Complete genome sequence of the green mould pathogen, *Trichoderma pleuroti*, in 18th Danube-Kris-Mures-Tisa (DMKT) Euroregional Conference on Environment and Health, Book of Abstracts, ed Škrbić B. (Novi Sad: University of Novi Sad), 51–52.

[B275] van den Bosch R. Messenger P. S. Gutierrez A. P. (1982). The history and development of biological control, in An Introduction to Biological Control, eds Gutierrez A. P. Messenger P. S. van den Bosch R. (New York, NY: Springer US), 21–36.

[B276] van Lenteren J. C. Bolckmans K. Köhl J. Ravensberg W. J. Urbaneja A. (2018). Biological control using invertebrates and microorganisms: plenty of new opportunities. BioControl 63, 39–59. 10.1007/s10526-017-9801-4

[B277] Vargas Gil S. Pastor S. March G. J. (2009). Quantitative isolation of biocontrol agents *Trichoderma* spp., *Gliocladium* spp. and actinomycetes from soil with culture media. Microbiol. Res. 164, 196–205. 10.1016/j.micres.2006.11.022 17459686

[B278] Venice F. Davolos D. Spina F. Poli A. Prigione V. P. Varese G. C. . (2020). Genome sequence of *Trichoderma lixii* mut3171, a promising strain for mycoremediation of pah-contaminated sites. Microorganisms 8, 1–15. 10.3390/microorganisms8091258 32825267 PMC7570066

[B279] Verma M. Brar S. K. Tyagi R. D. Surampalli R. Y. Valéro J. R. (2007). Antagonistic fungi, *Trichoderma* spp.: panoply of biological control. Biochem. Eng. J. 37, 1–20. 10.1016/j.bej.2007.05.012

[B280] Vicente I. Baroncelli R. Morán-Diez M. E. Bernardi R. Puntoni G. Hermosa R. . (2020). Combined comparative genomics and gene expression analyses provide insights into the terpene synthases inventory in *Trichoderma*. Microorganism 8, 1–20. 10.3390/microorganisms8101603 33081019 PMC7603203

[B281] Vinale F. Sivasithamparam K. Ghisalberti E. L. Marra R. Barbetti M. J. Li H. . (2008a). A novel role for *Trichoderma* secondary metabolites in the interactions with plants. Physiol. Mol. Plant Pathol. 72, 80–86. 10.1016/j.pmpp.2008.05.005

[B282] Vinale F. Sivasithamparam K. Ghisalberti E. L. Marra R. Woo S. L. Lorito M. (2008b). *Trichoderma*-plant-pathogen interactions. Soil Biol. Biochem. 40, 1–10. 10.1016/j.soilbio.2007.07.002

[B283] Vincelli P. (2012). Q(o)I (strobilurin) fungicides: benefits and risks. Plant Heal. Instr. 10.1094/phi-i-2002-0809-02. [Epub ahead of print].

[B284] Von Bargen K. W. Niehaus E. M. Krug I. Bergander K. Würthwein E. U. Tudzynski B. . (2015). Isolation and structure elucidation of Fujikurins A-D: products of the PKS19 Gene Cluster in *Fusarium fujikuroi*. J. Nat. Prod. 78, 1809–1815. 10.1021/np5008137 26192387

[B285] Wada S. ichi Iida A. Asami K. Fujita T. (1996). Ion channel-forming property of trichorovin-XII, an 11-residue peptaibol from the fungus *Trichoderma viride*, in planar lipid bilayer membranes. Bioorganic Med. Chem. Lett. 6, 2275–2278. 10.1016/0960-894X(96)00410-6

[B286] Wang C. C. C. Chiang Y. M. Praseuth M. B. Kuo P. L. Liang H. L. Hsu Y. L. (2010). Asperfuranone from *Aspergillus nidulans* inhibits proliferation of human non-small cell lung cancer A549 cells via blocking cell cycle progression and inducing apoptosis. Basic Clin. Pharmacol. Toxicol. 107, 583–589. 10.1111/j.1742-7843.2010.00545.x 20148857 PMC3110816

[B287] Wang G. Li X. Wang Z. (2016). APD3: The antimicrobial peptide database as a tool for research and education. Nucleic Acids Res. 44, D1087–D1093. 10.1093/nar/gkv1278 26602694 PMC4702905

[B288] Wang X. Peng J. Sun L. Bonito G. Guo Y. Li Y. . (2020). Genome sequencing of *Paecilomyces penicillatus* provides insights into its phylogenetic placement and mycoparasitism mechanisms on morel mushrooms. Pathogens 9, 1–12. 10.3390/pathogens9100834 33065983 PMC7650745

[B289] Wanka F. (2021). Open the pores: electroporation for the transformation of *Trichoderma reesei*, in Methods in Molecular Biology, eds Mach-Aigner A. R. Martzy R. (New York, NY: Humana Press Inc.), 73–78. 10.1007/978-1-0716-1048-0_6 33165780

[B290] Wasil Z. Pahirulzaman K. A. K. Butts C. Simpson T. J. Lazarus C. M. Cox R. J. (2013). One pathway, many compounds: heterologous expression of a fungal biosynthetic pathway reveals its intrinsic potential for diversity. Chem. Sci. 4, 3845–3856. 10.1039/c3sc51785c

[B291] Wee J. L. Sundermann K. Licari P. Galazzo J. (2006). Cytotoxic hypothemycin analogues from *Hypomyces subiculosus*. J. Nat. Prod. 69, 1456–1459. 10.1021/np060258o 17067161

[B292] Whipps J. M. (2001). Microbial interactions and biocontrol in the rhizosphere. J. Exp. Bot. 52, 487–511. 10.1093/jexbot/52.suppl_1.487 11326055

[B293] Woo P. C. Y. Lam C. W. Tam E. W. T. Lee K. C. Yung K. K. Y. Leung C. K. F. . (2014). The biosynthetic pathway for a thousand-year-old natural food colorant and citrinin in *Penicillium marneffei*. Sci. Rep. 4, 1–8. 10.1038/srep06728 25335861 PMC4205486

[B294] Xiao-Yan S. Qing-Tao S. Shu-Tao X. Xiu-Lan C. Cai-Yun S. Yu-Zhong Z. (2006). Broad-spectrum antimicrobial activity and high stability of Trichokonins from *Trichoderma koningii* SMF2 against plant pathogens. FEMS Microbiol. Lett. 260, 119–125. 10.1111/j.1574-6968.2006.00316.x 16790027

[B295] Xie B. B. Qin Q. L. Shi M. Chen L. L. Shu Y. L. Luo Y. . (2014). Comparative genomics provide insights into evolution of *Trichoderma* nutrition style. Genome Biol. Evol. 6, 379–390. 10.1093/gbe/evu018 24482532 PMC3942035

[B296] Xu Y. Vinas M. Alsarrag A. Su L. Pfohl K. Rohlfs M. . (2019). Bis-naphthopyrone pigments protect filamentous ascomycetes from a wide range of predators. Nat. Commun. 10, 1–12. 10.1038/s41467-019-11377-5 31395863 PMC6687722

[B297] Xu Z. Escamilla-Treviño L. L. Zeng L. Lalgondar M. Bevan D. R. Winkel B. S. J. . (2004). Functional genomic analysis of *Arabidopsis thaliana* glycoside hydrolase family 1. Plant Mol. Biol. 55, 343–367. 10.1007/s11103-004-0790-1 15604686

[B298] Xu J. Li F. Leier A. Xiang D. Shen H.-H. Marquez Lago T. T. . (2021). Comprehensive assessment of machine learning-based methods for predicting antimicrobial peptides. Brief. Bioinform. 10.1093/bib/bbab083. [Epub ahead of print]. 33774670

[B299] Yabuta T. Sumiki Y. Aso K. Tamura T. Igarashi H. Tamari K. (1939). Biochemical studies on the bakanae fungus. IV. The culture conditions for producing gibberellin or fusaric acid. J. Agric. Chem. Soc. Jpn. 15, 1209–1220.

[B300] Yamashita A. Yoshizawa T. Aiura Y. Sanchez P. C. Dizon E. I. Arim R. H. . (1995). *Fusarium* mycotoxins (Fumonisins, Nivalenol, and Zearalenone) and aflatoxins in corn from Southeast Asia. Biosci. Biotechnol. Biochem. 59, 1804–1807. 10.1271/bbb.59.1804 8520126

[B301] Yan Y. Liu Q. Jacobsen S. E. Tang Y. (2018). The impact and prospect of natural product discovery in agriculture. EMBO Rep. 19:e46824. 10.15252/embr.201846824 30361392 PMC6216283

[B302] Yang D. Pomraning K. Kopchinskiy A. Aghcheh R. K. Atanasova L. Chenthamara K. . (2015). Genome sequence and annotation of *Trichoderma parareesei*, the ancestor of the cellulase producer *Trichoderma reesei*. Genome Announc. 3, 885–900. 10.1128/genomeA.00885-15 26272569 PMC4536680

[B303] Yang L. Yang Q. Sun K. Tian Y. Li H. (2011). *Agrobacterium tumefaciens* mediated transformation of ChiV gene to *Trichoderma harzianum*. Appl. Biochem. Biotechnol. 163, 937–945. 10.1007/s12010-010-9097-7 20936373

[B304] Zeilinger S. (2004). Gene disruption in *Trichoderma atroviride* via *Agrobacterium*-mediated transformation. Curr. Genet. 45, 54–60. 10.1007/s00294-003-0454-8 14586554

[B305] Zeilinger S. Gruber S. Bansal R. Mukherjee P. K. (2016). Secondary metabolism in *Trichoderma*- chemistry meets genomics. Fungal Biol. Rev. 30, 74–90. 10.1016/j.fbr.2016.05.001

[B306] Zhang L. Yan J. Fu Z. Shi W. Ninkuu V. Li G. . (2021). FoEG1, a secreted glycoside hydrolase family 12 protein from *Fusarium oxysporum*, triggers cell death and modulates plant immunity. Mol. Plant Pathol. 22, 522–538. 10.1111/mpp.13041 33675158 PMC8035634

[B307] Zhang Q. W. Lin L. G. Ye W. C. (2018a). Techniques for extraction and isolation of natural products: a comprehensive review. Chinese Med. 13:20. 10.1186/s13020-018-0177-x 29692864 PMC5905184

[B308] Zhang S. Gan Y. Ji W. Xu B. Hou B. Liu J. (2017). Mechanisms and characterization of *Trichoderma longibrachiatum* T6 in suppressing nematodes (*Heterodera avenae*) in wheat. Front. Plant Sci. 8:1491. 10.3389/fpls.2017.01491 28966623 PMC5605630

[B309] Zhang S. Gan Y. Xu B. (2015). Biocontrol potential of a native species of *Trichoderma longibrachiatum* against *Meloidogyne incognita*. Appl. Soil Ecol. 94, 21–29. 10.1016/j.apsoil.2015.04.010

[B310] Zhang S. Xu B. Zhang J. Gan Y. (2018b). Identification of the antifungal activity of *Trichoderma longibrachiatum* T6 and assessment of bioactive substances in controlling phytopathgens. Pestic. Biochem. Physiol. 147, 59–66. 10.1016/j.pestbp.2018.02.006 29933994

[B311] Zhang Y. Wang X. Pang G. Cai F. Zhang J. Shen Z. . (2019). Two-step genomic sequence comparison strategy to design *Trichoderma* strain-specific primers for quantitative PCR. AMB Express 9:179. 10.1186/s13568-019-0904-4 31707479 PMC6842373

[B312] Zhang J. Xin L. Shan B. Chen W. Xie M. Yuen D. . (2012). PEAKS DB: *de novo* sequencing assisted database search for sensitive and accurate peptide identification. Mol. Cell. Proteomics 11:M111.010587. 10.1074/mcp.M111.010587 22186715 PMC3322562

[B313] Zhao Y. Ding J. Yuan W. Huang J. Huang W. Wang Y. . (2017). Production of a fungal furocoumarin by a polyketide synthase gene cluster confers the chemo-resistance of *Neurospora crassa* to the predation by fungivorous arthropods. Environ. Microbiol. 19, 3920–3929. 10.1111/1462-2920.13791 28485098

[B314] Zheng P. Xia Y. Xiao G. Xiong C. Hu X. Zhang S. . (2011). Genome sequence of the insect pathogenic fungus *Cordyceps militaris*, a valued traditional chinese medicine. Genome Biol. 12:R116. 10.1186/gb-2011-12-11-r116 22112802 PMC3334602

[B315] Zhong Y. H. Wang X. L. Wang T. H. Jiang Q. (2007). *Agrobacterium*-mediated transformation (AMT) of *Trichoderma reesei* as an efficient tool for random insertional mutagenesis. Appl. Microbiol. Biotechnol. 73, 1348–1354. 10.1007/s00253-006-0603-3 17021875

[B316] Zhou Y. Wang Y. Chen K. Wu Y. Hu J. Wei Y. . (2020). Near-complete genomes of two *Trichoderma* species: a resource for biological control of plant pathogens. Mol. Plant Microbe Interact. 33, 1036–1039. 10.1094/MPMI-03-20-0076-A 32314945

[B317] Zhu Z. X. Zhuang W. Y. (2015). Three new species of *Trichoderma* with hyaline ascospores from China. Mycologia 107, 328–345. 10.3852/14-141 25572101

